# Synthesis and characterization of monodispersed water dispersible Fe_3_O_4_ nanoparticles and *in vitro* studies on human breast carcinoma cell line under hyperthermia condition

**DOI:** 10.1038/s41598-018-32934-w

**Published:** 2018-10-03

**Authors:** K. S. Sharma, R. S. Ningthoujam, A. K. Dubey, A. Chattopadhyay, S. Phapale, R. R. Juluri, S. Mukherjee, R. Tewari, Neena G. Shetake, B. N. Pandey, R. K. Vatsa

**Affiliations:** 1 Chemistry Division, Bhabha Atomic Research Centre, Mumbai, 400085 India; 2 Bio-organic Division, Bhabha Atomic Research Centre, Mumbai, 400085 India; 3 Institute of Physics, SachivalayaMarg, Bhubaneswar, 751005 India; 4 UGC-DAE Consortium for Scientific Research, Mumbai Centre, Mumbai, 400085 India; 5 Material Science Division, Bhabha Atomic Research Centre, Mumbai, 400085 India; 6 Radiation Biology and Health Sciences Division, Bhabha Atomic Research Centre, Mumbai, 400085 India

## Abstract

Monodispersed Fe_3_O_4_ magnetic nanoparticles (MNPs) having size of 7 nm have been prepared from iron oleate and made water dispersible by functionalization for biomedical applications. Three different reactions employing thioglycolic acid, aspartic acid and aminophosphonate were performed on oleic acid coated Fe_3_O_4_. In order to achieve a control on particle size, the pristine nanoparticles were heated in presence of ferric oleate which led to increase in size from 7 to 11 nm. Reaction parameters such as rate of heating, reaction temperature and duration of heating have been studied. Shape of particles was found to change from spherical to cuboid. The cuboid shape in turn enhances magneto-crystalline anisotropy (*K*_*u*_). Heating efficacy of these nanoparticles for hyperthermia was also evaluated for different shapes and sizes. We demonstrate heat generation from these MNPs for hyperthermia application under alternating current (AC) magnetic field and optimized heating efficiency by controlling morphology of particles. We have also studied intra-cellular uptake and localization of nanoparticles and cytotoxicity under AC magnetic field in human breast carcinoma cell line.

## Introduction

Magnetic nanoparticles (MNPs) have been used for biomedical applications such as drug delivery vehicles, magnetic fluid hyperthermia, separating agents for biomolecules and magnetic resonance imaging (MRI) contrast agent^1–5^. For these applications, crystalline, size optimized and non-agglomerated nanoparticles are required^6^. Amongst MNPs, Fe_3_O_4_ particles are very important materials since they are biocompatible up to large concentrations. Fe_3_O_4_ MNPs can be synthesized using several different methods. The most commonly used is co-precipitation method in aqueous medium. However, the particles prepared by this method suffer from agglomeration and poor size control^7^, thereby being not very useful for biomedical application. Preparation of MNPs through reverse micelle is another commonly used method employed to reduce agglomeration^8^, however, in this case the crystallinity is poor and within a few days, agglomeration sets in. The best way to synthesize monodispersed and non-agglomerated particles is thermolysis or hot injection method which utilizes solvents having high boiling point and capping agent with long chain fatty acid. The main disadvantage of this method for biomedical applications is that the synthesized particles are not soluble (form suspension) in polar solvent like water^9–15^. Different strategies have been developed to make MNPs coated with long fatty acids water dispersible. One way is exchanging ligands with more hydrophilic ligands. But due to small chain length, there is a least separation between the particles and the particles settle down due to aggloromoration^15^. Another way is coating the particle with silica shell, dendrimers or polyethylene glycol (PEG) but still they suffer from agglomeration of particles. Another drawback is that the reaction procedures are lengthy^16–18^.

Another important issue from application point of view is the blocking temperature (*T*_*B*_, the ferromagnetic to superparamagnetic transition temperature). In most of the earlier reports on Fe_3_O_4_ MNPs, *T*_*B*_ is much lower than room temperature. It is desirable to prepare MNPs having *T*_*B*_ near to room temperature or normal body temperature (37 °C). Such properties make these nanoparticles to generate heat sufficient to kill cancer cells efficiently in a short period of time while applying a lower magnetic field at lower frequency and lower concentration of MNPs. So far, these issues have not been addressed in the literature.

In the present work, we report preparation of monodispersed Fe_3_O_4_ MNPs from Fe (III) oleate, which was produced by less expensive reagents Fe (III) salt and oleic acid instead of using expensive reagents Fe(II)/Fe(III) pentacarbonyl or Fe (III) acetylacetonate or sodium oleate. To make these MNPs water soluble, we have focused on addition and cleavage reactions on the double bond of oleic acid coating. A combination of these properties can make nanoparticles useful so that targeting and mapping can be carried out. Further, we show that shape and size of these particles can be manipulated to get desired heat generation under applied alternating current (AC) magnetic field for the hyperthermia treatment. We have studied the interaction of MNPs with human breast carcinoma cells and their killing by MNPs under AC magnetic field. This suggests that the water dispersible Fe_3_O_4_ MNPs will be a potential candidate material for application in magnetic based hyperthermia therapy.

## Results and Discussion

### XRD, FT-IR and TGA-DTA studies

**Figure 1:**
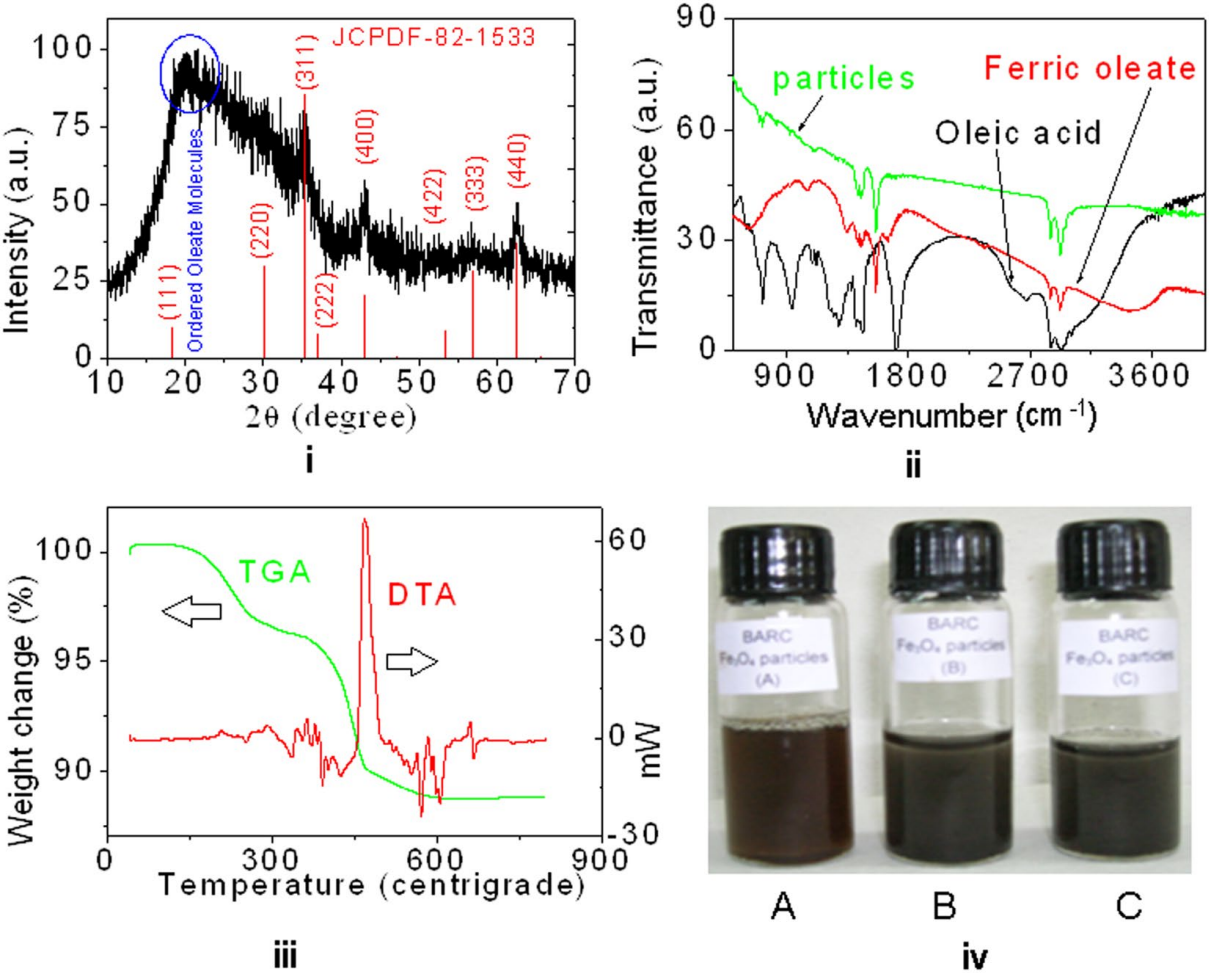
(**i**) XRD pattern of Fe_3_O_4_ particles along with JCPDF file, (**ii**) FT-IR spectra of oleic acid, ferric oleate and Fe_3_O_4_ particles, (**iii**) TGA and DTA curves of Fe_3_O_4_ particles, and (**iv**) Water dispersible Fe_3_O_4_ particles functionalized by (A) thioglycolic acid, (B) aspartic acid and (**C**) aminophosphonate.

The X-ray diffraction (XRD) pattern of as synthesized oleic acid coated Fe_3_O_4_ particles is shown in Fig. 1(i). The pattern of the sample matches with the cubic phase of Fe_3_O_4_ (JCPDF – 82–1533). The lattice parameter of the particles is found to be *a* = 8.385 Å. There is a broad peak around 2*θ* = 20°, which shows the ordered nature of oleate molecules present in the particles, but glass substrate used for XRD recording shows a broad peak at 2*θ* = 22–23°. Fe_3_O_4_ coated with polymers having a broad peak at 2*θ* = 20° was reported due to the formation of semi-crystalline phase^19^. The *d*- spacing of the ordered oleate particles is found to be 4.3 Å. It is to be noted that in the literature, some researchers have reported XRD pattern of the oleic acid coated nanoparticles after removing oleic acid, in which the broad peak corresponding to ordered oleates was not observed^20^. We believe that removing oleic acid may change morphologies and properties of materials. Our XRD patterns show that Fe_3_O_4_ particles have surface coated oleates.

Fourier transformed infrared (FT-IR) spectra of oleic acid, ferric oleate and Fe_3_O_4_ particles are shown in Fig. 1(ii). In case of oleic acid, peaks observed at 2890 and 2950 cm^−1^ correspond to CH_2_ stretching vibration and peak at 3059 cm^−1^ corresponds to =C-H stretching vibration of unsaturated carbons (C_9_-C_10_) of oleic acid^21^. The 1700 cm^−1^ peak corresponds to stretching vibration of C=O in oleic acid. The broad peak in 2200–3600 cm^−1^ corresponds to O-H stretching vibration of oleic acid in liquid phase. Ferric oleate has similar peaks of oleic acid, but the peak at 1700 cm^−1^ disappears and instead, the new peaks at 1557 and 1441 cm^−1^ appear and correspond to the anti-symmetric and symmetric stretching vibrations of carboxyl group (CO_2_^−^) attached to Fe^3+^ ions. The small peak at 3400 cm^−1^ corresponds to H_2_O present in oleate. In case of Fe_3_O_4_, extra peak at 607 cm^−1^ is observed as compared to oleate and this corresponds to Fe-O bond vibration. Detail studies are given in Supporting Information SI1.1 and 1.2.

Thermo-gravimetric analysis (TGA) curve of Fe_3_O_4_ particles is shown in Fig. 1(iii). Total contribution of oleate or oleic acid molecules is 24%. Similar findings were reported elsewhere^21,22^. Differential thermal analysis (DTA) curve of Fe_3_O_4_ shows three exothermic peaks at 350, 435 and 468 °C, which suggest the decomposition of oleate molecules.

### TEM study

**Figure 2:**
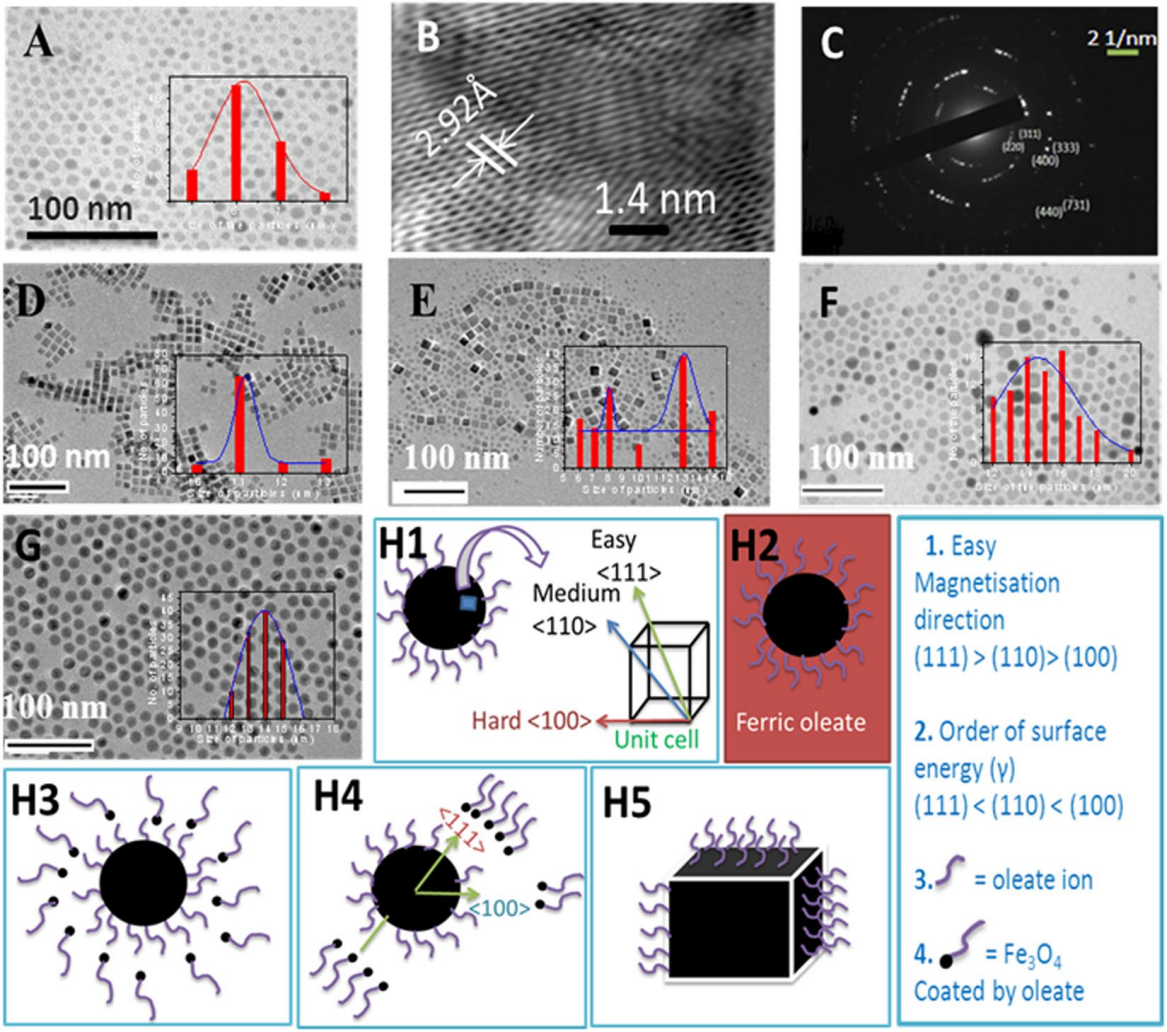
Core Fe_3_O_4_ particles (heating rate of 4 °C per minute): (**A**) TEM image, (**B**) HRTREM image, (**C**) SAED pattern. Core-shell Fe_3_O_4_@ Fe_3_O_4_ particles after addition of (**D**) 1 mL and (**E**) 5 mL of ferric oleate (duration of heating is 1 hour) and (**F**) 5 mL of ferric oleate (duration of heating is 8 hour). (**G**) core Fe_3_O_4_ particles prepared by heating rate of 1.3 °C/min. Schematic diagram of conversion of spherical to cubic shape of a particle by core-shell model (Ostwald ripening phenomenon): (**H1**) slightly spherical particle coated by oleate ions along with magnetisation axes of face-centred cube (Fe_3_O_4_ unit cell), (**H2**) particles in ferric oleate medium, (**H3**) formation of Fe_3_O_4_ small paricles/cluster which surrounds a larger particle, (**H4**) Faster growth/deposition along 〈111〉 direction as compared to 〈100〉 or 〈220〉 direction, (**H5**) formation of cubic shaped particle.

The size and morphology of Fe_3_O_4_ particles are investigated by transmission electron microscopy (TEM). TEM images reveal quasi-spherical particles with size of 7–8 nm when rate of heating during synthesis is maintained at 4 °C/min. The particle size distribution was determined by the statistical evaluation of ~100 particles (Fig. 2A). Selected area electron diffraction (SAED) patterns of the particles as well as the lattice fringes are observed in high resolution images. This is indicated that the particles are highly crystalline (Fig. 2B,C). From high resolution TEM image, *d* – spacing is found to be 2.92 Ǻ, which corresponds to (220) plane of cubic Fe_3_O_4_. These are treated as pristine Fe_3_O_4_ nanoparticles.

Adding ferric oleate (1 ml) to Fe_3_O_4_ particles (7 nm) followed by heating at 320 °C for 1 hour changes the shape of the particles from spherical to cuboid (Fig. 2D). The particles are monodispersed and are found to be 11 nm (one side of cuboid). In case of addition of 5 mL of ferric oleate to the already synthesized particles (following the same heating conditions as mentioned above), formation of large number of smaller particles around the bigger particles is observed as seen in Fig. 2E. The particle size shows bimodal distribution. Small spherical shaped particles of ~6–8 nm are found along with bigger cuboid particles with size of 13–16 nm.

However, when 5 mL of ferric oleate is added followed by 8 hours heating, particles are found to be larger in size (16 nm) and they contain both spherical as well as cuboid shapes (Fig. 2F). Also it was noticed that the smaller particles were not present. Detail study about formation of bigger size particles is given in SI2. Interestingly, highly monodispersed spherical Fe_3_O_4_ MNPs of bigger size (14 ± 1) are obtained when heated at a slower rate of 1.5 °C/min (i.e., instead of 4 °C/min) to reach 320 °C (Fig. 2G). As can be seen from the results, in order to prepare uniform spherical Fe_3_O_4_ particles, a very slow heating rate of 1.5 °C per minute is essential and also, different shape and size of Fe_3_O_4_ particles can be prepared using this method.

Increase in the size of the Fe_3_O_4_ particles can be explained based on Ostwald ripening (Fig. 2H), in which the smaller particles get deposited on the surface of larger particles to gain stability by lowering surface energy^23,24^. When ferric oleate solution is added to the pristine particles followed by heated to the reaction temperature for longer times, small particles are formed which subsequently get deposited on the already present larger particles. The larger particles act as nucleation sites for newly formed cluster/molecules. Change of shape of particle from spherical to cuboid can’t be explained purely on the basis of Ostwald ripening. It is obvious that some other factors also start playing important role in governing the shape of particles. A possible explanation is given below for transformation of spherical to cuboid shape. Upon heating the pristine spherical nanoparticles in presence of ferric oleate solution, newly formed smaller particles from ferric oleate preferably deposit on larger spherical nanoparticles on a specific plane that is easy to deposit and has less surface energy (*γ*). The sequence of planes in terms of surface energy is *γ* (111) < *γ* (100) < *γ* (110) < *γ* (220) in face-centered cube (fcc). Also, 〈111〉 direction has more magnetization than other directions in fcc Fe_3_O_4_ unit cell^25^. In such a situation, newly formed nanoparticles prefer to deposit on the plane (111) (i.e., growth rate is faster along 〈111〉 direction) and thus, the cubic shaped particles are formed, when amount of ferric oleate added is 1 mL. It has been reported that a faster growth rate along 〈111〉 direction over 〈100〉 direction leads to cubic shaped particles^25^. The formation steps are shown in Fig. 2H(1–5). Upon increasing the amount of ferric oleate to 5 ml, formation of a mixture of spherical and cuboid shaped particles was observed. This may be due to the inhomogeneous deposition of smaller particles over larger particles in terms of direction (〈111〉 or 〈100〉) when amount of newly formed smaller particles from ferric oleate is comparable to that of the larger particles already present.

### Functionalization of nanoparticles

Three different protocols are used here for making water dispersible, monodispersed Fe_3_O_4_ nanoparticles, which may have potential for application in drug delivery, hyperthermia treatment, etc.^26–32^. These protocols open an easy way to convert particles to another functional group so that it can be utilized for different applications (SI3). Amount of oleic acid on the surface of the particles and iron were calculated theoretically (see SI4) using the cubical or spherical model of the particles. The theoretically calculated value is in close agreement with the experimentally measured thermogravimetric analysis (TGA) value. The contribution of oleic acid or oleate in oleic acid coated Fe_3_O_4_ is about 24 wt.% in TGA. Exothermic reaction in differential thermal analysis (DTA) suggests evolution of different gases (e. g., CO_2_, CO, C_x_H_y_) which results into simultaneous oxidation and reduction reactions during formation of Fe_3_O_4_. Here, we use Fe^3+^ ions as precursor, whereas Fe_3_O_4_ contains Fe^2+^/Fe^3+^ ions. Part of Fe^3+^ is converted into Fe^2+^ (1:2 ratio of Fe^2+^:Fe^3+^ in Fe_3_O_4_). FT-IR study suggests that Fe_3_O_4_ is capped by COO^−^ group of oleate.

### Magnetization study

**Figure 3:**
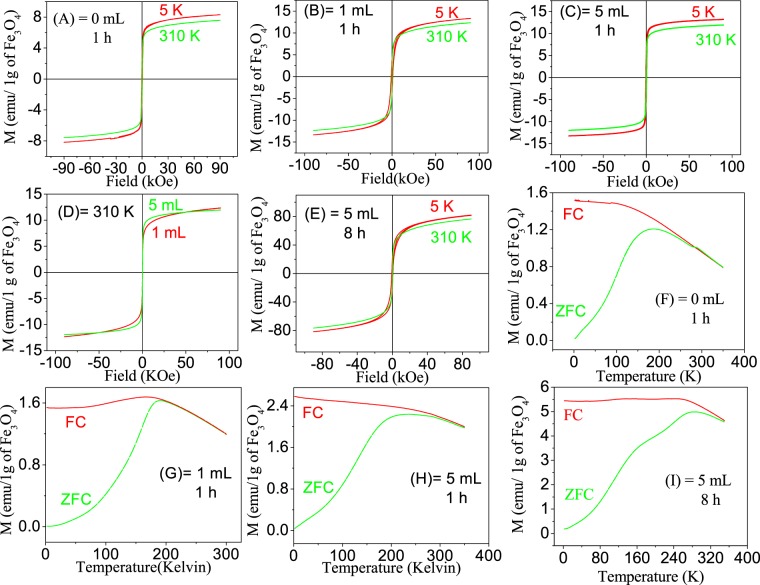
Magnetisation – Applied magnetic field (M-H) plots of particles. (**A**) Core Fe_3_O_4_, Core-shell Fe_3_O_4_@ Fe_3_O_4_ particles after addition of 1 mL of ferric oleate for 1 h duration of heating (**B**), 5 mL of ferric oleate for 1 h (**C**) and 5 mL of ferric oleate for 8 h (**E**). (**D**) M-H comparison at 310 K for 1 ml and 5 mL for 1 h. Their corresponding M-T plots in ZFC and FC modes (**F**–**I**).

Magnetization data of pristine particles (7–8 nm) with applied magnetic field (*M-H*) at two temperatures 5 and 310 K are shown in Fig. 3A. At 310 K (normal body temperature, 37 °C), there is no hysteresis loop indicating superparamagnetic nature of particles. At 5 K, there is a hysteresis loop (*M-H*) indicating ferromagnetic nature of particles with coercivity (*H*_*c*_ = 375 Oe), which is given in SI5. This suggests change of ferromagnetic to superparamagnetic with increasing temperature and this is supported by the blocking temperature (*T*_*B*_), which is found to be 181 K in zero field cooled - field cooled curves (ZFC–FC) measurement (Fig. 3F). Since *M* does not saturate up to 9 T, *M* vs. *1/H* is plotted. When *1/H* → 0, *M* can be considered as *M*_*s*_ (saturation magnetization). *M*_*s*_ values at 5 and 310 K are found to be 11.96 and 11.01 emu/g, respectively.

Similarly, *M-H* and *M-T* data for bigger particles, 1 and 5 mL for 1 h duration are shown in Fig. 3B,C,G,H, respectively. *T*_*B*_*, H*_*c*_*, M*_*S*_ (measured at 5 K) values of Fe_3_O_4_ MNPs (having 1 mL and 5 mL for 1 h heating) are 188 K, 910 Oe, 19.27 emu/g and 211 K, 386 Oe, 15.77 emu/g respectively. *T*_*B*_*, H*_*c*_ and *M*_*s*_ are improved by formation of bigger particles. *H*_*c*_ for 1 mL is more than that for 5 mL. For comparison, *M-H* plots of 1 mL and 5 mL at 310 K are shown in Fig. 3D. Rise of *M* at lower temperature is more in case of 5 mL than that of 1 mL. However, *T*_*B*_*, H*_*c*_ and *M*_*S*_ (measured at 5 K) of 5 mL for 8 h are 282 K, 688 Oe and 90 emu/g, respectively (Fig. 3E,I). These values increase with increasing duration of heating from 1 to 8 h. The *M*_*s*_ value for bulk Fe_3_O_4_ is 92 emu/g measured at 5 K with *H*_*c*_ of 736 Oe^21^. Details are given in SI5.

### Hyperthermia study

**Figure 4:**
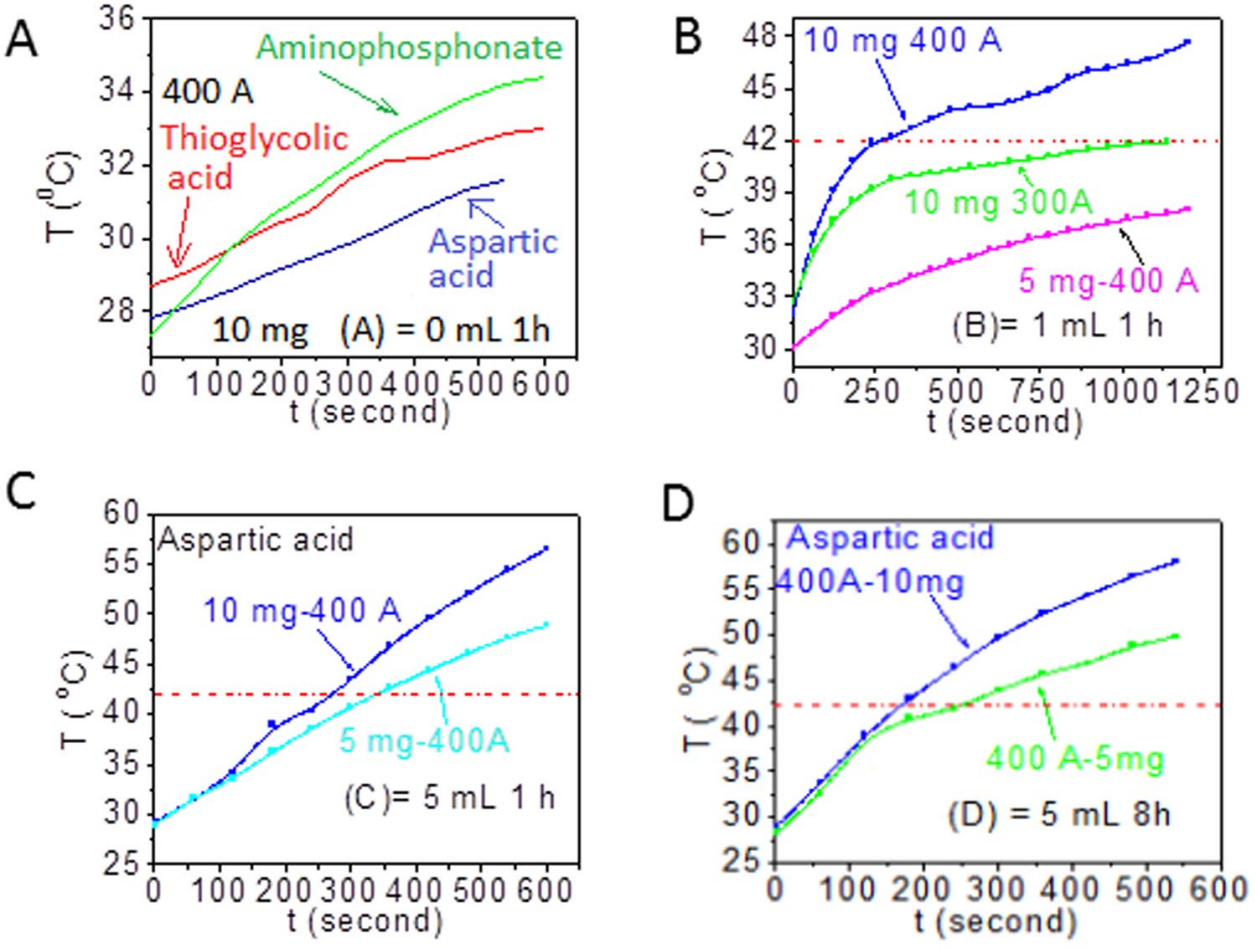
Heat released with respect to time under AC magnetic field (**A**) Core, Core-shell Fe_3_O_4_ @ Fe_3_O_4_ particles after addition of 1 mL of ferric oleate for 1 h duration of heating (**B**), 5 mL of ferric oleate for 1 h (**C**) and 5 mL of ferric oleate for 8 h (**D**). Dotted lines denote hyperthermia temperature with time. Particles are coated with aspartic acid. All samples are of MNPs-aspartic acid. However, MNPs-thioglycolic acid and MNPs-aminophosphonate acid are provided in (a) for comparison with MNPs-aspartic acid.

For typical demonstration of heating efficacy, the Fe_3_O_4_ particles coated with aspartic acid molecules are dispersed in water; and the dispersed particles (5 or 10 mg per 1 mL) are kept in induction coil (frequency = 265 kHz, current = 300 or 400A). 10 mg/mL of pristine Fe_3_O_4_ particles (7 nm) produce 31–33 °C within 600 seconds at 400 A (335 Oe) (Fig. 4(a)), but could not reach hyperthermia temperature (*HT* = 42 °C). Similarly, 5 mg/mL of bigger particles (cuboid shaped nanoparticles, 1 ml for 1 h) at 400 A could not reach *HT* (Fig. 4(b)). But, 10 mg/mL at 300 and 400A could reach HT in 1137 and 255 s, respectively. In another case (bigger particles, 5 mL for 1 h, 5 and 10 mg/ml could reach *HT* at 400 A (Fig. 4(c)) within 337 and 270 s, respectively. 5 and 10 mg/ml of the bigger particles (5 ml for 8 h case) reach *HT* at 400A at 245 and 170 s, respectively (Fig. 4(d)). These results suggest that the time required for reaching *HT* decreases with increasing particle size and magnetic field. Specific absorption rates (SAR) of pristine particles (0 mL, 1 h), bigger particles synthesized by (1 mL, 1 h), (5 mL, 1 h) and (5 mL, 8 h) are found to be 4.1, 42.7, 30.9 and 46.5 Wg^−1^, respectively and here, SAR is expressed in term of Watts per 1 g of Fe_3_O_4_. In our earlier studies^6,21^, SAR values of 30–40 Wg^−1^ for agglomerated Fe_3_O_4_ particles (size 10–12 nm) were found under similar conditions of magnetic field and AC frequency. Thus, change of morphologies has made a large change in magnetic properties. Considering nanoparticles of same volume, there is difference in magnetic anisotropy between the cuboid and spherical shaped nanoparticles. Coercivity of cuboid shaped particles is higher than that of spherical particles (see SI5). Cuboid shaped magnetic nanoparticles have large magnetic anisotropy constant (K) than the spherical shaped magnetic nanoparticles. The increase in heating in cuboid shaped particles is due to increase in magnetic anisotropy. SAR value increases by increase of particle size or change of shape from spherical to cuboid. Thus, magnetic nanoparticles will be useful in heat generation for hyperthermia based cancer therapy in economic ways (lower amount of materials, easy processing/dispersion, reduced current, time, etc.). The heat generation from magnetic fluid under AC magnetic field comes from relaxation phenomena and hysteresis loop^5,6,20,33^. Relaxation phenomena include Brownian motion of particles with liquid medium and single domain relaxation (10^−5^ – 10^−10^ s^−1^) and hysteresis loss arises during AC frequency (265 kHz) and applied magnetic field (see SI6). Relaxation phenomena are dominating factor in case of smaller particles sizes, whereas hysteresis loss is dominating factor in case of bigger particles. For comparison, heating behaviors of other particles coated with thioglycolic acid as well as aminophosphonate are shown in Fig. 4(a). Variation in heating is due to the different amounts of capping agents present over the surface of particles.

### Zeta-potential study

Zeta-potential possessed by the particles dispersed in water is measured at pH 5, 6, 7 and 8. The zeta-potential values of thioglycolic acid, aspartic acid and aminophosphonate functionalized particles (core: 7 nm) at pH = 5 are −28.1 ± 1, −5.1 ± 0.6 and −25.5 ± 0.1 mV, respectively. With increase of pH up to 8, the zeta-potential value of thioglycolic acid functionalized particles does not change, whereas that of aspartic acid or aminophosphonate functionalized particles increases. At pH = 8, the zeta-potential values of aspartic acid and aminophosphonate functionalized particles are −26.0 ± 0.5 and −38.8 ± 0.6 mV, respectively.

The zeta-potential measurements for the Dulbecco’s Modified Eagle Medium (DMEM) and the DMEM with serum are performed and found to be value of −8.1 ± 0.2 and −5.9 ± 0.5 mV, respectively. The zeta-potential measurements of the nanoparticles functionalized with thioglycolic acid, aspartic acid and aminophosphonate functional groups are also performed in these two media. The zeta-potential value of the thioglycolic acid functionalized particles in the DMEM is found to be −13.6 mV. But, its value in the DMEM containing serum is +2.5 mV. For MNPs functionalized with aspartic acid, corresponding zeta-potential values in DMEM and DMEM with serum are −11.5 and −0.03 mV, respectively. Zeta-potential values of MNPs functionalized with aminophosphonate in the DMEM and the DMEM with serum are −3.0 and −9.2 mV, respectively.

The MNPs are found to be stable in the cell-culture medium (DMEM) with serum for more than three hours. The stability charts of the MNPs aspartate dispersed in water and DMEM with serum are shown in the supporting information (see Figs S39 and S40). The possible reason for this stability is due to steric stabilization^34^. In general, the MNPs having positive charge on the surface are more prone to internalize the cell than MNPs having negative charge on the surface. However, there are reports of internalization of negatively charged nanoparticles also in literature^35^. The internalization of negatively charged nanoparticles is believed to occur through nonspecific binding and clustering of the particles on cationic sites on the plasma membrane (that are relatively scarcer than negatively charged domains) and their subsequent endocytosis. Our fluorescence imaging results showed significant uptake of MNP-aspartate-FITC in the MCF-7 cells. MNP-aspartate-FITC has a zeta potential of −0.03 mV (close to net zero charge) in presence of DMEM with serum (pH = 7.4), as compared to its zeta potential of −26 mV in distilled water at pH = 8. This significant reduction in negative zeta potential may be due to the interaction of these MNPs with the serum proteins. Probably, such reduced negative charge on MNPs may help in internalization of MNPs on the MCF-7 cells. MCF-7 cells have the zeta potential of −20 ± 0.4 mV^36^. However, the detailed mechanism of internalization needs to be further studied.

### Internalization study of magnetic nanoparticles in human breast adenocarcinoma cells

**Figure 5:**
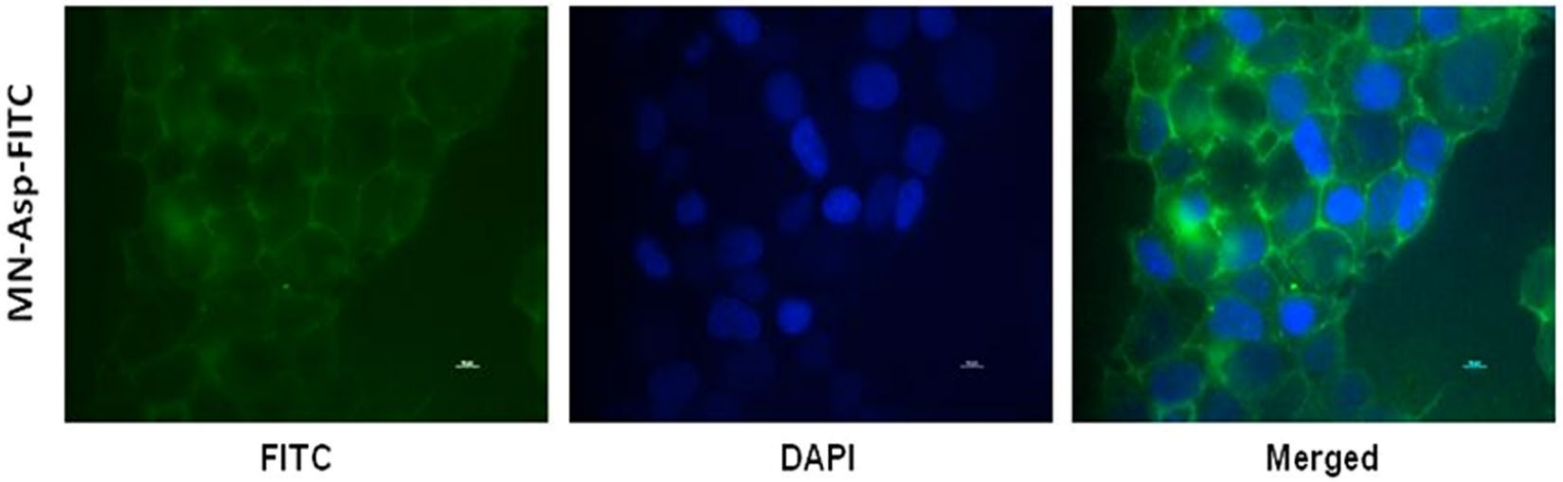
Fluorescence microscopy images of MCF-7 cells after treatment with MNP-aspartate-FITC. Nucleus is stained with DAPI (blue) and green color indicates MNPs labeled with FITC. Scale bar: 10 µm.

MCF-7 cells were treated with magnetic nanoparticles functionalized with different groups (viz., aspartic acid, thioglycolic acid and aminophosphonate). Using Prussian blue staining technique, blue coloured spots corresponding to presence of magnetic nanoparticles could be observed on the surface and inside the tumors cells, which again varied depending on nature of functionalized groups. These images indicate the internalization of magnetic nanoparticles in the tumor cells. It is interesting to observe that magnetic nanopaprticles functionalized with thioglycolate show faint staining in most of the tumor cells. Compared to this formulation, nanoparticles functionalized with aminophosphonate show more clustered localization in cell culture and the corresponding images are shown in Fig. S41. However, nanoparticles functionalized with aspartic acid show higher interaction and uptake in tumor cells resulting in darker blue spots. The differential staining and localization of nanoparticles functionalized with different groups may be associated with low Fe content or more homogenous distribution of MNPs in tumor cells. To further validate the higher intracellular uptake of MNP-aspartate in MCF-7 cells by Prussian blue studies, MNP-aspartate was labeled with a fluorescent dye (fluorescein iso-thiocyanate, FITC), followed by treatment of MCF-7 cells with 400 µl of MNP-aspartate-FITC. The cells were visualized by fluorescence microscopy. Green fluorescence is observed in the cytoplasm and near the surface of the cells. The nucleus stained with DAPI didn’t show co-localization with MNP-aspartate-FITC as observed in the merged image (Fig. 5) suggesting that the MNPs are localized mainly in the cytoplasm and cell surface.

### Cell cytotoxicity study of magnetic nanoparticles in human breast adenocarcinoma cells with or without AC magnetic field

**Figure 6:**
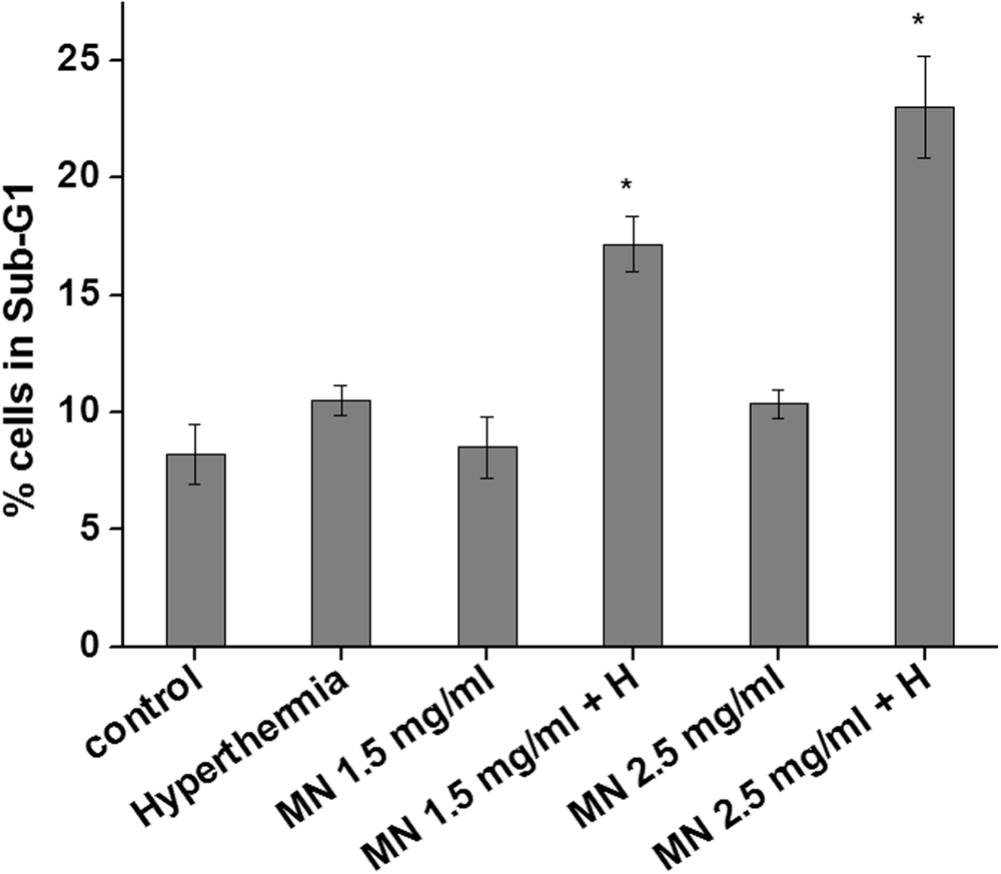
Sub-G1 analysis after PI staining by flow cytometry for MCF-7 cells after treatment with different concentrations of MNP-aspartate (1.5 and 2.5 mg/ml) in combination with or without *AC* magnetic field at 24 h. *Indicates that the values are significant at P < 0.05 as determined by Student’s t-test.

To determine the hyperthermia efficacy of MNP-aspartate in MCF-7 cells, they were treated with increasing concentrations of nano-formulation (1.5 and 2.5 mg/ml), followed by induction heating for 20 min and determination of cell cyto-toxicity by PI cell cycle analysis (Fig. 6). Results showed that as compared to only nanoparticles (8.5 ± 1.3% and 10.3 ± 0.6% cells in sub-G1 for 1.5 and 2.5 mg/ml MNP-aspartate treatments, respectively), MCF-7 cells treated with MNP-aspartate and hyperthermia therapy showed significant increase in % cells in sub-G1 phase of cell cycle (17.2 ± 1.2% and 23.02 ± 2.1% cells in sub-G1 for 1.5 mg/ml + hyperthermia and 2.5 mg/ml + hyperthermia treatments, respectively). The histogram analysis of flow cytometry is provided in Fig. S42. These results indicate that MN-aspartate in combination with *AC* magnetic field induces significant cell cyto-toxicity by apoptosis, suggesting its potential for cancer hyperthermia therapy applications.

## Experimental Details

### Materials required

All the materials were purchased from Sigma-Aldrich and used without further purification. All solvents were dried before use. Ferric chloride (99%), oleic acid (90%), 1 - octadecene (90%), thioglycolic acid (99%), 3 - chloroperbenzoic acid (70%), diethyl phosphite (98%), tertiary butyl amine (99%) and aspartic acid (99%) were used.

### Cell culture experiments

Human Breast Adenocarcinoma cell line (MCF-7) was obtained from National Centre for Cell Sciences, Pune, India. Cells were cultured in Dulbecco’s Modified Eagle Medium (DMEM; GIBCO, Invitrogen, Carlbad, CA, USA) supplemented with 10% fetal calf serum (FCS: Himedia Laboratories, Mumbai, India) and antibiotics (100 U ml^−1^ penicillin and 100 µg ml^−1^ streptomycin) in a humidified atmosphere of 5% CO_2_ at 37 °C. For studying the intracellular uptake, MNP-aspartate were labeled with FITC, followed by testing its internalization efficacy in MCF-7 cells (protocol for FITC labeling is given in SI7.8). Briefly, MCF-7 cells (1 × 10^6^) were seeded on glass coverslips for overnight at culture conditions, followed by treatment with MNP-aspartate-FITC for 3 h. The cells were then washed with PBS, followed by fixing in 4% paraformaldehyde for 20 min. at room temperature (RT). The cells were further washed with PBS and mounted on slide using Prolong Gold mounting media containing DAPI (Molecular Probes, USA). The cells were visualized by fluorescence microscopy under 40 X magnification.

For determining the hyperthermia efficacy of MNP-aspartate, 0.5 × 10^6^ cells were seeded in 35 mm Petri-dishes for overnight at culture conditions, followed by treatment with different concentrations of MNP-aspartate (viz., 1.5 and 2.5 mg/ml) for 3 h. Further, the cells were subjected to hyperthermia treatment for 20 min, followed by further incubation at culture conditions for 24 h. The cell viability was determined by PI cell cycle analysis by flow cytometry. Briefly, the cells were harvested by trypsinization followed by washing with PBS. The cell pellet was fixed with ice cold absolute methanol and stored at −20 °C till further use. For flow cytometry analysis, the cells were first permeabilized (0.1% Triton X-100 and 1 mg/ml sodium citrate in PBS), followed by staining with PI [containing freshly added RNAse solution (50 µg/tube)]. The flow cytometry (Partec, Germany) analysis was carried out at 488 nm excitation and 585 nm emission wavelengths. 20,000 cells were analyzed and % cells in sub-G1 phase of cell cycle were determined by using Cyflogic software.

### Synthesis of Fe_3_O_4_ particles

Reaction of ferric chloride with oleic acid at room temperature was used to prepare iron oleate. Iron oleate solution was slowly heated at the rate of 4 °C per minute to reach 320 °C in presence of 1- octadecene in inert atmosphere. Once the desired temperature was reached, it was maintained for 1 h. Change of brownish to black coloration was observed after about 30 minutes of heating suggesting formation of Fe_3_O_4_ particles. The particles were characterized by Fourier transform infrared (FT-IR) spectroscopy, thermo-gravimetric analysis (TGA) and differential thermal analysis (DTA). The detailed procedures for synthesis have been given in SI1. Thus, oleic acid coated Fe_3_O_4_ particles were prepared. These particles were found to be dispersible in chloroform and hexane etc., but not dispersible in water which can be ascribed to hydrophobic nature of the oleic acid capping. Amount of oleic acid present on the surface of the particles and iron were calculated theoretically (SI4) using the spherical and cuboid models of the particles. Different possible arrangements of COO^−^ groups of oleate molecules are also given in detail in SI4. From TGA data, we found oleic acid contributed a minimum of ~24% of the particle’s weight, thus functionalization reactions on the particles itself saved lots of reagent.

To make these particles water dispersible, we planned three different strategies. Here, methyl oleate was used as a model before trying it on oleic acid coated nanoparticles. As methyl oleate has no acidic functional group (-COOH), addition and cleavage reactions to the C=C of oleate can be carried out. Otherwise, this functional group will affect other mechanism and also, this model is almost the same with the particle system. The -COO^−^ group of oleic acid is used to cap Fe_3_O_4_ particles. Synthetic procedure for methyl oleate is given in SI7.1.

### Addition of thioglycolic acid to the double bond of methyl oleate

**Figure 7:**
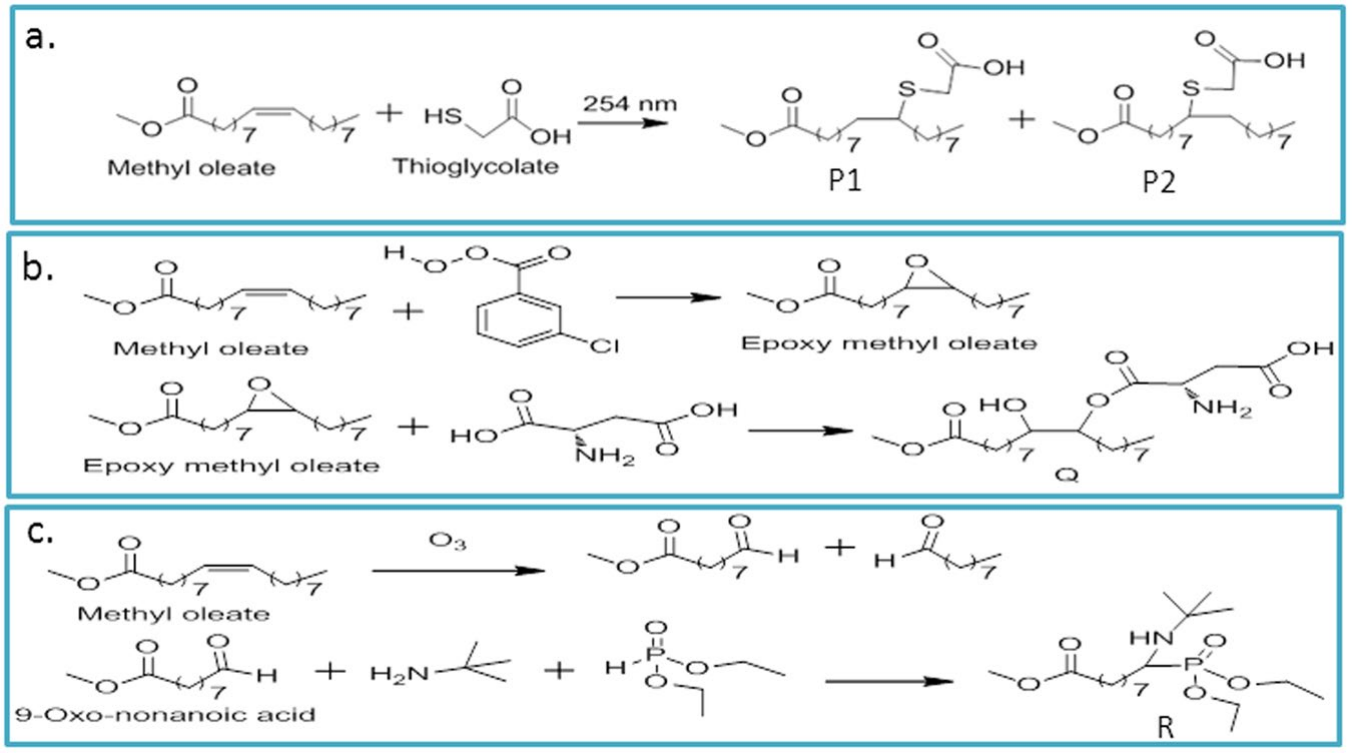
(**a**) Addition of thioglycolic acid to the double bond of methyl oleate. (**b**) Addition of aspartic acid to oxirane derivatives of methyl oleate and (**c**) Synthesis of aminophosphonate from aldehyde obtained from methyl oleate.

About 1 g of methyl oleate was dissolved in 10 mL of hexane and was mixed with 62 mg (2 equivalents) of thioglycolic acid in isopropanol. It was exposed to the ultraviolet lamp of 15 W having emission maxima at 254 nm. The reaction was monitored with the help of thin layer chromatography (TLC). The products (P1 & P2) were purified using column chromatography and characterized by nuclear magnetic resonance (NMR) and FT-IR spectroscopy (SI7.2A). The yield of the reaction was ~90% and the typical reaction scheme is shown in Fig. 7a.

We performed similar reaction on the nanoparticles in which methyl oleate was substituted with oleic acid coated Fe_3_O_4_ nanoparticles. The thioglycolic acid functionalized particles were separated by centrifugation and were washed with methanol. These particles were characterized by FT-IR spectroscopy (SI7.2B), and found to be water dispersible (Fig. 1iv-A).

### Addition of aspartic acid to oxirane derivatives of methyl oleate

About 20 mg of the methyl oleate was dissolved in 20 mL of dichloromethane and this solution was cooled to 0 °C. Dried 3-chloroperbenzoic acid in dichloromethane was added drop-wise to the cooled solution with stirring. The reaction mixture was kept for 3 h stirring at the room temperature. The product was purified and characterized by FT-IR and NMR spectroscopy (SI7.3A). The yield of the reaction was ~80%. The obtained oxirane and aspartic acid (2 equivalents) were dissolved in dimethyl formamide (DMF) and heated to 60 °C with the addition of catalytic amount of boron trifluoride. The reaction was monitored using TLC (thin layer Chromatography) and it was found to be complete in 4 h. The product (Q) was purified and characterized by FT-IR and NMR spectroscopy. The yield of the reaction was ~70% and the typical reaction scheme is shown in Fig. 7b.

Similar reaction was performed on the nanoparticles. The aspartic acid functionalized particles were washed with methanol to remove excess aspartic acid and separated by centrifugation. The particles were characterized by FT-IR spectroscopy (SI7.3B). These particles were found to be water dispersible (Fig. 1iv-B).

### Synthesis of aminophosphonate from aldehyde obtained from methyl oleate

About 10 mg of methyl oleate was dissolved in 30 mL of dichloromethane and cooled to −50 °C to prevent explosion due to molozonide that is formed by the reaction of ozone on C-C double bond of methyl oleate. Maintaining this temperature, ozone gas was passed. After passing for one and half hour, blue colouration of the reaction mixture was observed. It indicated excess of ozone gas present in the reaction medium. Triphenyl phosphine (3 equivalents) was added to the reaction mixture along with passing of oxygen gas. The reaction mixture was kept stirring for overnight at room temperature. The product was purified using column chromatography and characterized by FT-IR and NMR spectroscopy (SI7.4A.1). The yield of the reaction was ~90%.

Using the aldehyde having ester group only, α-aminophosphonate of tertiary butyl amine and diethyl phosphite was synthesized by Kabachnik - Fields reaction. The product (R) was purified and characterized by FT-IR and NMR spectroscopy (SI7.4A.2). Detailed procedure is given in the supporting information. The typical reaction scheme is shown in Fig. 7c.

Similar reaction was performed on the particles. The amino-phosphonate functionalised particles were washed with ethyl acetate and collected with centrifugation. The particles were characterized with FT-IR spectroscopy (SI7.4B) and found to be water dispersible (Fig. 1iv-C).

### Synthesis of bigger Fe_3_O_4_ particles

To study the heating effect of different sizes and shapes of Fe_3_O_4_ particles under AC magnetic field, bigger particles were prepared. A stock solution of 500 mL of ferric oleate in 1 - octadecene (0.16 g/mL) was prepared. About 1 mL of ferric oleate was added to the already synthesized particles that were synthesized starting from 10 mL of stock solution. Following the same procedure of particles synthesis, reaction mixture was heated for 1 h at 320 °C (heating rate of 4 °C per minute to reach 320 °C). In the second set, 5 mL of ferric oleate was added to the already synthesized particles and reaction mixture was heated for 1 h at 320 °C. The particles formed after addition of 1 and 5 mL of ferric oleate are designated as 1 mL and 5 mL, respectively. In the third set, 5 mL of ferric oleate was added to the already synthesized particles and reaction mixture was heated for 8 h at 320 °C. The size and morphology of the particles were investigated with TEM (Fig. 2D–F).

### Characterization

FT-IR spectra were recorded using Bomem spectrometer (Hartmann and Braun, MB – 100 series). NMR data were recorded on a Bruker 400 MHz spectrometer using tetramethylsilane or H_3_PO_4_ as the standard reference. X-ray diffraction (XRD) patterns were recorded using Rigaku Miniflex 600 machine and crystallite size (t) was calculated using the Scherrer equation t = (0.9 λ)/(B cosθ), where λ is the wavelength of Cu Kα, B the half width at maximum intensity and θ the Bragg’s angle. Thermogravimetric analysis (TGA) and differential thermal analysis (DTA) data were recorded with TG - DTA - EGA Setaram (Setsys evolution) in an argon atmosphere in 20–600 °C. Transmission electron microscopic images of the particles were taken with 200 keV JEOL (HRTEM) (SI1).

The magnetization and magnetic hysteresis measurements were performed using a Physical Property Measurement System (PPMS) (Model: 6000, Quantum Design) equipped with vibrating sample magnetometer (VSM) option and a superconducting magnet producing fields up to ±9 Tesla. To carry out this measurement, the samples were packed in a pocket (capsule) made from polytetrafluoroethylene (PTFE) tape. The mass of the sample was chosen in the range of 8–12 mg in order to obtain a good signal-to-noise ratio. For zero field cooled (ZFC) measurement, the samples were at first cooled in zero magnetic field down to 5 K and then magnetizations were recorded by increasing the temperature in an applied magnetic field (i. e. 50 Oe). For field cooled measurement on warming (FC or FCW), samples were cooled down to 5 K in the presence of magnetic field and then magnetizations were recorded by increasing temperature in the presence of the same field (i.e. 50Oe). To obtain the field dependence of the magnetization (i.e. hysteresis loop), the samples were brought to a specific temperature and then the sample’s magnetic moment, as a function of the magnetic field with field stabilization time 90 s, was recorded in the magnetic field range of ±9 Tesla.

Induction heating of MNPs (Fig. 4) was performed in plastic micro-centrifuge tube (1.5 ml) using AC magnetic field (Easy Heat 8310, Ambrell, UK) with 6 cm diameter (4 turns) coil. Particles (5–10 mg) suspended in 1 mL of distilled water were placed at the centre of coil and the applied frequency was 265 kHz. Duration of heating was 10 min. Calculated values of magnetic field (H) at currents 100, 200, 300, 400, 500 and 600A were 83.8, 167.6, 251.4, 335.2, 419 and 502.8Oe (equivalent to 6.7, 13.3, 20.0, 26.7, 33.3 and 40.0 kA/m), respectively. Specific absorption rate (SAR) per Fe_3_O_4_ of the sample was calculated following the same procedure in our previous paper (SI6)^6,21^. The zeta-potential measurements were done using Zetasizernanoseries, Malvern Instruments.

For FT-IR, TGA-DTA and XRD experiments, pristine Fe_3_O_4_ particles (prepared with a heating rate of 4 °C per minute to reach 320 °C) were used.

## Conclusions

Monodispersed Fe_3_O_4_ MNPs have been prepared from iron oleate. These particles have been made water dispersible by functionalization with different functional groups. Shape of particles was found to change from spherical to cuboid. The cuboid shape in turn enhances magneto-crystalline anisotropy (*K*_*u*_). Particle size can be increased by heating pristine particles in presence of ferric oleate solution for different periods of time. The increase in size of particle has been explained by Oswald ripening mechanism. The time required to reach hyperthermia temperature changes with shape and size of particles. The MNPs functionalized with aspartic acid showed better interaction or internalization in human breast cancer cells and showed enhanced cell killing under AC magnetic field. These results suggest potential of these nano-formulations for cancer hyperthermia therapy applications. However, it needs to be further studied in detail.

## Electronic supplementary material

Supplementary Information
