# Supplementary material for: Synthesis and characterization of monodispersed water dispersible Fe3O4 nanoparticles and in vitro studies on human breast carcinoma cell line under hyperthermia condition
Source: Sci Rep. 2018 Oct 3;8:14766. doi: 10.1038/s41598-018-32934-w (PMC6170485; doi:10.1038/s41598-018-32934-w)
Supplement: Supplementary file 1 — Supplementary Information [file 41598_2018_32934_MOESM1_ESM.pdf]

Supplementary Information

For

Synthesis and characterization of monodispersed water dispersible Fe_3O_4 nanoparticles and in vitro studies on human breast carcinoma cell line under hyperthermia condition

K. S. Sharma[1], R. S. Ningthoujam[1*], A. K. Dubey[2], A. Chattopadhyay[2], S. Phapale[1], R. R. Juluri[3], S. Mukherjee[4], R. Tewari[5], Neena G. Shetake[6], B. N. Pandey[6] and R. K. Vatsa[1]

1 Chemistry Division, Bhabha Atomic Research Centre, Mumbai-400085, India

2 Bio-organic Division, Bhabha Atomic Research Centre, Mumbai-400085, India

3 Institute of Physics, SachivalayaMarg, Bhubaneswar-751005, India

4 UGC-DAE Consortium for Scientific Research, Mumbai Centre-400085, India

5 Material Science Division, Bhabha Atomic Research Centre, Mumbai-400085, India

6 Radiation Biology and Health Sciences Division,Bhabha Atomic Research Centre, Mumbai-400085, India

***Corresponding author: rsn@barc.gov.in (RSN)**

Phone: +91 2225592321

Fax: +91 2225505151

SI 1 Synthesis of monodispersed Fe_3O_4 magnetic nanoparticles
Materials required: Ferric chloride, oleic acid, 1-octadecene, sodium hydroxide, sodium sulphate anhydrous, petroleum ether, and methanol.

SI 1.1 Synthesis of ferric oleate

Procedure: To a solution of ferric chloride (5 g, 18.5 mmol.) prepared in methanol (20 mL), petroleum ether (25 mL) containing oleic acid (18 mL, 56.5 mmol.) that was treated with sodium hydroxide (2.26 g in 10 mL water) was poured. After stirring for 1 h, organic layer was separated, refluxed at the temperature of 60 ^{o}C for 1 h and concentrated with rotavapour and high vacuum pump. A waxy solid obtained was characterized for ferric oleate by FT-IR spectrometer (see **Fig 1ii** of main manuscript or **Fig. S1**). Yield of the reaction was 95%.

By the same procedure, 80 g of ferric oleate was prepared and dispersed in a 500 mL of volumetric flask with 1-octadecene (0.16 g per mL). This would be used as the stock solution for all the synthesis.

FT-IR (cm^{-1}) of ferric oleate: 3435 (broad) (O-H stretching of water present), 3011 (m) (sp^2 C-H stretching vibration), 2956/2920/2850 (s) (sp^3 C-H stretching vibration), 1645 (m) (C=C stretching vibration), 1558 (s) (asymmetric –COO stretching), 1462 (m) (CH_2 bending vibration), 1446 (m) (symmetric $–COO^-$ stretching vibration), 1346 (m) (CH_3 bending vibration), 720 (w) (long chain band). (Ref. 1, 2)

FT-IR (neat, cm^{-1}) oleic acid: 3625-2746 (broad) (O-H stretching of carboxyl group), 3007 (m) (sp^2 C-H stretching vibration), 2929, 2855 (m) (sp^3 C-H stretching vibration), 1712 (s) (C=O stretching vibration), 1652 (m) (C=C stretching vibration), 1464 (m) (CH_2 bending vibration), 942 (s) (O-H loop), 723 (w) (long chain band). (Ref. 1)

Notes: s = strong, m = medium and w = weak

References:

1. Pavia, D. L., Lampman, G. M., Kriz, G. S., Introduction to spectroscopy, page 26, third edition (Thomson Learning, Washington-2001).

2. Ghosh, R., Pradhan, L., Devi, Y. P., Meena, S. S., Tewari, R., Kumar, A., Sharma, S., Gajbhiye, N. S., Vatsa, R. K., Pandey, B. N., Ningthoujam, R. S. Induction heating studies of Fe_3O_4 magnetic nanoparticles capped with oleic acid and polyethylene glycol for hyperthermia. J. Mat. Chem. **21**, 13388 – 13399 (2011).

Figure S1. FT-IR spectra of ferric oleate and oleic acid. Thick blue colored spectrum corresponds to ferric oleate and green thin to oleic acid

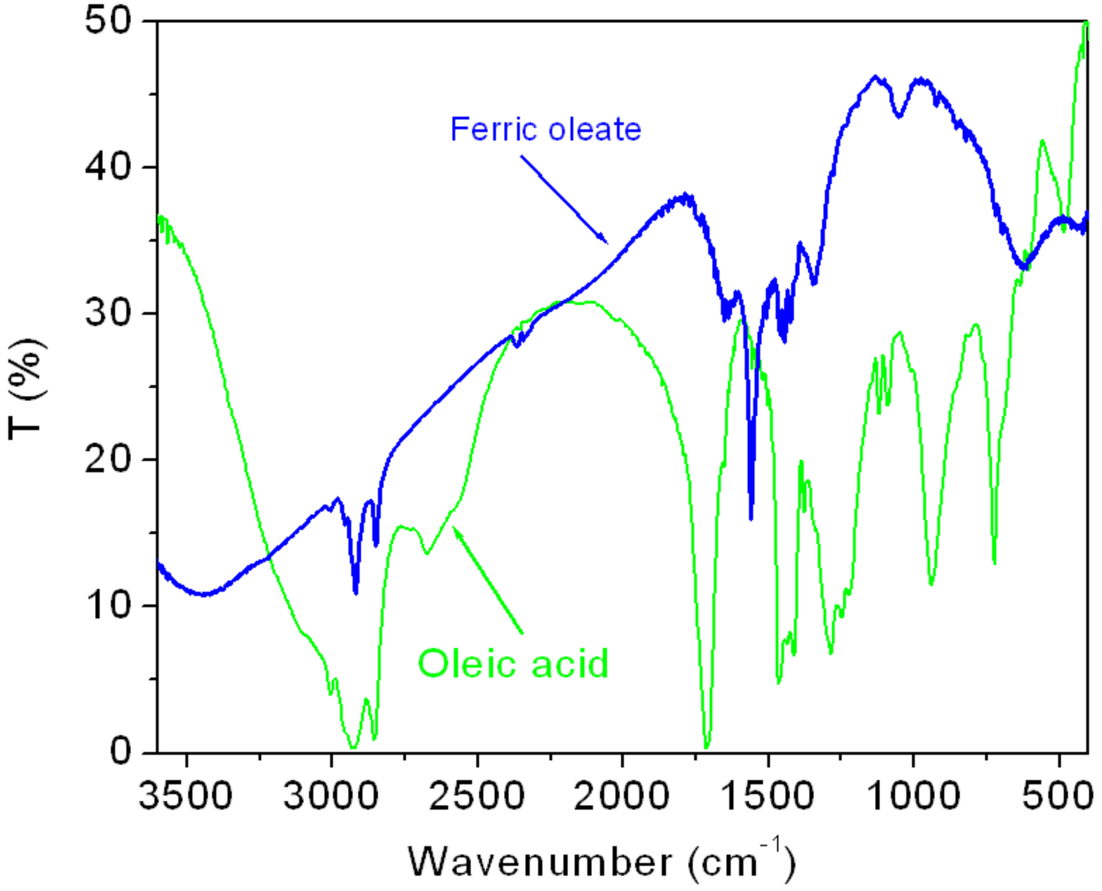

SI 1.2 Synthesis of Fe_3O_4 magnetic nanoparticles

Procedure: To a three necked flask, ferric oleate (10 mL, equivalent to 1.6 g ferric oleate), 1-octadecene (20 mL) and oleic acid (5 mL) were added. With blowing argon gas for 10 minutes before heating, the reaction mixture was heated at rate of 4 ^oC per minute to reach 320 ^oC and maintained for 1 hour. A change of colour from brownish to black was observed in the reaction mixture that confirmed the formation of Fe_3O_4 particles. By suddenly cooling to the room temperature the particles was precipitated with acetone and the excess oleic acid and 1-octadecene remained was removed by dispersion in petroleum ether, precipitation in acetone and decanting the supernatant with the help of magnet. This was carried out for three times. The obtained Fe_3O_4 particles were characterized with Fourier Transformed Infrared spectrometer (FT-IR) (see **Fig. S2** or **Fig. 1ii** of main manuscript), X-ray Diffraction (XRD) pattern (see **Fig. 1i** of main manuscript), Thermogravimetric analysis (TGA) (see **Fig. 1iii**) of main manuscript or **Fig. S3**), Superconducting Quantum Interference Device (SQUID) (see **Fig. 3A, 3F** of main manuscript) and Transmission Electron Microscopy (TEM) (see **Fig. 2A** of main manuscript or **Fig. S4**). For heating rate of 1.3 ^oC per minute to reach 320 ^oC, TEM image is shown (see **Fig. 2(G)** of main manuscript or **Fig. S5**)

Characterization of the particles

FT-IR (KBr, cm^{-1}): 3007 (m) (sp^2 C-H stretching vibration), 2957/2921/2852 (s) (sp^3 C-H stretching vibration), 1699 (s) (C=O stretching vibration of unbound/free oleic acid), 1560 (s) (COO^- asymmetric stretching vibrations), 1462 (m) (CH_2 bending vibrations), 1422 (m) (COO^- symmetric stretching vibrations), 720 (w) (long chain band vibration), 535 (w) (Fe-O vibration). (Ref. 1)

Correlation of XRD pattern: Planes correspond to JCPDF no. 82-1533 using Bragg's equation ($\lambda = 2d\sin\theta$, in which λ, d and θ are wavelength of x-ray, inter-planner space and Bragg's angle). This suggests cubic structure of Fe_3O_4 particles with cubic structure having lattice parameter of a = 8.363 Å.

2θ / degree	Plane corresponding from the XRD pattern		
	H	k	l
30.17	2	2	0
35.50	3	1	1
43.04	4	0	0
56.76	3	3	3
62.49	4	4	0

TGA data of the particles

Temperature interval (^{o}C)	Weight loss (%)	Characteristics (Assignment 3 & 4)
< 200	1	Water present
300 - 500	24	Decomposition of oleic acid
Total loss	25	Oleic acid plus water present

Notes: From literature, amount of oleic acid present as capping agent was reported 25 - 30% (Ref. 3). In case of our, amount of oleic acid was found to be about 24 %.

References:

3. (a) Limaye, M. V., Singh, S. B., Date, S. K., Kothari, D., Reddy, V. R., Gupta, A., Sathe, V., Choudary, R. J., & Kulkarani, S. K. J. Phys. Chem. B **113**, 9070 - 9076 (2009); (b) Roca, A. G., Morales, M. P., Grady, K. O., Serna, C. J. Structural and magnetic properties of high temperature decomposition of organic precursors. Nanotechnology 17, 2783 – 2788 (2006).

4. Jadhav, N. V., Prasad, A. I., Kumar, A., Mishra, R., Dhara, S., Babu,K. R., Prajapat, C. L., Misra, N. L., Ningthoujam, R. S., Pandey, B. N. & Vatsa, R. K. Synthesis of oleic acid functionalized Fe_3O_4 magnetic nanoparticles and studying their interaction with tumor cells for potential hyperthermia applications. Colloids and surfaces B: Biointerfaces **108**, 158 - 168 (2013).

Figure S2. FT-IR spectra of Fe_3O_4 particles and ferric oleate. Inset shows the spectral region from 3050 to 2800 cm^{-1}.

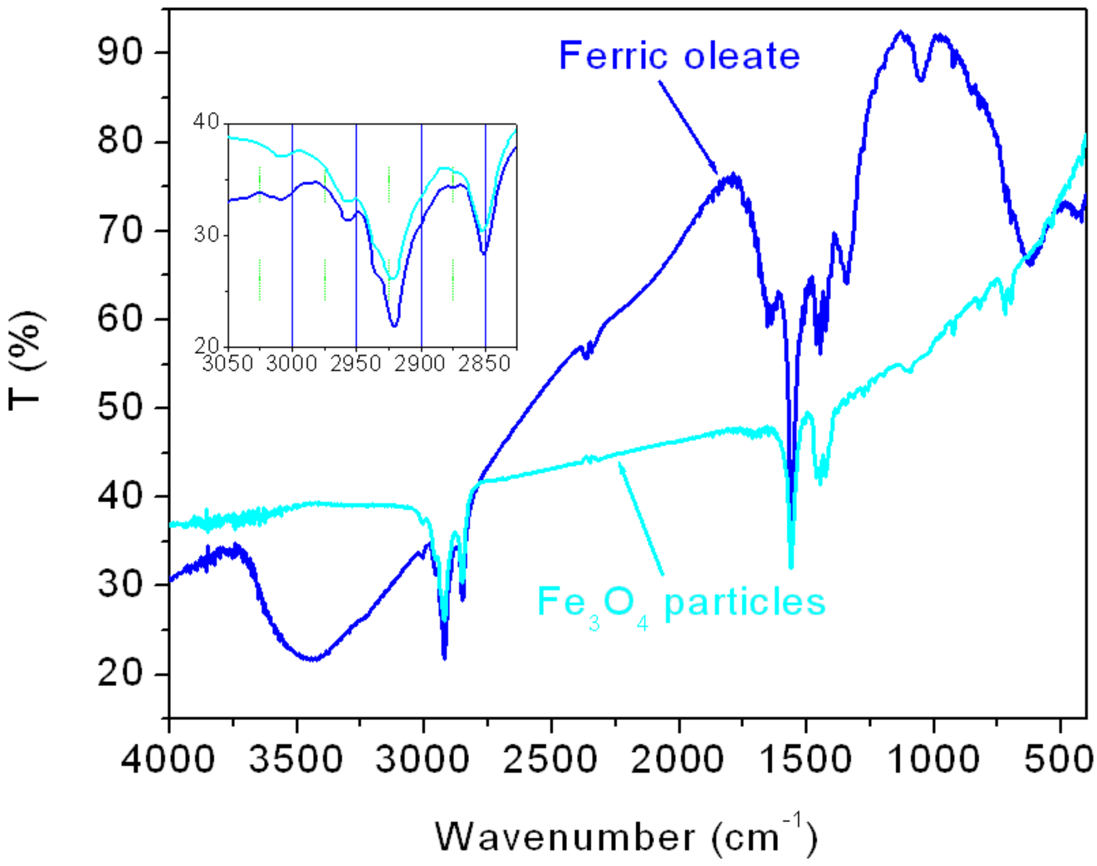

Figure S3. TGA and DTA curves of Fe_3O_4 nanoparticles in Ar atmosphere

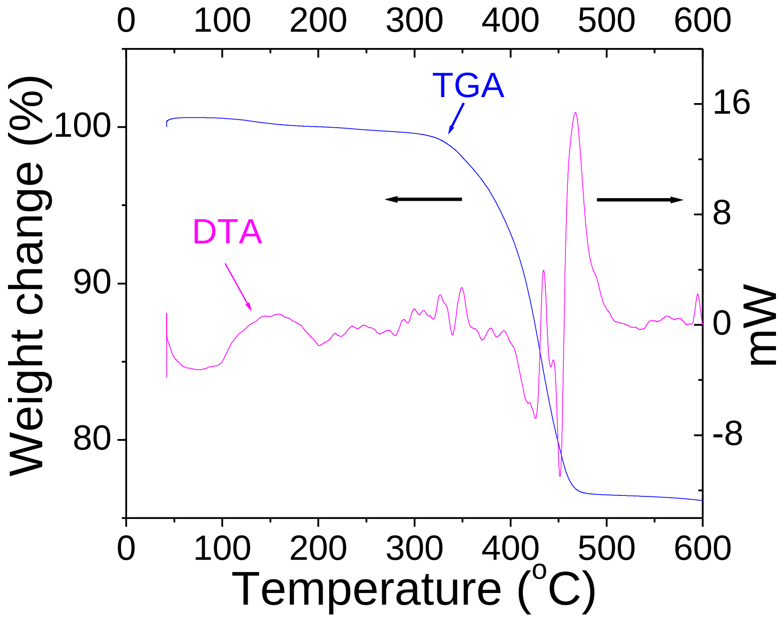

Figure S4. TEM image of Fe_3O_4 NPs

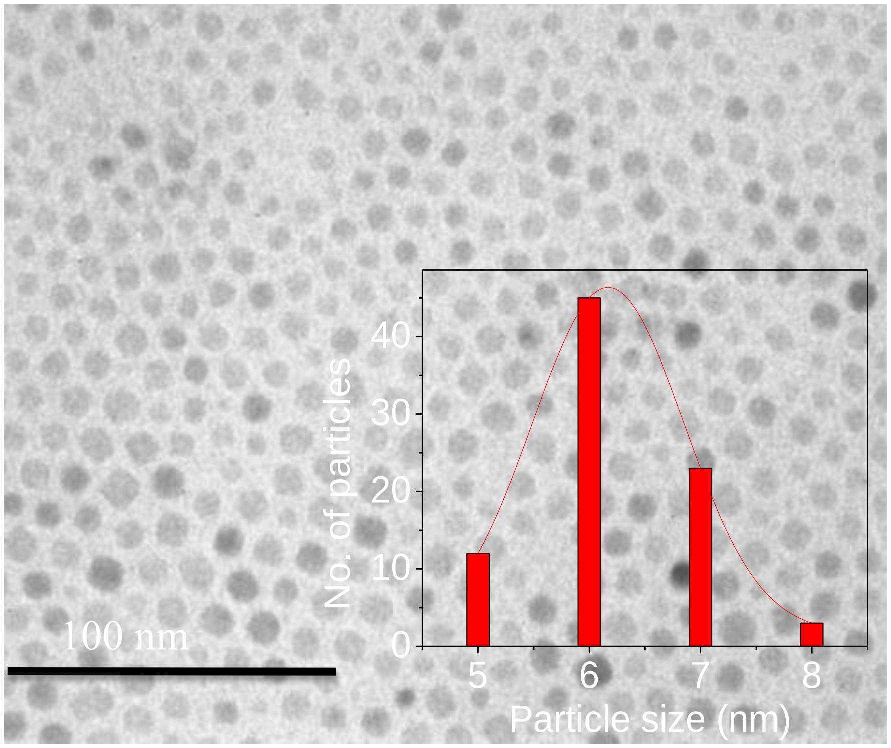

Figure S5. TEM image of Fe_3O_4 NPs

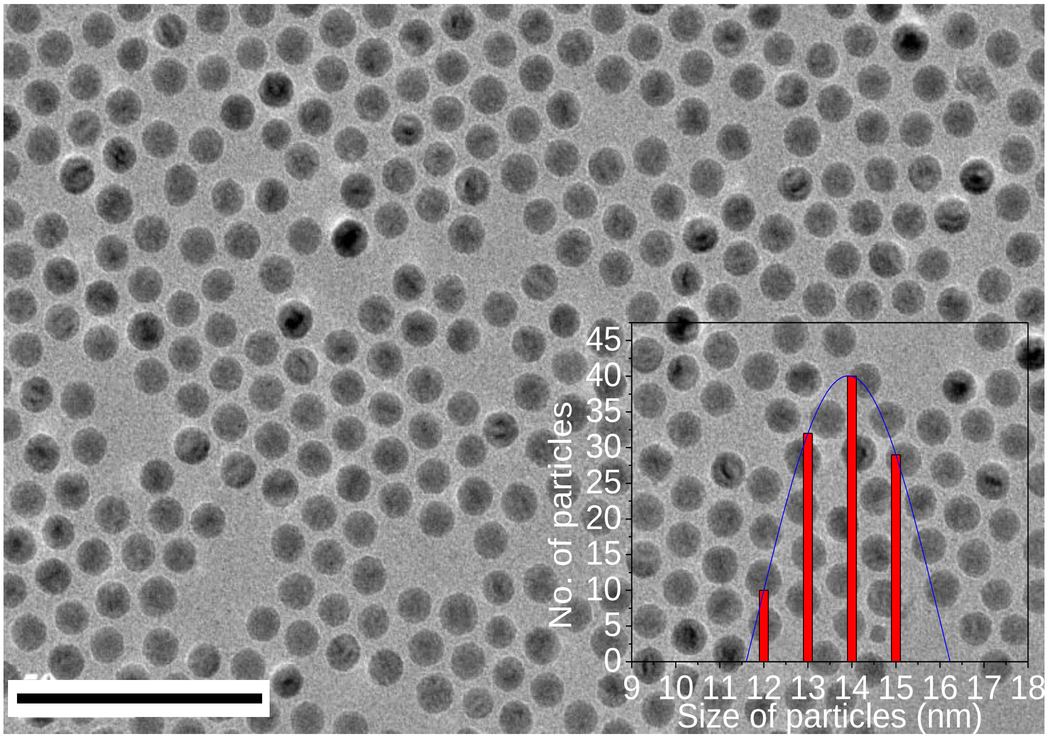

Scale bar is 100 nm

SI 2 Synthesis of magnetic nanoparticles by core – shell model

Using 10 mL of the prepared ferric oleate solution and 20 mL of 1-Octadecene and 5 mL of oleic acid, Fe_3O_4 particles were synthesized. After the synthesis of Fe_3O_4 particles and cooling to the room temperature, about 1 or 5 mL of ferric oleate was added and following the same process of the particles synthesis **(SI 1.2)** (heating rate of 4°C per minute), it was heated and kept in the reaction temperature for 1 or 8 h. Morphology and magnetic properties of the obtained particles were studied with TEM (**Fig. 2D, 2E, 2F**) of the main manuscript) and SQUID (**Fig. 3B, 3C, 3E, 3G, 3H and 3I** of the main manuscript) respectively.

SI 3 Importance of three protocols to make water dispersible Fe_3O_4 particles

There are not many reports regarding addition or cleavage reactions of the double bond of oleic acid for making particles dispersible in water. Addition or cleavage reactions can help in functionalization with one or more groups so that it can be used for dispersion, conjugation with antigens and fluorescence.

The first protocol (thioglycolic acid coated Fe_3O_4 MNPs) is very simple and can be used for wide range of hydrophobic monodispersed particles as the reaction time is short and does not involve use of costly reagents. This type of thiol-ene click reaction of the double bond of oleic acid can be used for making thiol dendrimers around the magnetic particles so that magnetic and luminescent nanohybrids can be prepared (Ref. 5, 6).

The second protocol uses aspartic acid to open the epoxide generated from oleic acid. As the particles contain amino acid groups as well as the long hydrocarbon chain of oleic acid, it can behave hydrophobic as well as hydrophilic and will help the particles to sweep faster in biological fluid and cell membrane. Additionally, many different amino acids or peptides (i.e. iRGD) can be used for further functionalization of the magnetic nanoparticles. This secondary functionalization can be utilized for targeting the cancer cells.

The third protocol converts capping agent oleic acid into highly polar α-amino-phosphonate functional group. Due to the presence of both amino and phosphonate groups, the binding ability of these nanoparticles for divalent and trivalent metal ions will be high. Particles functionalized with amino-phosphonate groups can be used for targeting group, hyperthermia treatment and removal of toxic metal ions without further addition of specific targeting groups to the particles (Ref. 7-11). In addition, these particles can act as inhibitor of many enzymatic reactions and targeting agents (Ref. 8, 11).

References:

5. Dondoni, A. The emergence of thiol–ene coupling as a click Process for materials and bioorganic chemistry. *Angew. Chem. Int. Ed.* **47**, 8995 – 8997 (2008).

6. Killops, K. L., Campos, L. M., & Hawker, C. J. Robust, efficient, and orthogonal synthesis of dendrimers via thiol-ene “click” chemistry. *J. Am. Chem. Soc.* **130**, 5062 – 5064 (2008).

7. Galezowskaa, J. & Kontecka, E. G. Phosphonates, their complexes and bio-applications: A spectrum of surprising Diversity. *Coord. Chem. Rev.***256,** 105 – 124 (2012).

8. Naydenova, E. D., Todorov, P. T. & Troev, K. D. Recent synthesis of aminophosphonic acids as potential biological importance. *Amino Acids* **38**, 23 – 30 (2010).

9. Fernandez, Y., Maranon, E., Castrillon, L. & Vazquez, I. Removal of Cd and Zn from inorganic industrial waste leachate by ion exchange. *J. Hazard. Mater. B.* **126,**169 – 175 (2005).

10. Kiefer, R. & Holl, W. H. Sorption of heavy metals onto selective ion-exchange resins with aminophosphonate functional Groups. *Ind. Eng. Chem. Res.***40**,4570 - 4576 (2001).

11. Kafarski P. & Lejcxak B. Biological activity of aminophosphoric acids. Phosphorus, Sulfer and Silicon **63**, 193-215 (1991).

SI 4 Theoretical calculation of oleic acid contribution to the weight of the particle

SI 4.1 Calculation of iron ions on the surface

Here, we consider hard-sphere model of ions. Values of ionic radii are taken from the literature (Ref. 12). Fe^{2+}/ Fe^{3+} have high spin state (H. S.). In a molecule of Fe_3O_4, one Fe^{2+} and one Fe^{3+} are in octahedral sites (six coordination number, 6 C.N.) whereas one Fe^{3+} is in tetrahedral site (4 coordination number, 4 C.N.) in inverse spinel structure.

Ionic radius of Fe (II) (C.N. = 6, H. S.) = 0.77 Å

Ionic radius of Fe (III) (C.N. = 4, H. S.) = 0.49 Å

Ionic radius of Fe (III) (C.N. = 6, H. S.) = 0.645 Å

Ionic radius of O^{2-} (C.N. = 4) = 1.38 Å

Ionic radius of O^{2-} (C.N. = 6) = 1.4 Å

van der Waal's radius of oxygen is 1.5 Å

$$\text{Average ionic radius of Fe ions} = \frac{0.645+0.49+0.77}{3}$$
$$= 0.635\ \text{Å}$$

$$\text{Average ionic radius of } O^{2-} \text{ ions} = \frac{1.38+1.4\times2}{3}$$
$$= 1.39\ \text{Å}$$

Reference:

12. Shannon, R. D. & Prewitt, C. T., Effective ionic radii in oxides and fluorides. Acta Crystal. B **25**, 925 - 929 (1969).

Figure S6. Arrangement of Fe^{2+}/Fe^{3+} and O^{2-} ions on the surface of a particle

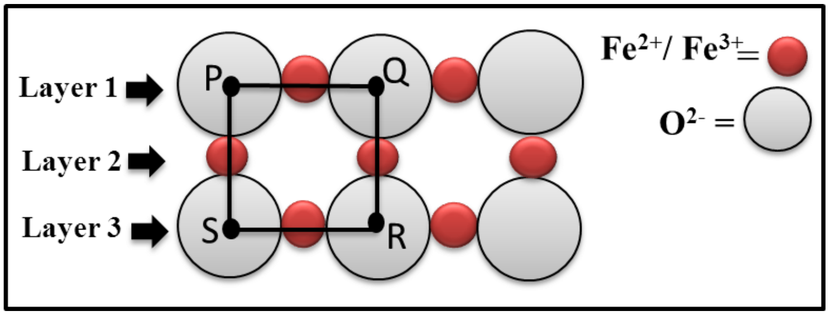

Arrangement of Fe^{2+} / Fe^{3+} and O^{2-} is shown in **Fig. S6**.

Let us consider the square PQRS,

Distance between P and Q ($\overline{PQ}$) = 2 × 0.139 + 2 × 0.064 = 0.406 nm

Area of the square (PQRS) = 0.406 nm × 0.406 nm = 0.1648 nm^2

So, the total number of these squares present on the surface of a particle (spherical or cuboid) is obtained by dividing the surface area of the particle by the area PQRS.

$$N_T = \frac{\text{Surface area of a particle}}{\text{Area PQRS}}$$

Each square contains four iron ions. But they are shared with another square.

So, Number of iron ions per square = 2

And, total number of iron ions on the surface of a particle = 2 × N_T

SI 4.2 How oleate ions occupy the surface of a particle

SI 4.2.1 Oleate ion (R-COO$^-$) occupying Fe^{2+}/Fe^{3+} ions in a plane

Figure S7. O---O′ distance in an oleate ion

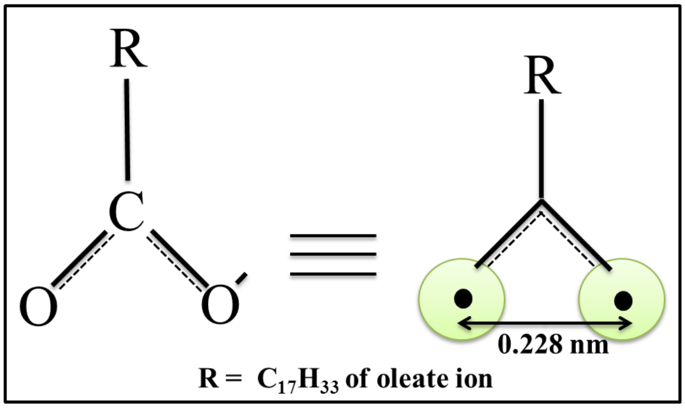

Oleate ion has functional group –COO$^-$ with hydrocarbon moiety (R = $C_{17}H_{33}$). This is shown schematically (**Fig. S7**). The distance between O----O′ is 0.228 nm (Ref. 13). R-COO$^-$ (oleate) ion can occupy surface of a particle in the following possible ways. R-COO$^-$ (oleate) ion can occupy XZ or YZ plane.

Let us consider Fe^{2+}/Fe^{3+} and O^{2-} ion in XY-plane in 2D (**Fig. S8**). R-COO$^-$ molecular ion is bonding to Fe^{3+}/Fe^{2+} ion. The horizontal plane containing R-COO$^-$ arranges along XZ plane or layer 1. Along layer 1, Fe---O---Fe or O---Fe---O bond distance is 0.406 nm. The distance of O---Fe---O---Fe---O is 0.812 nm. Within this distance whether two R-COO$^-$ molecules ions attaches to two Fe^{2+}/ Fe^{3+} ions or not. We know O---O in R-COO$^-$ is 0.228 nm. For minimum distance between two R-COO- along layer 1 on XZ plane will be 2 × 0.228 + 2 × 0.138 (actually, the value is less than 0.138 nm) = 0.722

Thus, difference between 2 × (O---Fe---O) and 2 (R-COO-) = 0.812 – 0.722 = 0.09 nm

= 0.9 Å

It means that R-COO$^-$ ions can occupy layer 1 in adjacent Fe^{2+}/ Fe^{3+} ions.

Reference:

13. Fabbrizzi, L., Leone, A., & Tagleitti, A. A chemo - sensing ensemble for selective carbonate detection in water based on metal – ligand interaction. Angew. Chem. Int. Ed. 40, 3066 (2001).

Figure S8. Oleate ions occupying Fe^{2+}/Fe^{3+}

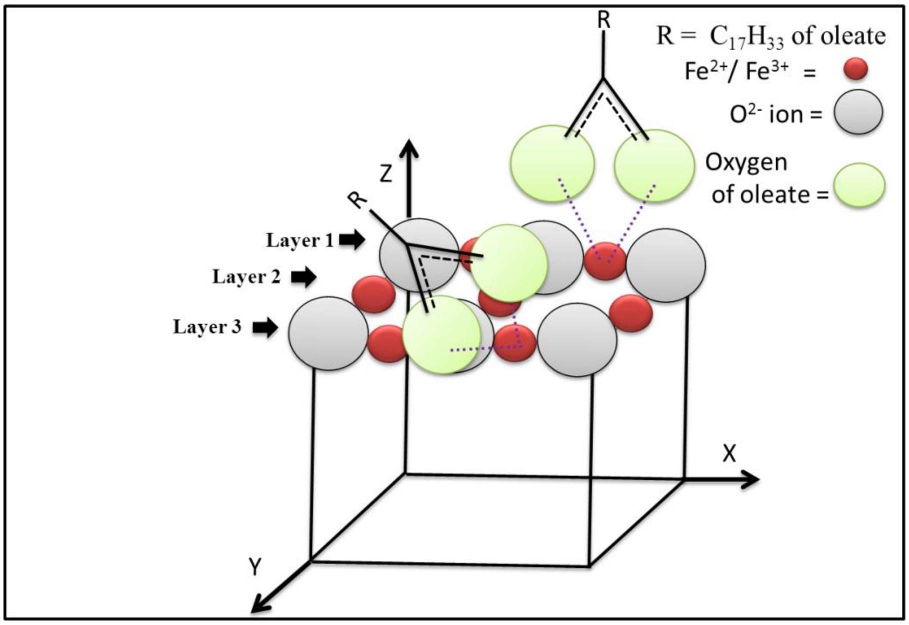

If we add R-COO$^-$ ions along layer 2 in XZ plane, whether is it feasible or not. Distance between layer 1 and layer 2 is same as Fe-O (i.e. 0.203 nm). The schematic diagrams are shown in **Fig. S8, S9**. The calculation is given below:

$\overline{PQ}$ = 0.406 nm

$\overline{LW}$ = 0.114 nm

$\overline{MW}$ = 0.203 – 0.114 = 0.089 nm

$\overline{LM}$ = 0.114 – 0.089 = 0.025

Then, $\overline{LN}$ = $\sqrt{0.0252^2 + 0.2032^2}$

= 0.204 nm

The O--O′ distance in two R-COO$^-$ ions is 0.204 nm, which is less than 0.276 nm (2 × 0.138 = 0.276 nm, a half of O$^-$ ion from R-COO in layer 1 and another half from R-COO in layer

2). There is not possible to occupy two R-COO$^-$ ions in layers 1 and 2 within square of 0.205 nm length. It is ruled out for possibility.

Figure S9. Arrangement of oleate ions in layer 1 and layer 2

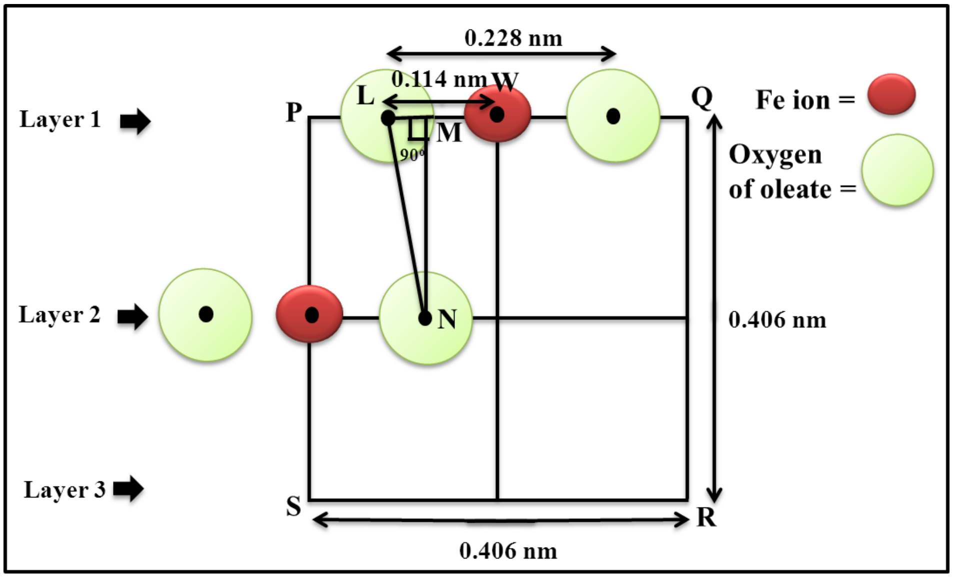

If we add R-COO- ion along layer 3 in XZ plane, R-COO$^-$ ion can occupy because distance between two layer (layer 1 and layer 3) is same as O---Fe---O (i.e. 0.405 nm) which is more than one O^{2-} ion (2 × 0.138 = 0.276 nm) i.e. a half is contributed by R-COO$^-$ ion and another is contributed by another R-COO$^-$ assuming O^{2-} has same size with R-COO$^-$. But in reality O^{2-} is much bigger than Oxygen of R-COO$^-$. Thus, two R-COO- ions can occupy 2 Fe^{2+}/ Fe^{3+} ions, which have minimum distance of 0.405 nm. Similarly, R-COO$^-$ ions can be arranged in YZ plane.

SI 4.2.2 R-COO- ions occupying Fe^{2+}/ Fe^{3+} diagonally

Let us consider R-COO- ions occupying Fe^{2+}/ Fe^{3+} ions diagonally. This is shown in **Fig. S10**. Fe-O (AB) is 0.203 nm. The distance between Fe—Fe (AC) is 0.286 nm. Along diagonal Fe---Fe---Fe will be 0.573 nm. The minimum distance between two R-COO- is 0.722 nm. Thus two R-COO$^-$ could not occupy Fe---Fe---Fe diagonally.

Figure S10. Three diagonally placed Fe^{2+}/Fe^{3+} ions

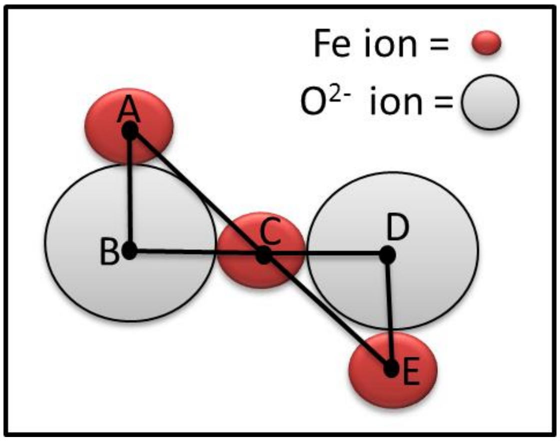

SI 4.2.3 One R-COO$^-$ occupies XZ - plane and adjacent R-COO$^-$ occupies YZ plane or vice-versa

One R-COO$^-$ occupies XZ-plane and another on YZ plane (**Fig. S11**). It is possibly only when 2 Fe^{3+}/Fe^{2+} ions have minimum distance of 0.406 nm. The distance between neighboring oxygen ions of two oleate ions can be calculated as shown below.

Figure S11. Arrangement of oleate ions on layer 1 and layer 3

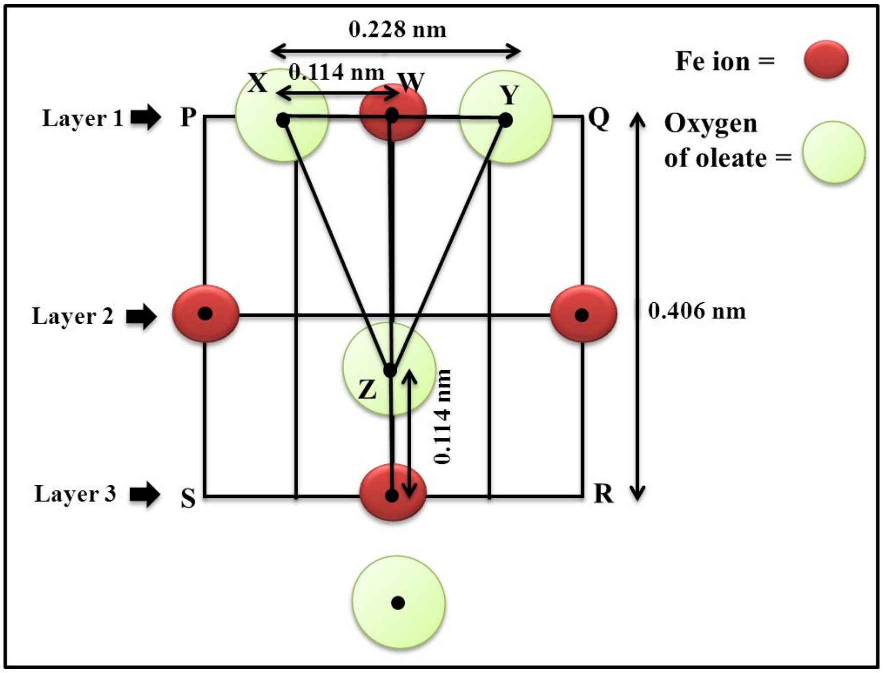

$\overline{WZ}$ = 0.406 nm – 0.114 nm = 0.292 nm

$\overline{XW}$ = 0.228/2 = 0.114 nm

$$\overline{XZ} = \sqrt{0.292^2 + 0.114^2}$$

= 0.313 nm which is more than 0.276 nm. It is feasible.

SI 4.2.4 Amount of oleic acid present

We can conclude that whatever way R-COO$^-$ ions occupy Fe^{2+}/Fe^{3+} ions, only one iron ion out of two in a square is capped by R-COO$^-$ (oleate) ion.

So, 2 iron ions ≡ 1 oleate ion

SI 4.2.5 Calculation of weight of a particle

Weight of a particle = volume of the particle × density of Fe_3O_4

SI 4.2.6 Calculation of the contribution of the weight of oleate ion on a particle of 6 nm size

SI 4.2.6.1 Considering the particle has spherical shape

For the spherical particle we know that

Radius of the particle = 3 nm

Then, surface area of the particle = 113 nm^2 (i.e. $4\pi r^2$, r = 3)

N_T = 686 number of squares

Total number of Fe ions on the surface = 2 × 686 = 1371 iron ions

1371 Fe ions ≡ 686 oleate ions

Avogadro's number ≡ 282.46 (molecular weight of oleic acid) – 1 (from H) = 281.46 g

So, 686 oleate ions ≡ 3.2×10^{-19} g

Calculation of mass of a particle

Density of Fe_3O_4 = 5×10^{-21} g nm^{-3}

Mass of the particle = volume of the particle × density of the particle

$$= (\frac{4}{3}\pi r^3) \times 5 \times 10^{-21} \text{ g nm}^{-3}$$

$$= 5.652 \times 10^{-19} \text{ g}$$

Percentage of weight

Contribution in weight by oleate ion is given by

$$= \frac{3.2 \times 10^{-19}}{5.65 \times 10^{-19} + 3.2 \times 10^{-19}} \text{ X } 100$$

= 36 %

SI 4.2.6.2 Considering the particles having cuboid shape

Length of a cuboid shaped particle = 6 nm

Surface area of the cube = $6L^2$ (L = side of a cube)

Surface area of the cube = 216 nm^2

Following the same assumption,

Number of squares on the surface = 216 / 0.1648 = 1211 squares

Number of Fe ions on the surface = 2 × 1211 = 2621 Fe ions

But, 2 Fe ions ≡ 1 oleate ion

2621 Fe ions ≡ 1311 oleate ions

So, weight of oleate molecules on the surface of the particles = 6.13×10^{-19} g

Volume of a cuboid particle = $L^3 = 6^3 = 216$ nm^3

Mass of a cuboid particle = $216 \times 5 \times 10^{-21}$ g = 10.8×10^{-19} g

Percentage of oleate ion weight to the weight of a particle = 36.2 %

Table S2. Percentage contribution of oleate ions to the weight of a particle

Size (nm)	No. of square unit on the surface (N_T)		No. of Fe ions per particle		No. of oleate ions per particle		Percentage contribution of oleate into the weight of the sample (%)	
	Cuboid	Spherical	Cuboid	Spherical	Cuboid	Spherical	Cuboid	Spherical
6	1211	686	2621	1371	1211	686	36.2	36
7	1784	934	3568	1867	1784	934	32.7	32
8	2330	1219	4660	2439	2330	1219	29.8	29.8
9	2949	1543	5898	3087	2949	1543	27.43	27.43
10	3641	1905	7282	3811	3641	1905	25.4	25.4
11	4405	2305	8811	4611	4405	2305	23.63	23.63
12	5243	2743	10485	5487	5243	2743	22.09	22.1
13	6153	3220	12306	6440	6153	3220	20.48	20.7
14	7136	3735	14271	7469	7136	3735	19.55	19.56
15	8192	4287	16383	8574	8192	4287	18.5	18.5

SI 5 Magnetic parameters

Table S1. Magnetic data of the particles are summarized below:

S. N.	Sample	Temperature (K)	H_C	M_R	M_S	Blocking temperature T_B
1	Core Fe_3O_4 particles	5	375	3.81	11.96	181
		310	26	0.1	11.01	
2	Fe_3O_4 particles shell from 1 mL of ferric oleate 1 h heating	5	910	5.0	19.27	188
		310	80	3.52	17.84	
3	Fe_3O_4 particles shell from 5 mL ferric oleate 1 h heating.	5	386	5.96	15.77	211
		310	37	0.12	11.96	
4	Fe_3O_4 particles shell from 5 mL ferric oleate 8 h heating.	5	688	20	90.0	180<br>282
		310	0	0	87.0	
5	Reported data on Fe_3O_4 particle in terms of size (nm) (ref. 14)					
Size = 4 nm		5	294	10.1	56.1	
		300	12	0	31.8	
11.5 nm		5	202	12.7	77.5	
		310	34	3.9	60.1	
47.7 nm		5	211	16.3	77.8	
		310	156	16.4	65.4	
150 nm		5	736	32.1	88.5	
		310	326	18.9	75.6	
Bulk (ref. 15)		M_S = 90 emu/g at 300 K and 92 emu/g at 5 K.				

References:

14. Goya, G. F., Berquo, T. S., Fonseca, F. C. & Morales, M. P. Static and dynamic magnetic properties of spherical magnetite nanoparticles. J. Appl. Phys. **94**, 3520 – 3528 (2003).

15. B. D. Cullity, Introduction to Magnetic Materials, London, Addison - Wesley Publishing Company, 1972.

SI 6 The reason behind heat generation by Fe_3O_4 magnetic nanoparticles in liquid medium under AC magnetic field

Magnetic nanoparticles dispersed in a fluid medium are found to generate heat under an applied AC magnetic field (ACMF). This heat generation can be explained by the following phenomena: (1) Brownian particles rotation after collision of the particles in the fluid medium, (2) Néel's spin relaxation, (3) hysteresis loss, and (4) eddy current. The first and second phenomena give major contributions to the heat generation. The contribution of the third phenomenon is less but that of the fourth one is almost negligible.

Figure S12. The Brownian rotation of two particles in a fluid medium

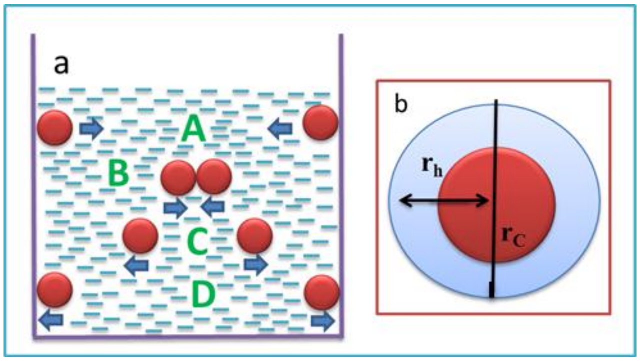

Brownian rotation can be explained schematically in **Fig. S12(a)**. In mode A, the particles are approaching after striking the wall of the container or themselves; mode B shows the collision between particles. In mode C, the particles rebound after collision and relax. The relaxation produces heat to the surrounding medium. These particles rotate to the equilibrium position before colliding with each other. Last mode D shows particles approaching towards the wall to collide. This whole process is known as Brownian relaxation (τ_B), which continues indefinitely. As the collision is the inelastic one, the loss of energy is compensated as heat to the medium. But, the mode of collision is different if MNPs are kept under the influence of the magnetic field like ACMF.

This relaxation (τ_B) at a particular temperature (T) is given by

$$\tau_B = \frac{4\pi\eta {r_h}^3}{k_B T}$$

Where η and r_h are respectively the dynamic viscosity of the carrier fluid and the hydrodynamic radius (sum of the radius of the particle and surfactant coating layer shown in **Fig. S12(b)**). k_B is the Boltzmann's constant. The τ_B becomes slow with the increase of r_h and η at a particular temperature.

Figure S13. The Néel's relaxation of particles

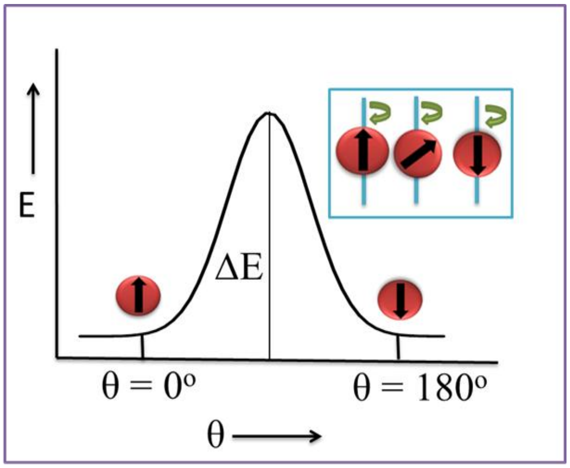

Generally, superparamagnetic particle is taken as a single domain. Each domain having a spin (↑) that orients from one direction to the opposite direction with relaxation (τ_N) of 10^{-9} s and without interacting each other. This is called as Néel's relaxation. But, the resultant net moment is zero. In **Fig. S13 (inest)**, the net moment is zero. This τ_N is given by

$$\tau_N = \tau_0 e^{\Delta E/k_B t}$$
$$= \tau_0 e^{KV/k_B t}$$

Where τ_0 is the order of 10^{-9} s and ΔE is the anisotropic energy barrier. ΔE equals to the product of the anisotropy energy constant (K) and the volume (V) of the particle. During the relaxation of spin, the excess energy is released to the surrounding medium as heat. τ_N becomes slow with the increase of K and V at a particular temperature. Thermal energy (k_BT) dominates over ΔE at smaller values of K and V. In such a situation, the spin of the domain rotates from 0° to 180° (**Fig. S13**).

In a DC magnetic field, the measured time is about 10 s that is too long to measure spin relaxation (10^{-9} s). Thus, the average moment is zero in a superparamagnetic regime. In an AC magnetic field of applied frequency (*f*) of 280 kHz which corresponds to 3.5×10^{-6} s, the direction of the current changes periodically within 4×10^{-6} s. Within this time window of 3.5 ×

10^{-6} s, the relaxation of spin of the domain could not be complete. So, the particles have net magnetic moment and hence hysteresis loss that is essential parameter for heat generation in an AC magnetic field is observed. The change of current over time can be plotted in the form of a loop. The area of the loop gives the hysteresis loss and is given by

$$A = f \int \mathrm{M\,dH}$$

Where, M is magnetization at the applied magnetic field (i.e., current is proportional to H)
Heat / power dissipation (P) of the whole system is given by

$$\mathrm{P} = \mu_0 \pi \chi'' \mathrm{f}\, \mathrm{H_0}^2$$

Where, μ_o is the permeability of free space. χ'' is the imaginary part of susceptibility (χ). Susceptibility is the magnetization divided by the applied magnetic field (χ = M/H). χ has two parts: real (χ') and imaginary (χ'').

$$\chi = \sqrt{\chi'^2} + \sqrt{\chi''^2}$$

The (χ'') is related to the heat dissipation of the system, which is defined as

$$\chi'' = \frac{\omega\tau}{1+(\omega\tau)^2}\chi$$

Here, τ is the total relaxation contributed by Brownian (τ_B) and Néel's (τ_N)

$$\frac{1}{\tau} = \frac{1}{\tau_B} + \frac{1}{\tau_N}$$

The fourth possibility of the heat generation is from eddy current (ED), which is an induced current and generally observed in the metallic rod. The ED is defined as

$$ED = \frac{(\mu\pi d f H_0)^2}{20\rho}$$

Where μ is the permeability of a material, d is the diameter of the particle, and ρ is the resistivity of the material. Since particles are so tiny and are suspended in the fluid medium, they cancel each other even if they possess eddy current (Ref. 16). Also, these MNPs behave as insulator with high value of ρ. Thus, the overall ED is negligible in the case of MNPs.

The density of the particle relates to the loss power density P or specific absorption rate (SAR), which can be calculated using following relation

$$SAR = C\frac{\Delta T}{\Delta t}\,\frac{1}{m_{magn}}$$

Where, C is the specific heat capacity of the sample. Usually, it is calculated by considering both the sample weight and the weight of the water. Comparing to the weight of the water, sample

weight is negligible, so only the specific heat capacity of water is contributed, and its value is 4.18 J g^{-1} K^{-1}. $\Delta T/\Delta t$ is the slope of the time-dependent versus temperature curve. The m_{magn} is the amount of magnetite or Fe in the 1 mL system.

Reference:

16. Ningthoujam, R. S., Vatsa, R. K., Kumar, A. & Pandey, B. N., "Functionalised magnetic nanoparticles: concepts, synthesis and application in cancer hyperthermia" Edited Banerjee, S. & Tyagi, A. K. Elsevier Inc., USA. Chapter 6, 229 – 260 (2012).

SI 7. Functionalization of particles to make dispersible in water

Notes: *For every functionalization reaction of Fe_3O_4 particles coated with oleic acid, we assumed that only 30 % of the weight was contributed by oleic acid. Methyl oleate was taken as the model precursor for doing functionalization reactions.*

SI 7.1 Synthesis of methyl oleate

To a stirred solution of oleic acid (10 g, 35 mmol.) in methanol (800 mL, 35 mmol.), 1 - 2 drop of 1 M H_2SO_4 were poured and was refluxed at 60 ^oC for 3 h. After removing excess methanol and washing with saturated solution of $NaHCO_3$ (2 × 100 mL), brine, the organic phase was extracted with $CHCl_3$, dried with Na_2SO_4, filtered and concentrated under vacuum to get a colorless oil that was chromatographed (silica, 100 - 200 mesh; eluent: petroleum ether (PE) / Ethyl acetate (EA) = 95 : 5). Yield: 90%, 9.4 g; thin layer chromatography (TLC): Retention factor (R_f) 0.8 (10 % EA/PE) and characterized by FT-IR (**Fig. S14**) and NMR spectrometer (**Fig. S15, S16**).

FT-IR (cm^{-1}): 3004 (m) (sp^2 C-H stretching vibration), 2954 / 2925 / 2852 (s) (sp^3 C-H stretching vibration), 1755 (s) (C=O stretching vibration), 1465 (m) (CH_2 bending vibration), 1376 (m) (CH_3 bending vibration), 1195 (s) (C-O bending), 720 (s) (long chain band). Here, 's' and 'm' are referred as strong and medium peak intensities, respectively.

^{1}H (400 MHz, $CDCl_3$): δ 5.37-5.28 (m, J = 3.2 Hz, 2H), 3.65 (s, 3H), 2.30-2.27 (t, J = 7.2 Hz, 2H), 2.02 - 1.97 (q, J = 6.4 Hz, 4H), 1.62-1.59 (t, J = 7.2 Hz, 2H), 1.29-1.25 (m, 18H), 0.88-0.87 (d, J = 6.8 Hz, 3H). Here, 'm', 's', 't', 'q' and 'd' are referred as multipeaks, singlet, triplet, quartet, and doublet, respectively.

^{13}C (100 MHz, $CDCl_3$): δ, 174.2, 129.9, 129.7, 51.4, 34.1, 31.9, 29.7, 29.6, 29.5, 29.3, 29.1, 29.0, 27.2, 27.1, 24.9, 22.6, 14.1.

Figure S14. FT-IR spectra of methyl oleate and oleic acid

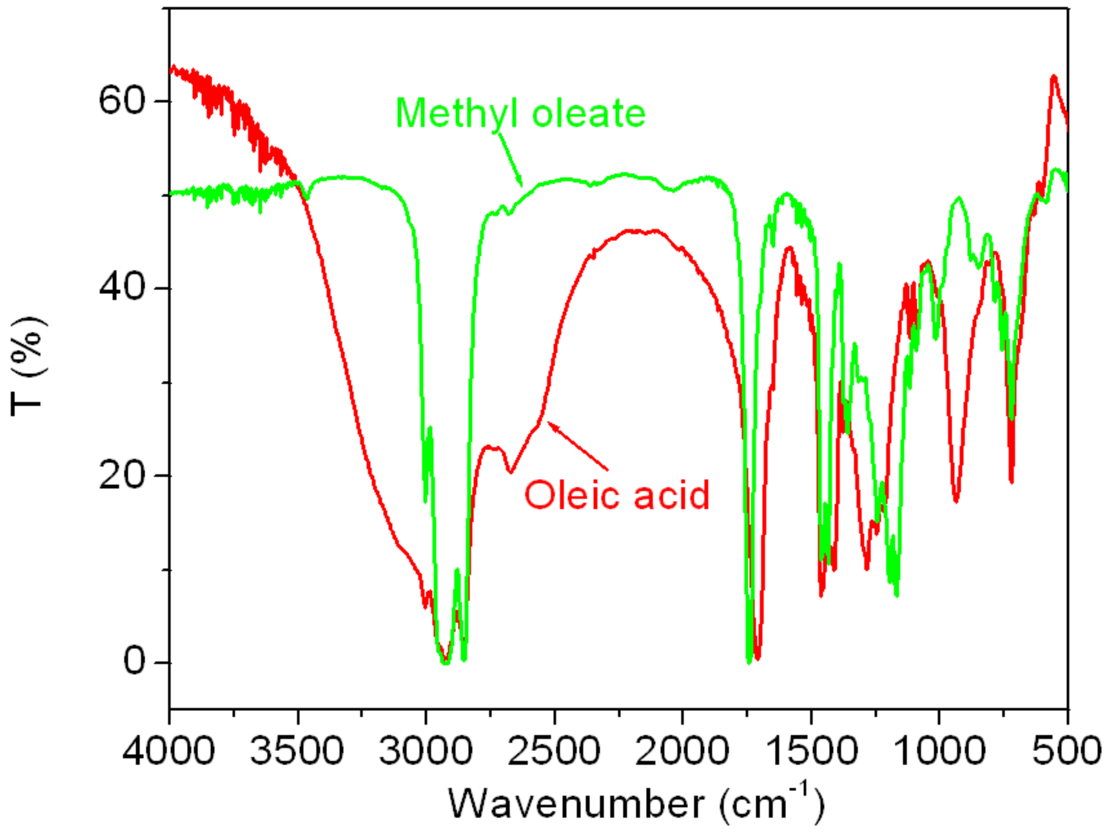

Figure S15. ^{1}H NMR spectrum of methyl oleate

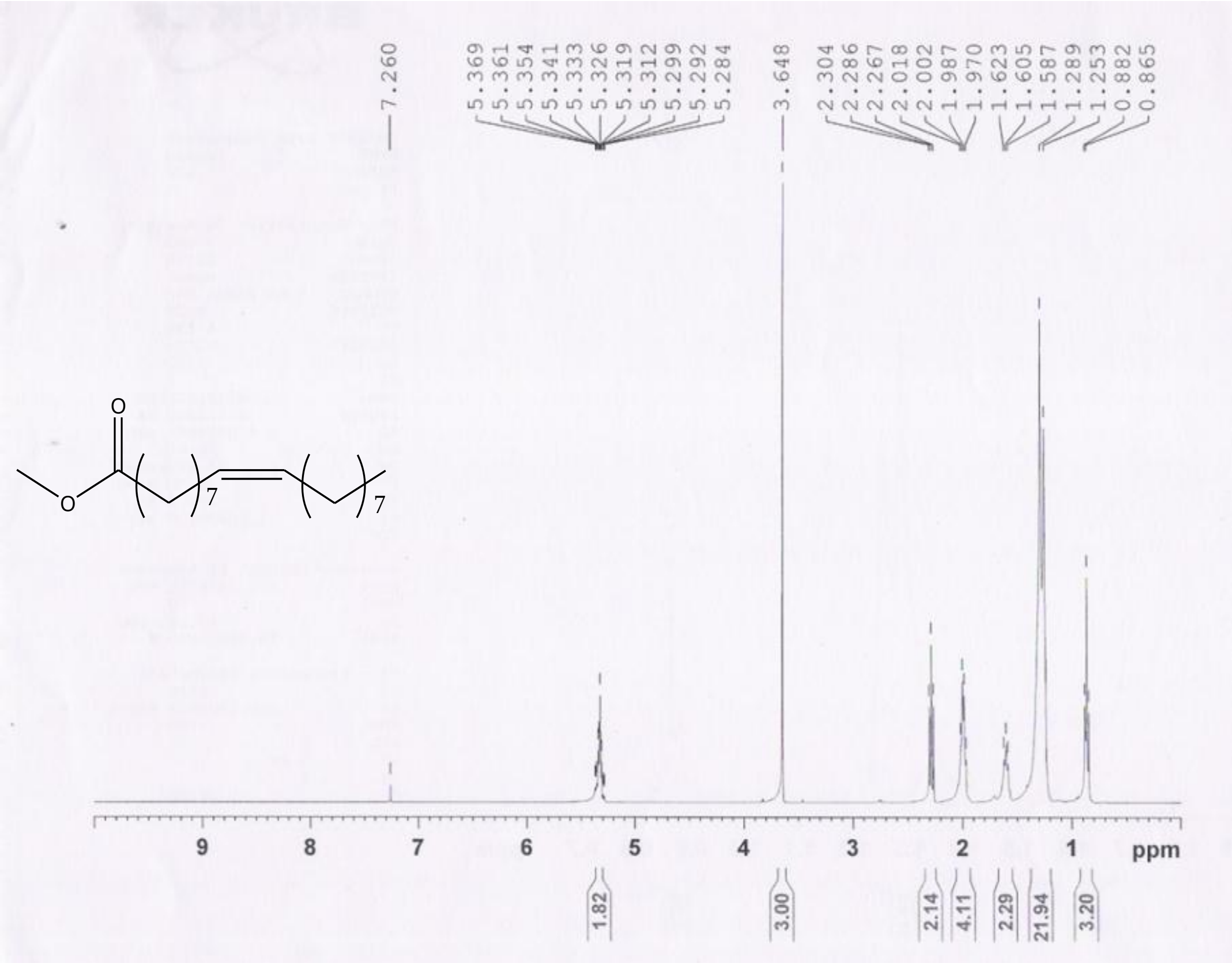

Figure S16. ^{13}C spectrum of methyl oleate

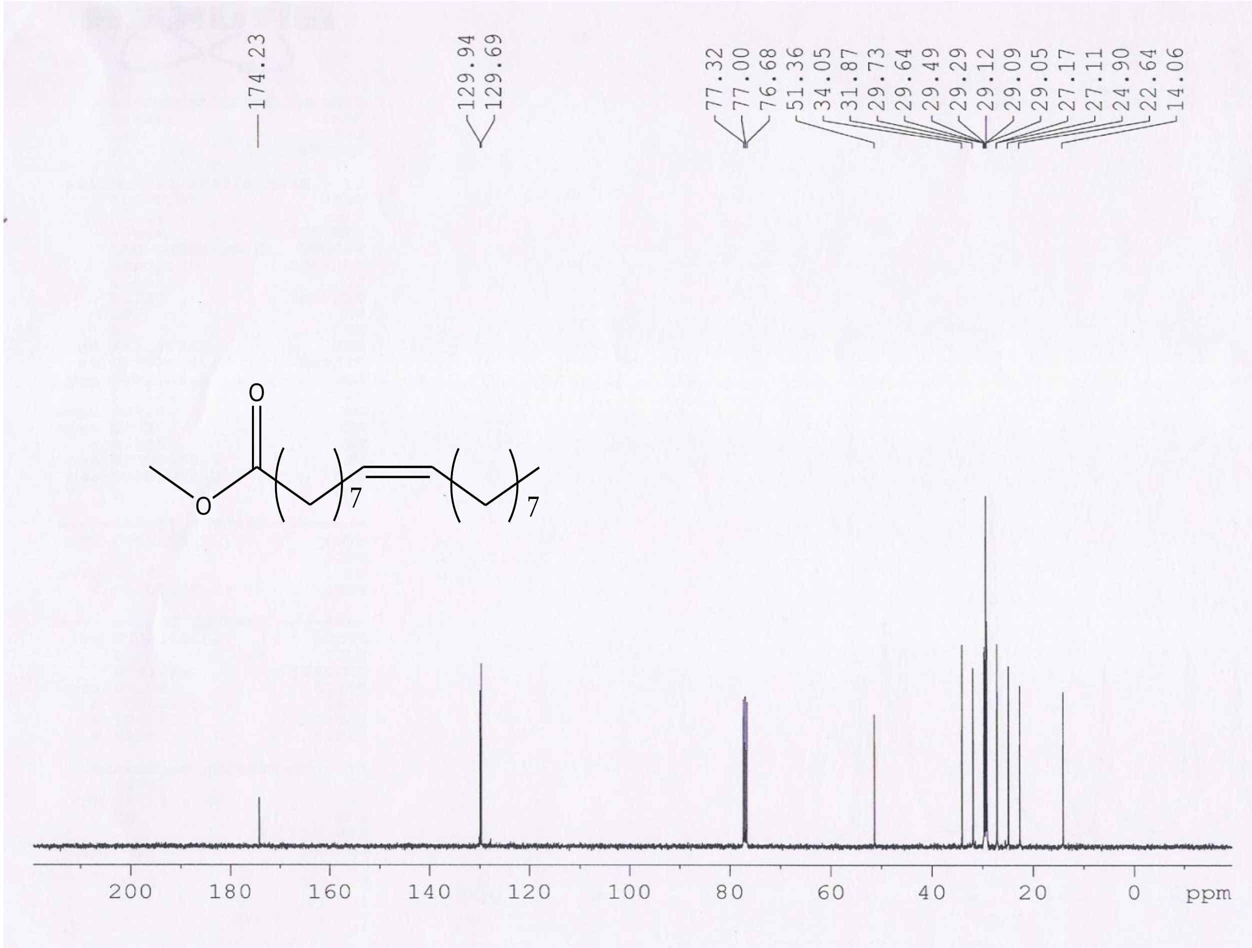

SI 7.2 Functionalization of particles with thioglycolic acid

A. Reaction of thioglycolic acid with methyl oleate

To a solution of methyl oleate (500 mg, 1.7 mmol) in hexane (5 mL), thioglycolic acid (0.143 mL, 2 mmol.) was added. After exposing to the ultraviolet lamp (285 nm) for 3 h, the mixture was washed with saturated aqueous solution of $NaHCO_3$ (30 mL), extracted with $CHCl_3$, dried with Na_2SO_4, filtered and concentrated under vacuum to get a colorless oil that was chromatographed (silica 100 - 200 mesh; eluent: PE/EA = 90:10) and characterized by FT-IR (**Fig. S17**) and NMR spectroscopy (**Fig. S18, S19**). Yield : 90 %, 0.524 ; TLC : R_f 0.4 (15 % EA / PE).

FT-IR (cm^{-1}): 3413-2721 (broad band) (O-H stretching vibration of carboxyl group), 2936 / 2854 (s) (sp^3 C-H stretching vibration), 1738 (s) (C=O stretching vibration of ester), 1714 (s) (C=O stretching vibration of carboxyl group), 1460 (m) (CH_2 bending vibration), 1357 (m) (CH_3 bending vibration), 1192 (C-O bending), 721 (w) (long chain band).

^{1}H (300 MHz, $CDCl_3$): δ 3.67 (s, 3H), 3.33 - 3.24 (m, 5H), 3.24 (s, 1H), 2.80 - 2.75 (t, J = 6.3 Hz, 2H), 2.34 - 2.29 (t, J = 7.2 Hz, 2H), 1.62 - 1.53 (m, 7H), 1.41 - 1.39 (m, 22H), 0.90 - 0.87 (t, J = 6.3 Hz, 3H).

^{13}C (75 MHz, $CDCl_3$): δ 175.9, 175.9, 174.6, 174.6, 51.6, 51.5, 46.7, 46.7, 34.4, 34.3, 34.2, 34.1, 34.1, 32.2, 31.9, 31.9, 29.6, 29.5, 29.4, 29.3, 29.3, 29.2, 29.2, 29.1, 29.1, 28.9, 26.6.

Figure S17. FT-IR spectra of thioglycolic acid addition to C-C double bond of oleic acid. Inset shows the spectra region from 2000 to 1500 cm^{-1}.

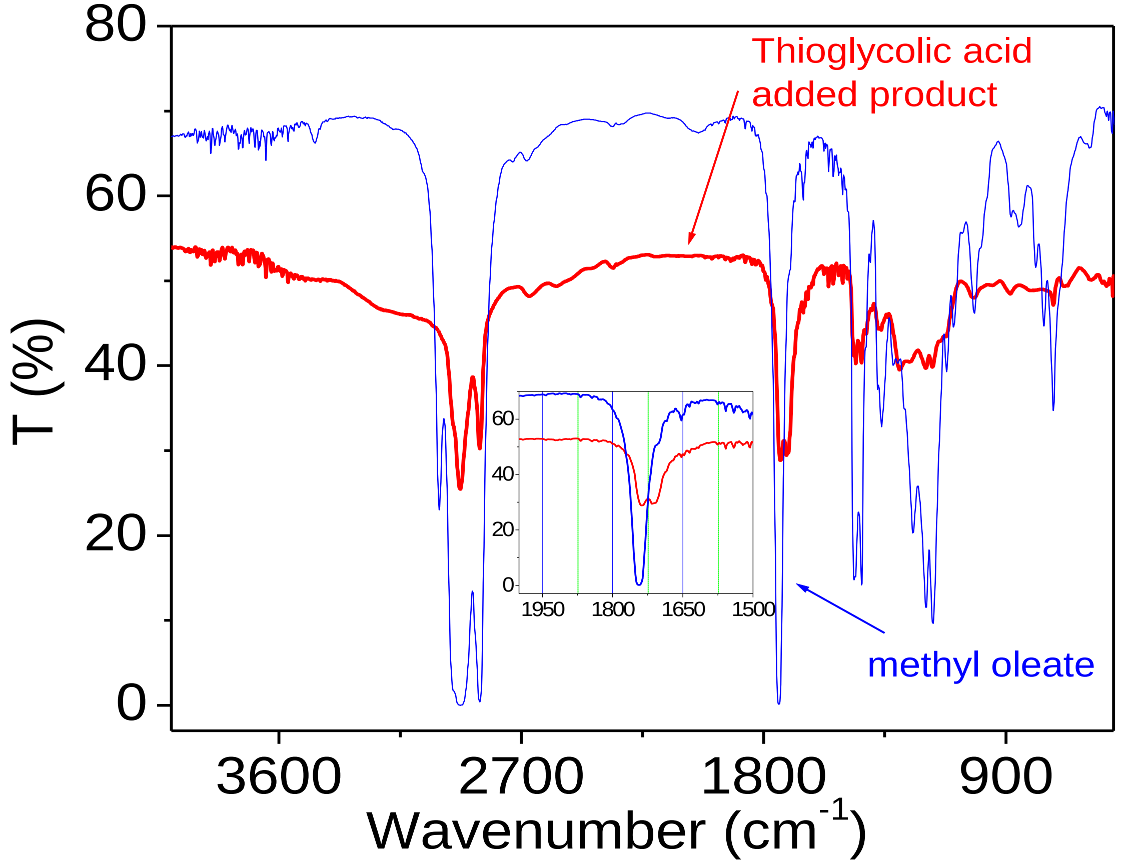

Figure S18. ^{1}H NMR spectrum of thioglycolic acid addition to C-C double bond of oleic acid

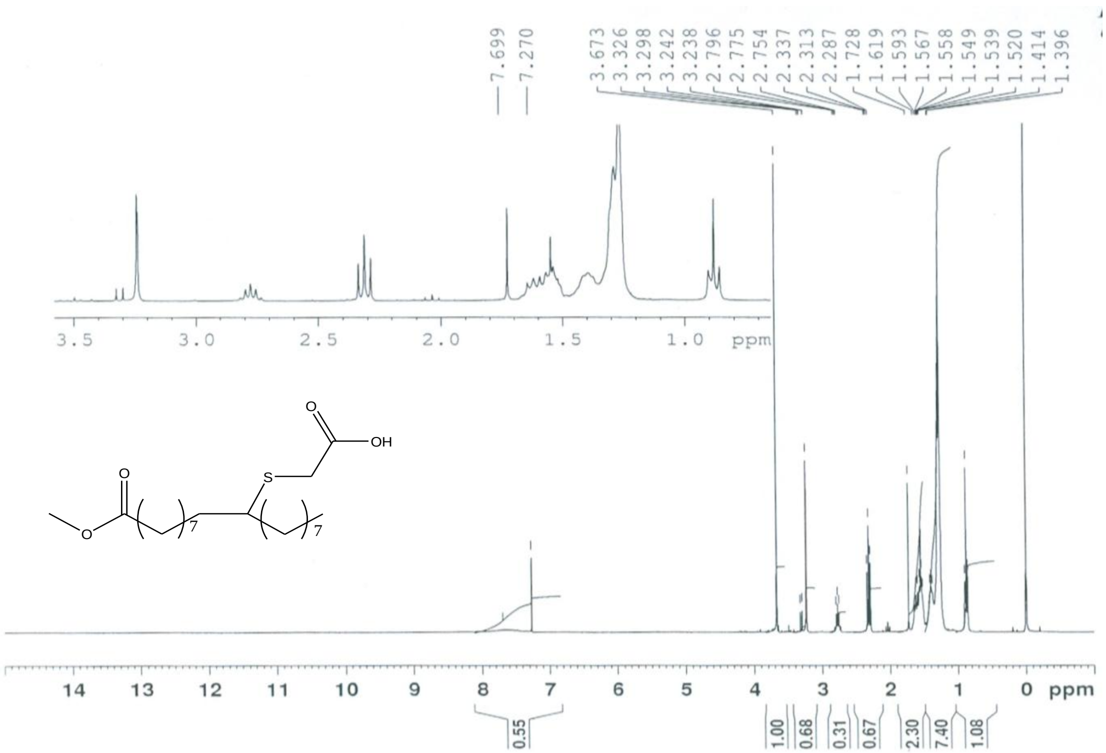

Figure S19. ^{13}C NMR spectrum of thioglycolic acid addition to C-C double bond of methyl oleate

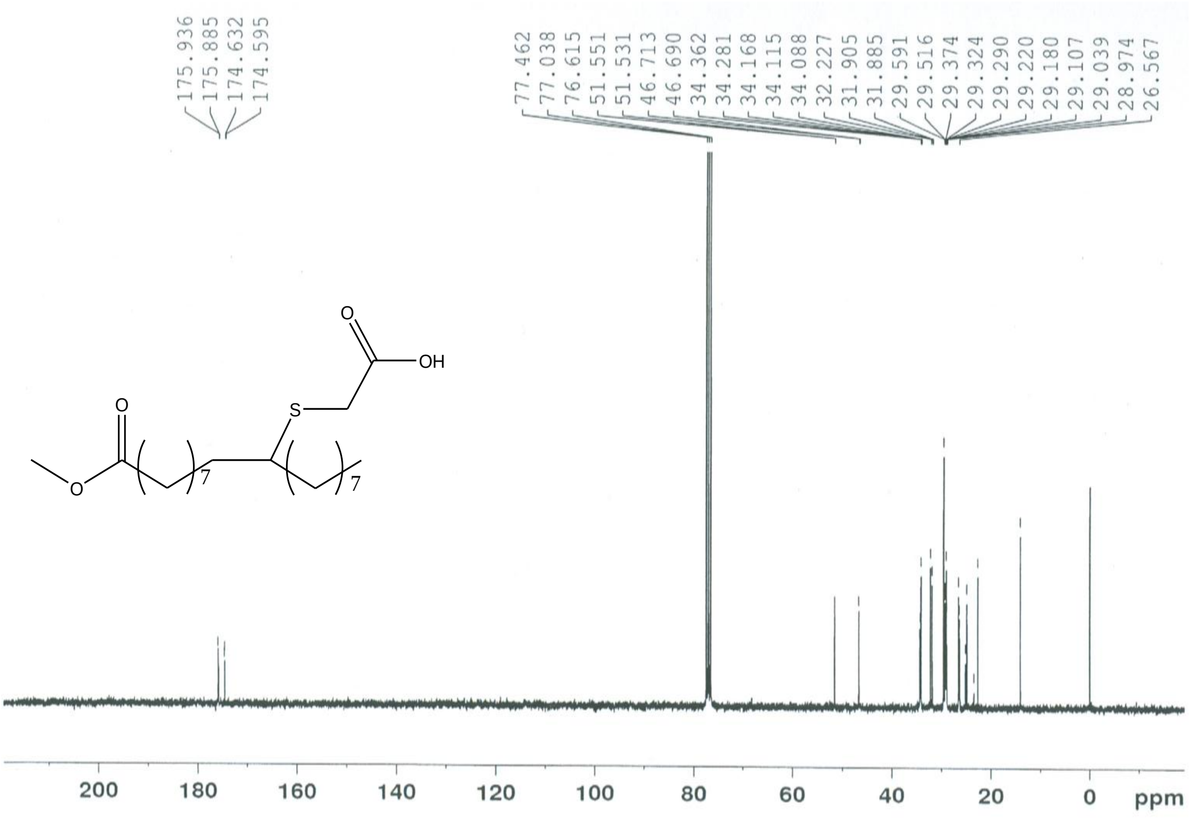

B. Conjugation of Fe_3O_4 particles with thioglycolic acid

To a quartz tube containing Fe_3O_4 particles (100 mg) dispersed in hexane (5 mL), and thioglycolic acid (11.2 μL, 0.159 mmol., 1.5 equivalent) in isopropanol (2 mL) were added and exposed to the ultraviolet light (285 nm) for about 3 h. The particle was centrifuged, washed with methanol three times after removing the solvent and thioglycolic acid under reduced pressure, dried and characterized by FT-IR spectroscopy (**Fig. S20**).

FT-IR (cm^{-1}): 3690 - 2595 (broad band) (O-H stretching vibration of carboxyl group), 1684 (broad band) (C=O stretching vibration of carboxyl group), 1562 (w) (COO^- asymmetric stretching vibration), 1453 (m) (CH_2 bending vibration), 1407 (s) (COO^- symmetric stretching vibration) 1294 (m) (C-O stretching vibration), 911 (m) (O-H loop), 547 (m) (Fe-O vibration).

Figure S20. FT-IR spectra of Fe_3O_4 particles functionalized with thioglycolic acid addition to C-C double bond of capping oleic acid and Fe_3O_4 particles

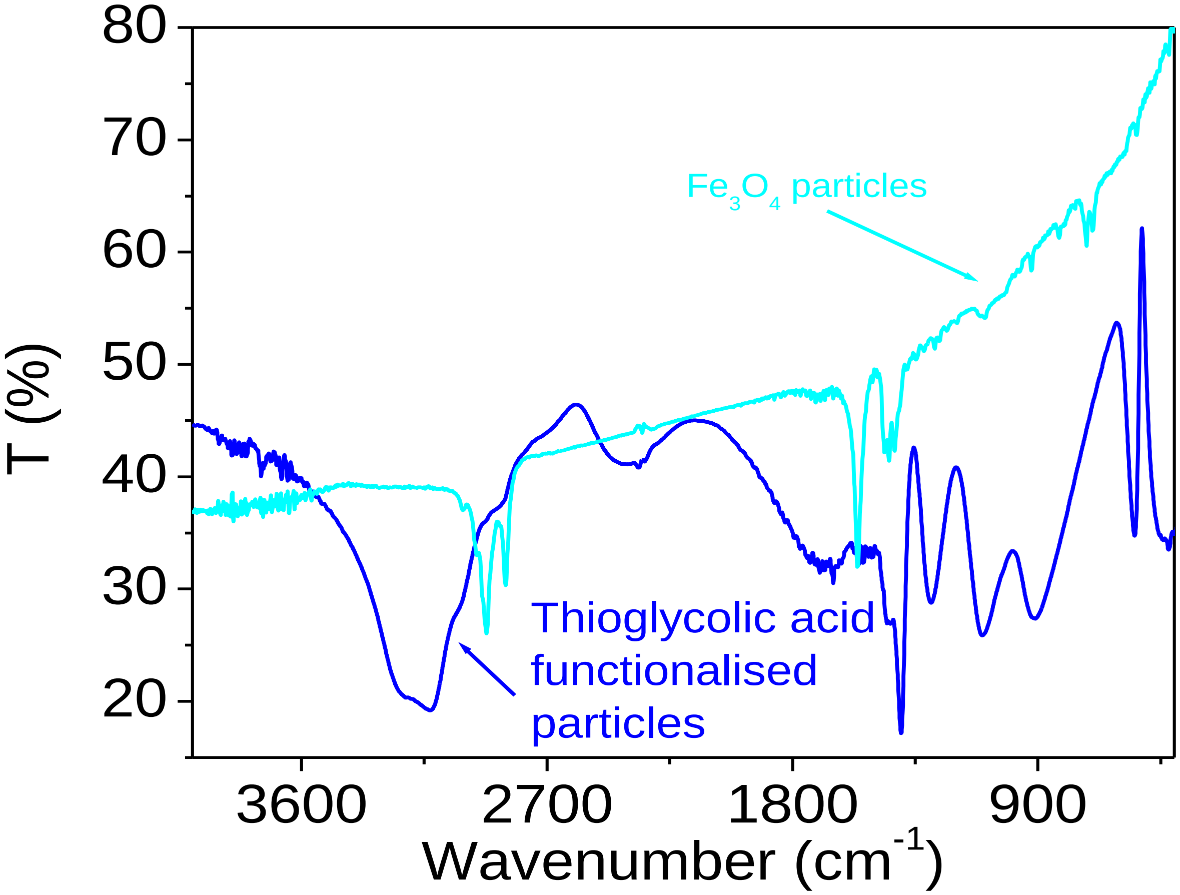

SI 7.3 Functionalization of Fe_3O_4 particles with aspartic acid

A. Reaction with methyl oleate

A.1 Epoxidation of methyl oleate with meta-chloroperbenzoic acid

To a stirred solution of methyl oleate (1 g, 3.37 mmol.) in CH_2Cl_2 (20 mL) at 0 ^oC, a solution of meta-chloroperbenzoic acid (1.25 g, 5.06 mmol, 1.5 equivalent) in CH_2Cl_2 (10 mL) was added slowly. After stirring for 3 h, the reaction mixture was washed with saturated solution of $NaHCO_3$ (20 mL), brine, extracted with $CHCl_3$, dried with Na_2SO_4, filtered and concentrated under vacuum to get a colorless oil that was chromatographed (silica:100 - 200 mesh; eluent: petroleum ether (PE)/Ethyl acetate (EA) = 90:10) to give epoxy methyl oleate. Yield: 85%, 0.89 g; TLC: R_f 0.6. The obtained compound was characterized by FTIR (**Fig. S21**) and NMR spectroscopy (**Fig. S22, S23**)

FT-IR (cm^{-1}): 2927 / 2854 (s) (sp^3 C-H stretching vibration), 1746 (s) (C=O stretching vibration of ester), 1464 (m) (CH_2 bending vibration), 1369 (m) (CH_2 bending vibration), 1197 (s) (C-O bending vibration of ester), 723 (s) (long chain band).

^1H (300 MHz, $CDCl_3$): δ 3.66 (s, 3H), 2.89 - 2.88 (m, 2H), 2.32 - 2.28 (t, J = 7.5 Hz, 2H), 1.43 (bs, 2H), 1.42 - 1.27 (m, 24H), 0.94 - 0.85 (t, J = 6.3 Hz, 3H).

^{13}C (75 MHz, $CDCl_3$): δ 174.2, 57.2, 57.1, 51.4, 34.1, 31.9, 29.6, 29.4, 29.3, 29.2, 29.1, 27.9, 27.8, 26.6, 26.6, 24.9, 22.7, 14.1.

Figure S21. FT-IR spectra of epoxy methyl oleate and methyl oleate. Inset shows the spectral region from 3050 to 2850 cm^{-1}.

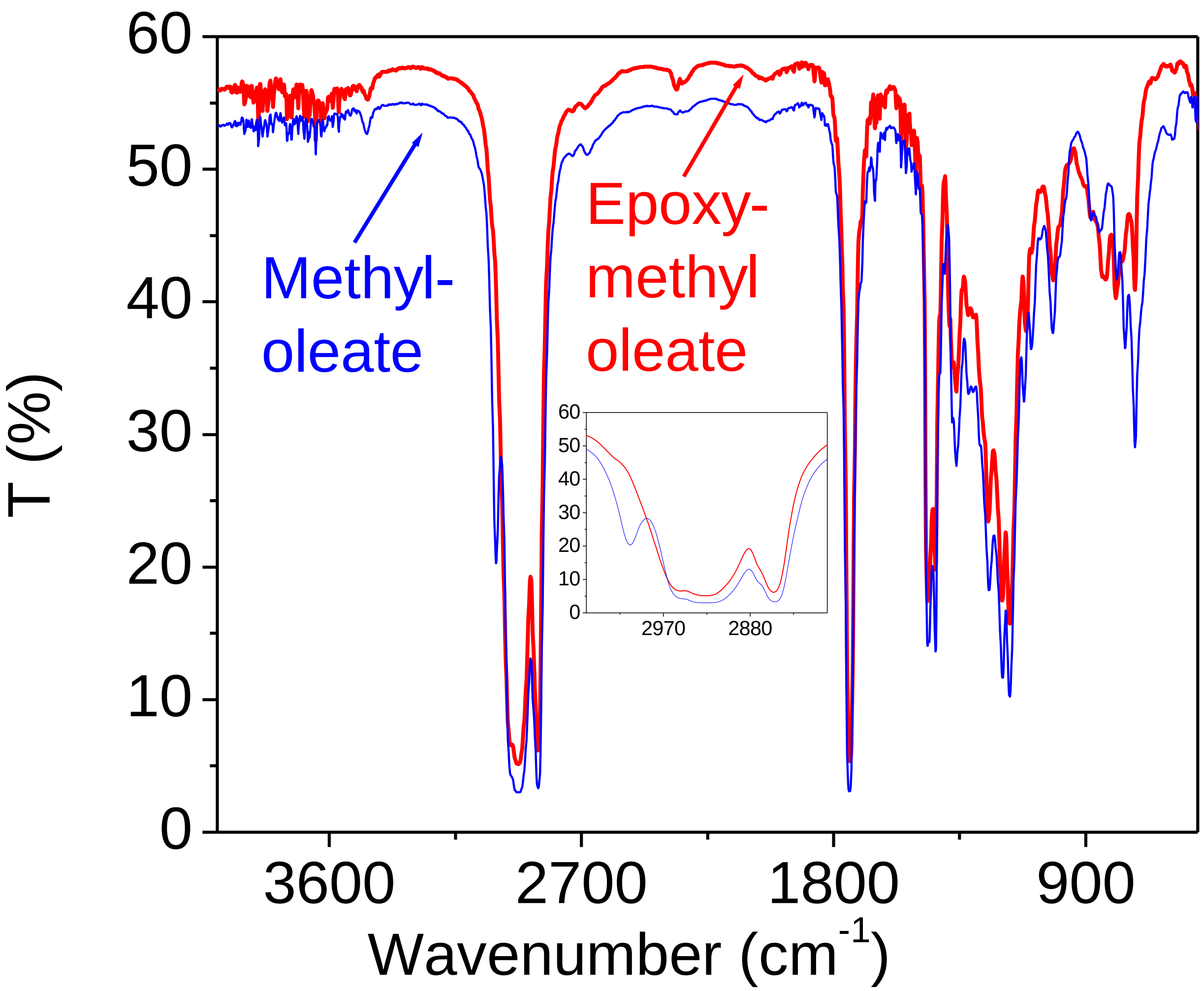

Figure S22.^{1}H NMR spectrum of epoxide of methyl oleate. Inset shows expansion spectrum in 0.6-3.0 ppm.

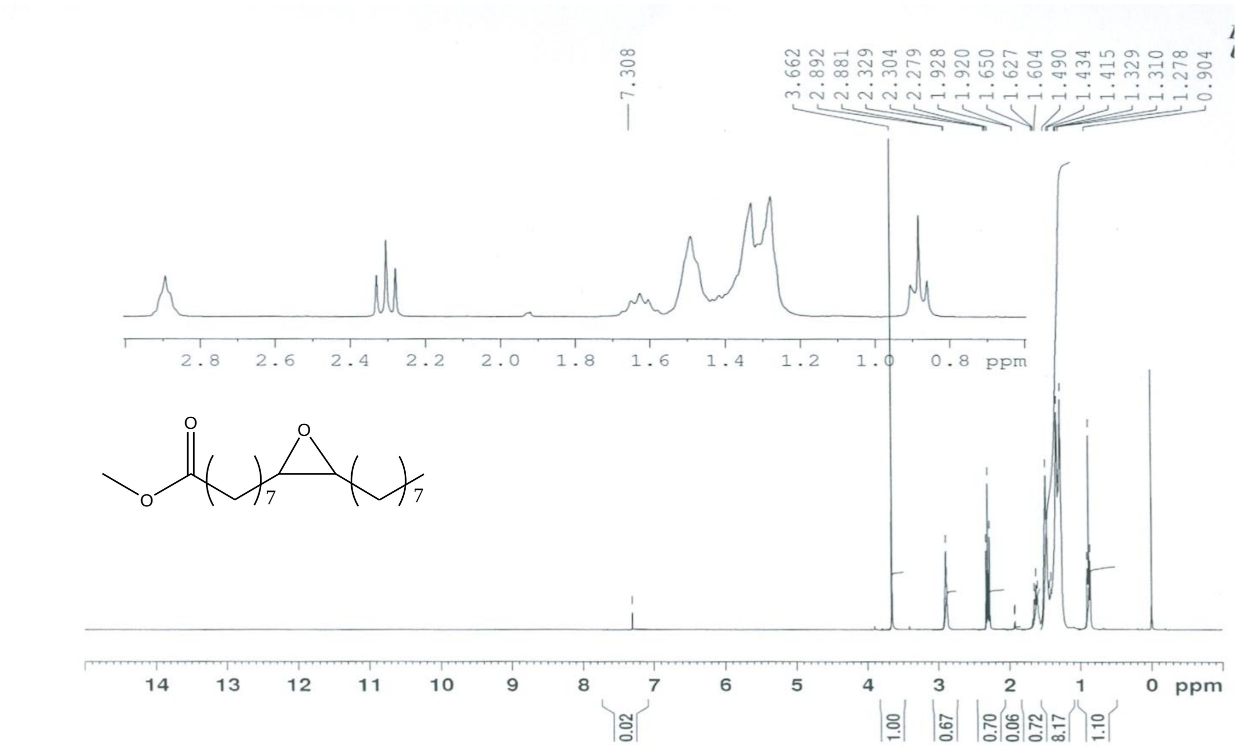

Figure S23. ^{13}C NMR spectrum of epoxide of methyl oleate

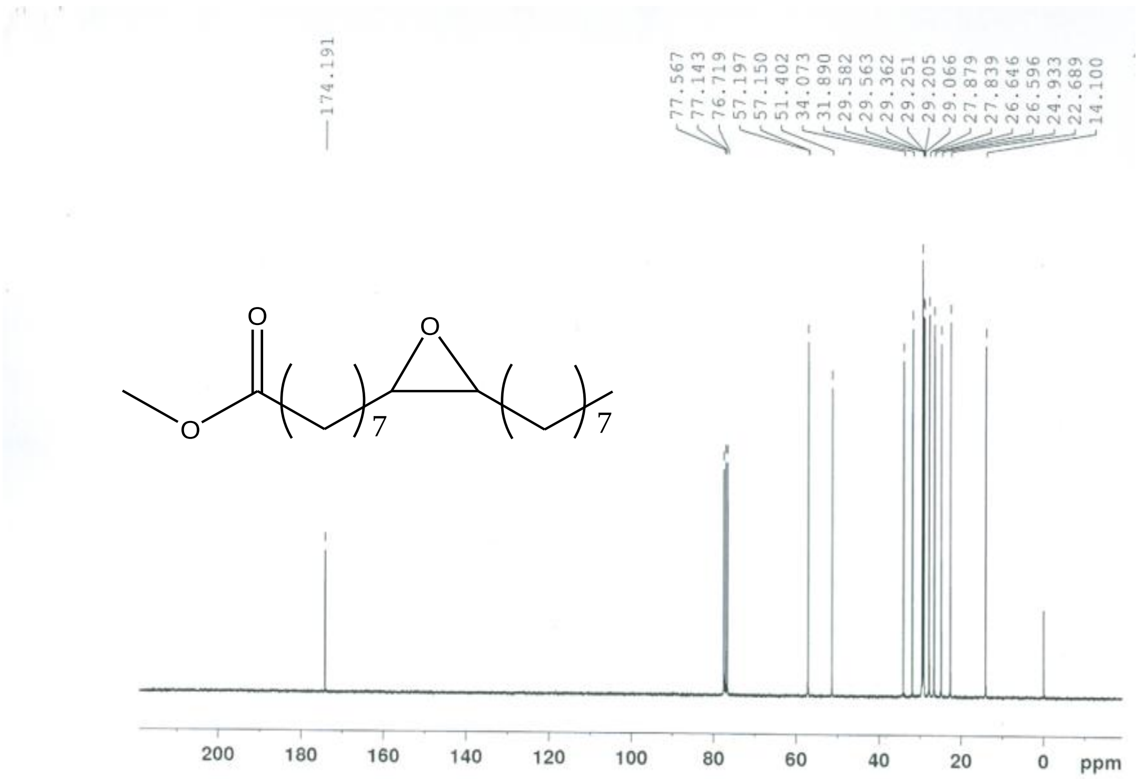

A.2 Ring opening of epoxide of methyl oleate by protected aspartic acid

To a solution of epoxy methyl oleate (500 mg, 1.6 mmol.) in dimethyl formamide (DMF) (10 mL), aspartic acid (320 mg, 2.4 mmol. 1.5 equivalent) and 1 drop of BF_3 were added and the mixture was refluxed at 60 ^oC. After refluxing for 3 h and cooling to the room temperature, the mixture was washed with saturated solution of $NaHCO_3$ (50 mL), brine, extracted with $CHCl_3$, washed five times with water, dried with Na_2SO_4, filtered and concentrated under vacuum to get a colorless oil that was chromatographed (silica: 100 - 200 mesh; eluent: hexane / ethyl acetate = 50:50) to get a white solid powder. yield 73%, 0.42 g); TLC R_f 0.45 (30 % EA / PE). The obtained compound was characterized with FT-IR (**Fig. S25**) and NMR spectroscopy (**Fig. S26, S27**).

FT-IR (neat, cm^{-1}): 3469 (broad) (hydrogen bonded O-H stretching vibration), 3381 (s) (N-H stretching vibration of amines), 3182 (m) (overtone of 1629), 2961/2897/2843 (s) (sp^3 C-H stretching vibration), 1764 - 1710 (s) (C=O stretching vibration), 1631 (m) (N-H bending vibration), 1459 (m) (CH_2 bending vibration), 1376 (m) (CH_3 bending vibration), 1200 (m) (C-O stretching vibration), 1115 (m) (C-N stretching vibration), 886 (m) (N-H loop), 723 (long chain band).

^{1}H (300MHz, $CDCl_3$): δ 8.09 (bs, 1H), 4.86 (bs,1H) 3.60 (s, 3H), 3.55 (bs, 1H), 2.26-2.21 (bs, 3H), 1.57 (bs, 4H), 1.38 (bs, 4H), 1.24-2.20 (bs, 18H), 0.81 (bs, 3H).

^{13}C (75MHz, $CDCl_3$): δ 174.3, 161.0, 76.7, 72.0, 51.4, 33.9, 33.3, 31.8, 30.4, 29.4, 29.1, 29.0, 28.9, 25.5, 25.4, 25.3, 25.2, 24.8.

Figure S25. FT-IR spectrum of epoxide ring opening by aspartic acid

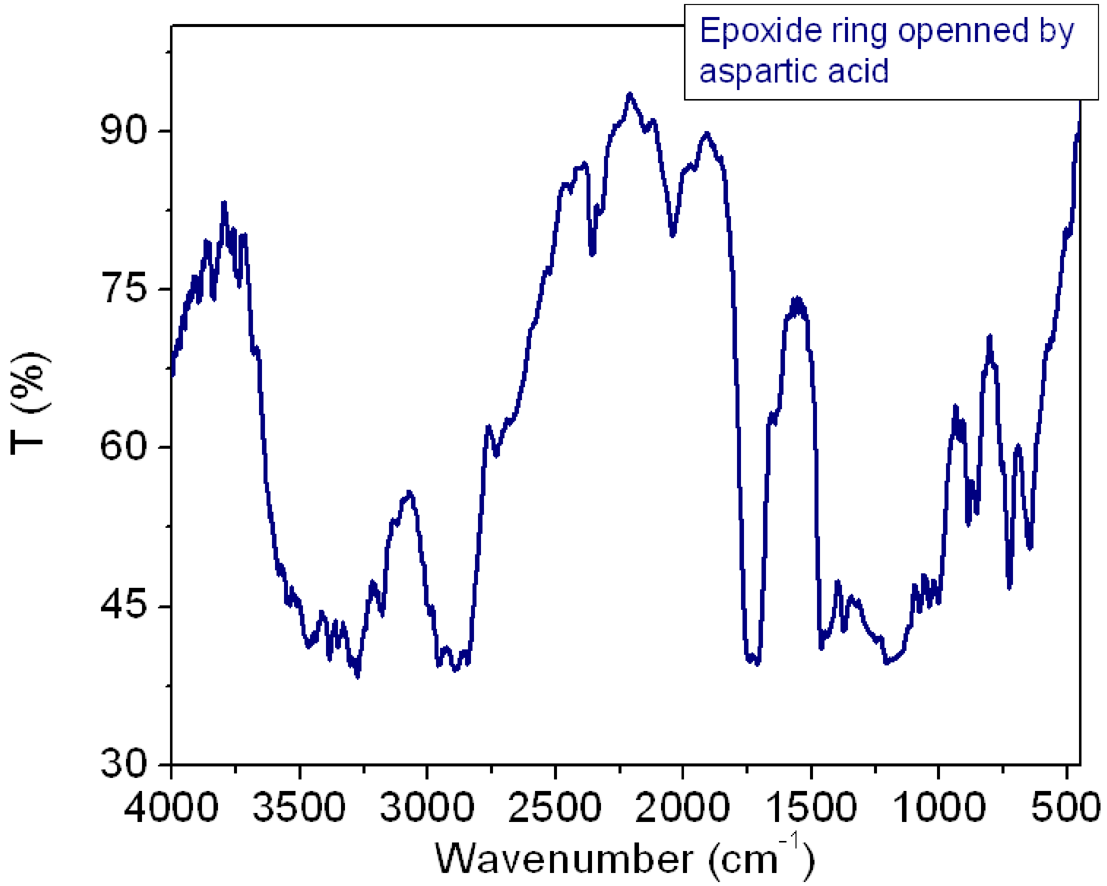

Figure S26. ¹H NMR spectrum of epoxide ring opened by aspartic acid

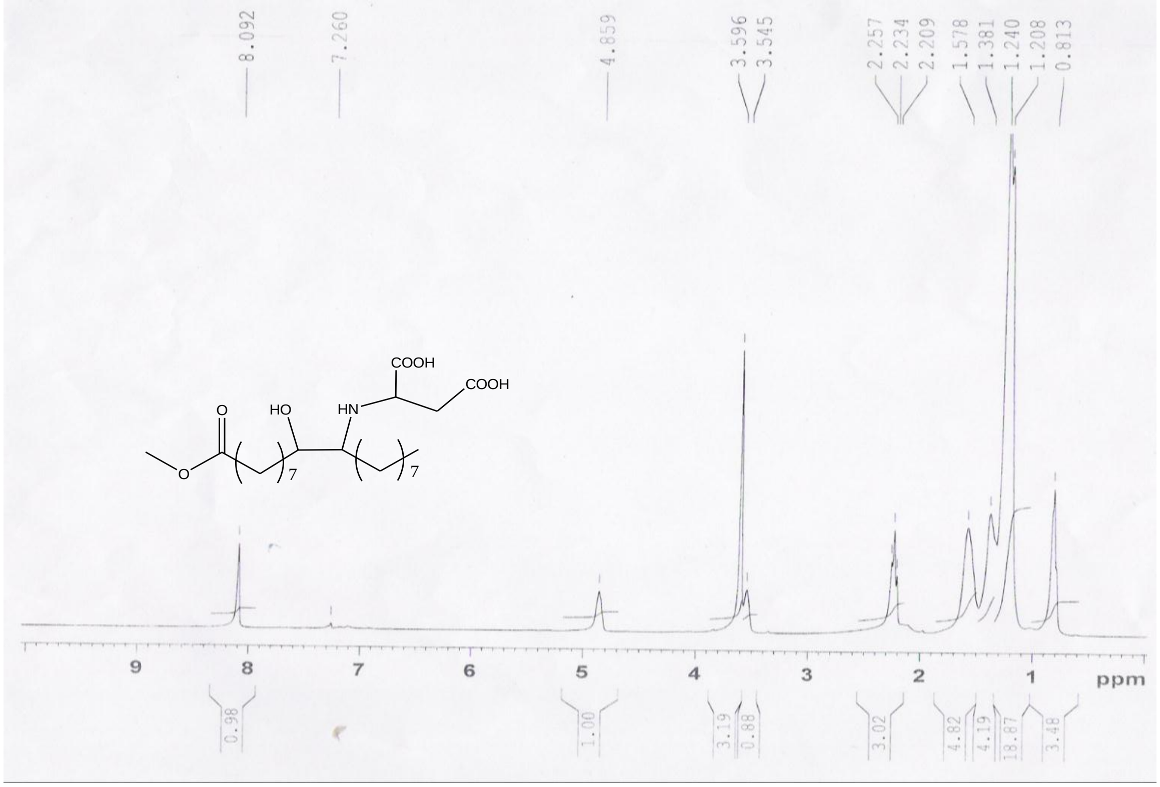

Figure S27. ^{13}C NMR spectrum of epoxide ring by aspartic acid

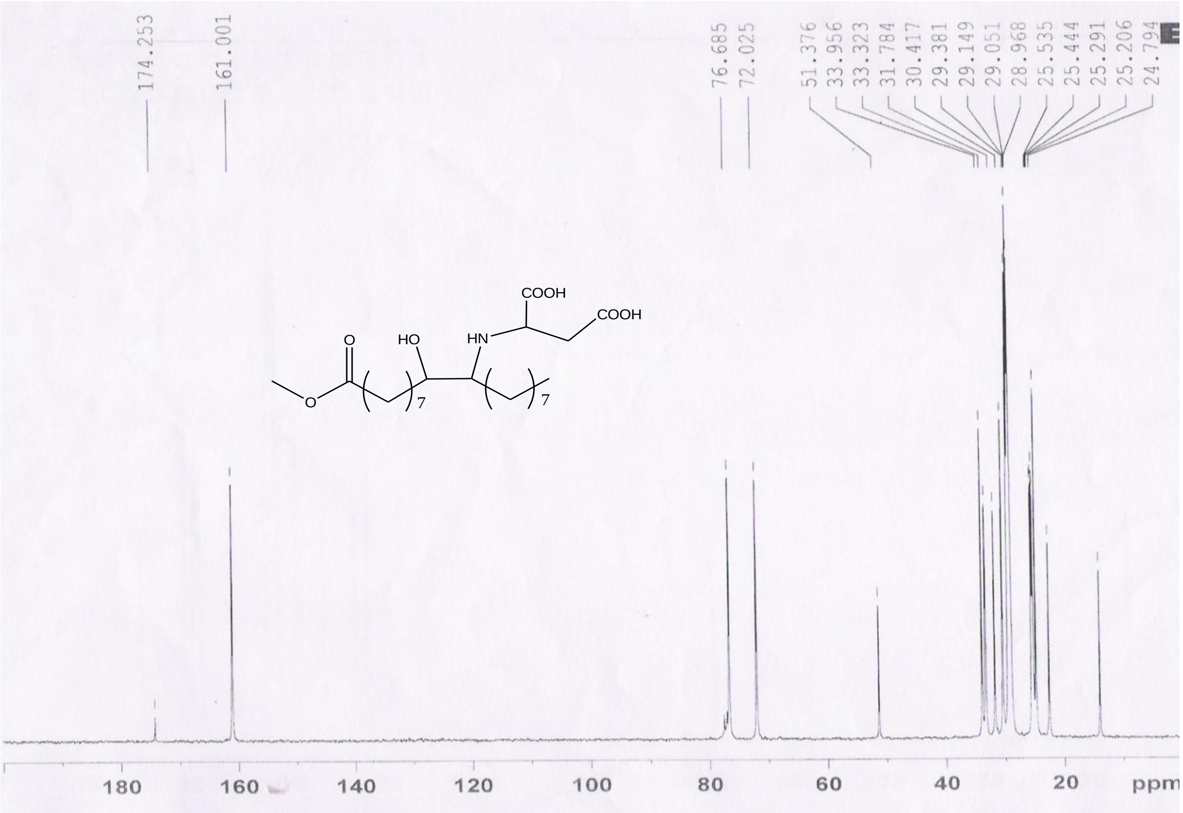

B. Conjugation of Fe_3O_4 particles with aspartic acid

B.1 Epoxidation of Fe_3O_4 particles

To a three necked flask containing Fe_3O_4 particles (100 mg) dispersed in dichloromethane (DCM, 30 mL) cooled to 0 ^oC by using ice, meta-chloroperbenzoic acid (87 mg, 0.5 mmol.) dissolved in DCM and dried by anhydrous sodium sulphate was added by dropping funnel. After stirring for 3 hours at room temperature, the reaction was worked up with chloroform, washed three times with water containing sodium bicarbonate, centrifuged with ethanol three times and dried under reduced pressure. The particle was characterized by FT-IR spectroscopy (**Fig. S28**). The yield of the reaction is 85%.

FT-IR (neat, cm^{-1}): 3433 (broad) (O-H stretching vibration of water molecule), 2931 / 2860 (s) (sp^3 C-H stretching vibration), 1647 (asymmetric $–COO^-$ stretching vibration), 1401 (symmetric $–COO^-$ stretching vibration), 1119 (m) (C-O stretching vibration of oxirane), 583 (s) (Fe-O stretching vibration).

Figure S28. FT-IR spectrum of epoxidation of Fe_3O_4 nanoparticles

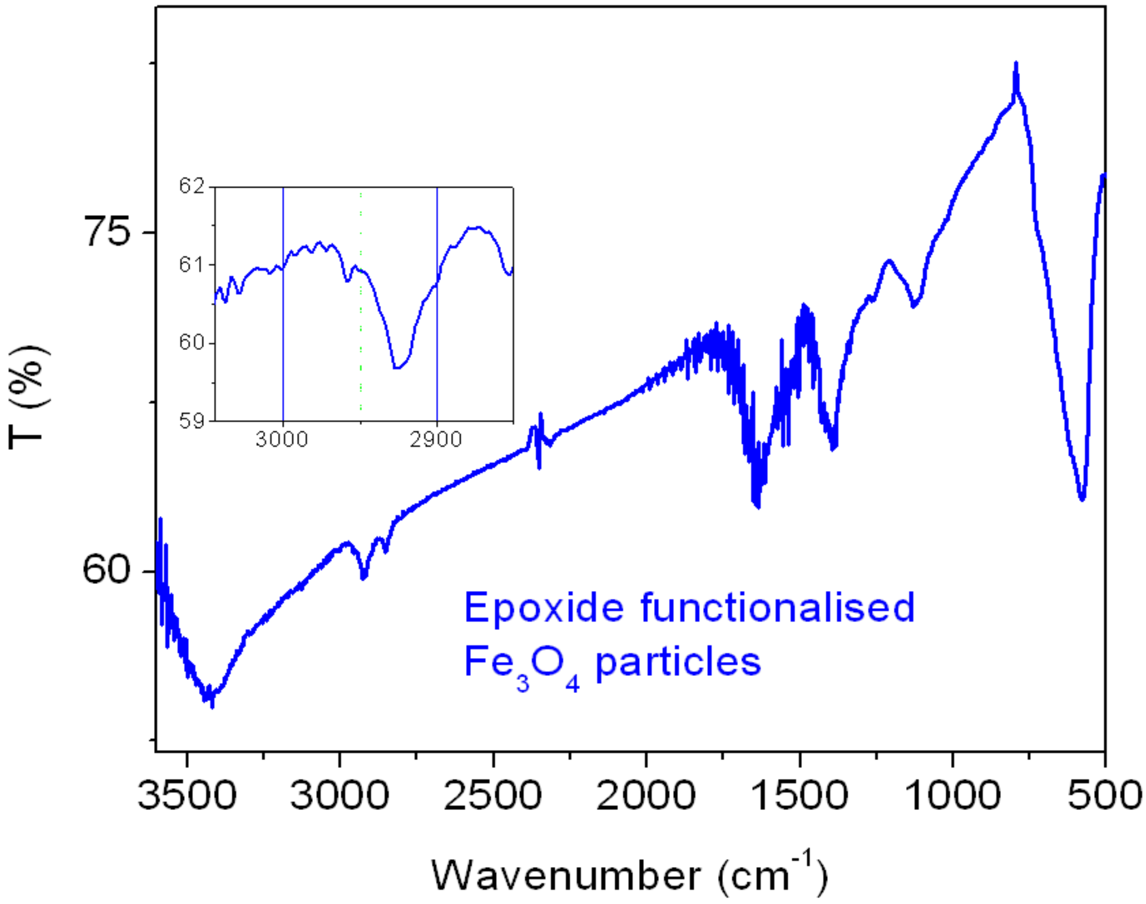

B.2 Epoxide ring opening by aspartic acid:

To the epoxide functionalized particles (50 mg) in DMF (5 mL), aspartic acid (3.2 mg), 1 - 2 drops of boron trifluoride were added and refluxed at the temperature of 60 ^{o}C for 3 h. After heating for 1 h no phase separation was observed and even after cooling also. The particles were centrifuged, washed with ethyl acetate and then methanol three times each, dried under reduced pressure and characterized by FT-IR spectrometer (**Fig. S29**). The yield of the reaction was 80%.

FT-IR (cm^{-1}): 3447 - 1685 (broad) (O-H stretching vibration of the carboxyl group), 2921 / 2854 (sp^3 C-H stretching vibration), 1570 (s) (N-H bending and COO^- stretching vibration), 1396 (m) (COO^- asymmetric stretching vibration), 1365 (w) (CH_3 bending vibration), 1126 (m) (C-N stretching vibration), 794 (w) (N-H loop), 570 (s) (Fe-O vibration).

Figure S29. FT-IR spectrum of Fe_3O_4 nanoparticles after epoxide ring on ligand opened by aspartic acid

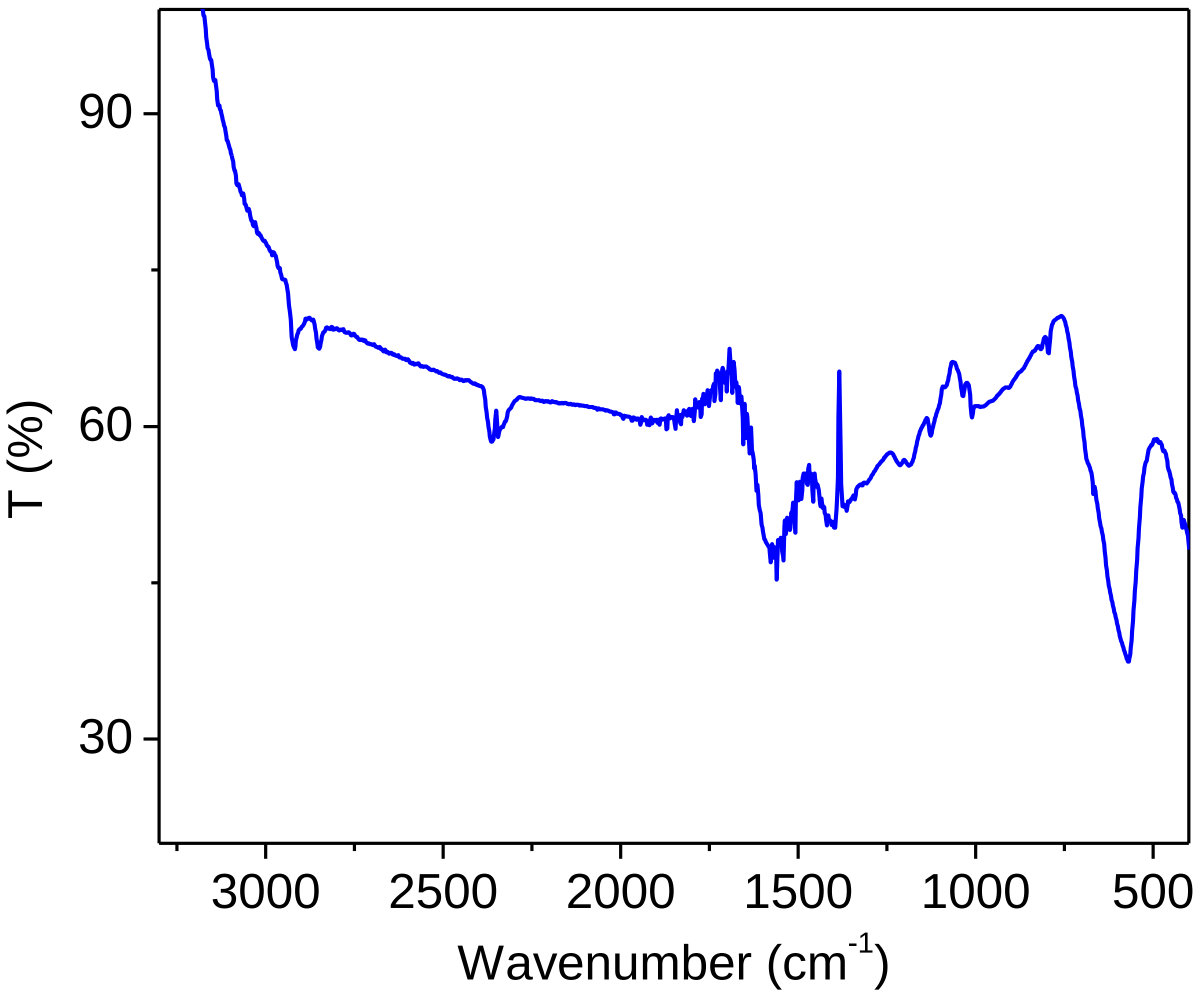

SI. 7.4 Functionalization of Fe_3O_4 particles by aminophosphonate functional group

A. Reactions on methyl oleate

A.1 Ozonolysis of methyl oleate:

To a three necked flask containing a stirred solution of methyl oleate (5 g, 16.86 mmol.) in CH_2Cl_2 (50 mL) clamped inside at a Dewar flask, oxygen gas was passed with simultaneous cooling to - 40 ^oC. Maintaining this temperature, ozone was passed till bluish coloration appeared. Passing oxygen only, triphenyl phosphine (6.63 g, 25.3 mmol.) was added. After stirring for overnight at room temperature, the mixture was concentrated and chromatographed (silica: 100 - 200 mesh; eluent: PE / EA = 95:5). Yield: 95 %, 2.79 g; TLC: R_f 0.6 (5 % EA / PE). The obtained compound was characterized by FT-IR (**Fig. S30**) and NMR spectroscopy (**Fig. S31, S32**).

FT-IR (neat, cm^{-1}): 2934 / 2857 (s) (sp^3 C-H stretching vibration), 2717 (s) (C-H stretching vibration of –CHO), 1738 (s) (C=O stretching vibration of ester), 1711 (s) (C=O stretching vibration of aldehyde), 1461 (m) (C-H bending of CH_2), 1362 (C-H bending vibration of CH_3), 1198 (C-O bending vibration), 729 (m) (long chain band).

^{1}H (300 MHz, $CDCl_3$) δ 9.74 (s, 1H), 3.65 (bs, 3H), 2.43 - 2.39 9 (m, 1H), 2.35 - 2.26 (m, 1H), 1.6 (bs, 4H), 1.30 (bs, 8H).

^{13}C (300 MHz, $CDCl_3$): δ 202.9, 174.4, 51.5, 43.8, 34.3, 34.0, 28.9, 24.8, 24.6, 23.5, 21.9, 14.1.

Figure S30. FT-IR spectra of aldehyde obtained by ozonolysis and methyl oleate

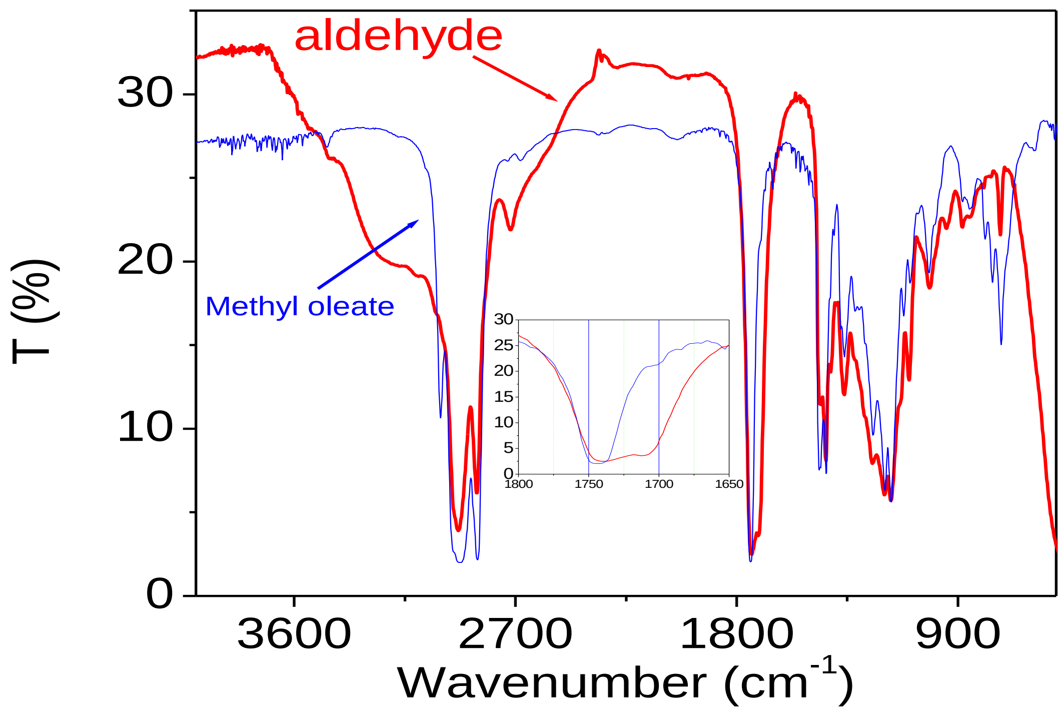

Figure S31. ^{1}H spectrum of aldehyde obtained by ozonolysis

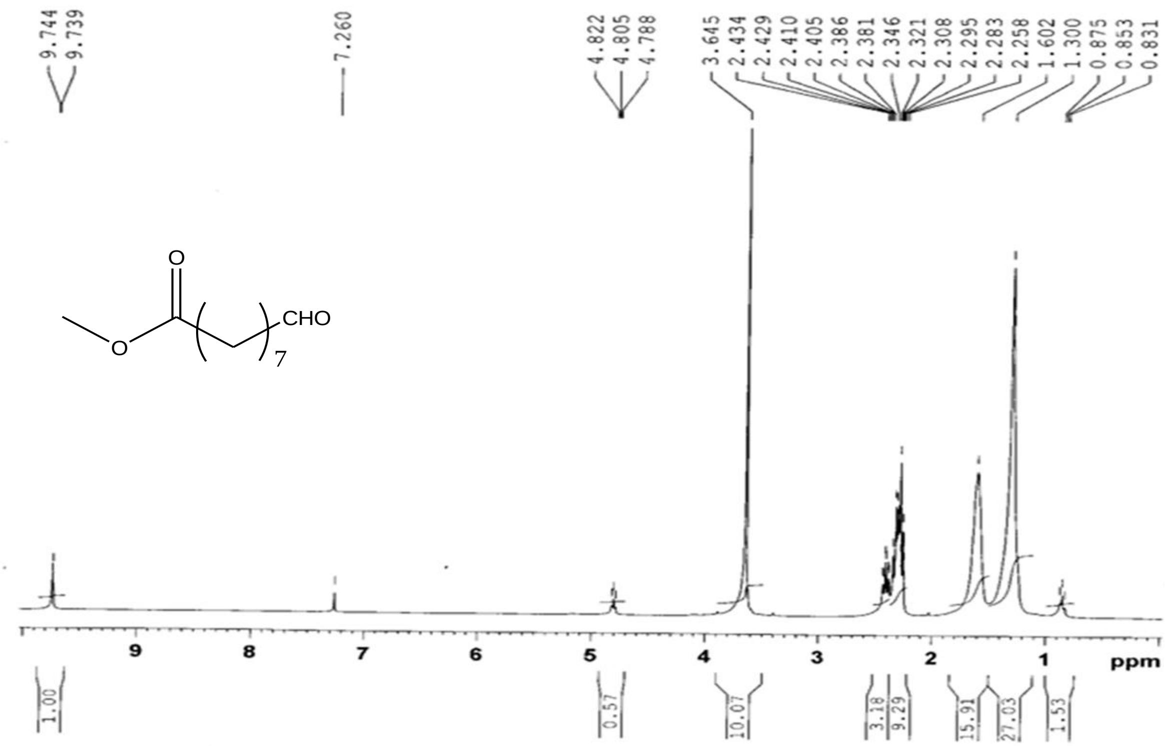

Figure S32. ^{13}C NMR spectrum of aldehyde obtained by ozonolysis

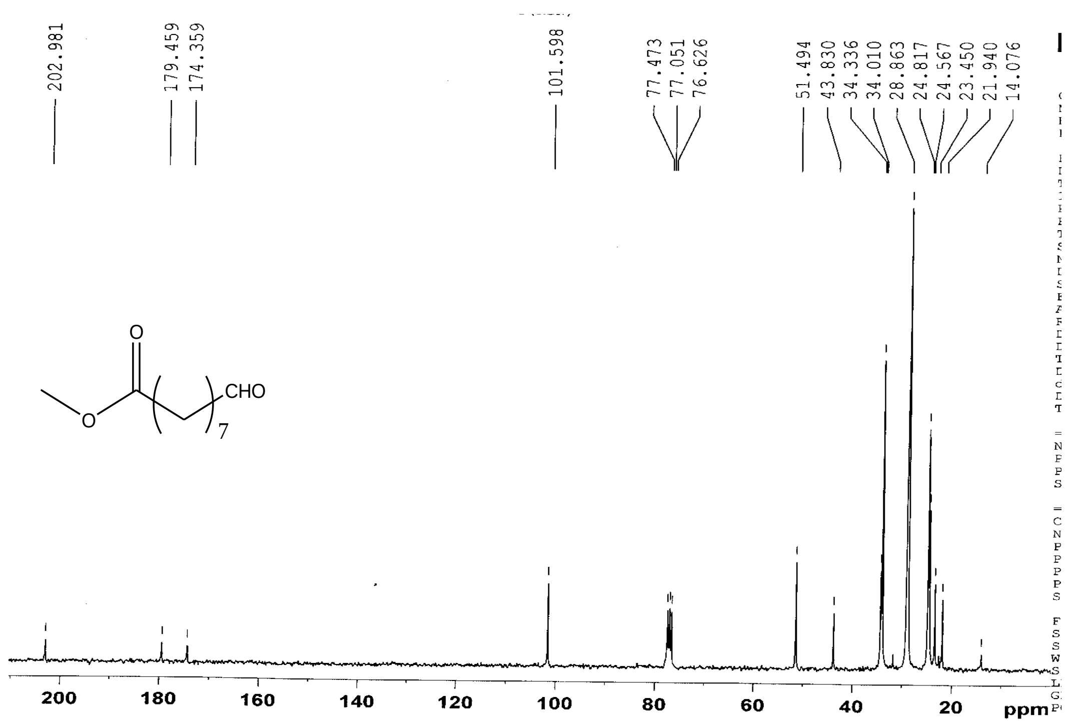

A.2 Synthesis of aminophosphonates starting from aldehyde obtained from ozonolysis.

To a stirred solution of aldehyde (500 mg, 2.7 mmol.) in acetronitrile (10 mL) at 0 ^{o}C, tertiary butyl amine (200 mg, 2.7 mmol.) was added and the mixture was stirred at room temperature. After stirring for 2 h, diethyl phosphite (370 mg, 2.7 mmol.) was added and the mixture was reflused at 80 ^{o}C. After refluxing for 6 h and cooling to room temperature, the mixture was concentrated under vacuum and chromatographed (silica 100 - 200 mesh; eluent: chloroform / methanol = 90 : 10). Yield: 85 %. The obtained compound was obtained and characterized by FT-IR (**Fig. S33**) and NMR spectrometer (**Fig. S34, S35, S36**). (Ref. 17)

FTIR (neat, cm^{-1}): 3453 (broad) (N-H stretching vibration), 2935(s) (C-H stretching vibration) 2937 (s) (C-H stretching vibration) 2933 (m) (C-H stretching vibration), 1738 (s) (C=O stretching vibration ester), 1641 (w) (N-H bending vibration), 1444 (m) (C-H bending vibration), 1367 (m) (C-H bending vibration), 1231 (s) (P=O stretching vibration), 1176 (m) (C-N stretching vibration), 1099 (m) (C-O stretching vibration), 1061 (m) (C-O stretching vibration), 1032 (C-O stretching vibration);

^{1}H (300 MHz, $CDCl_3$) δ 4.11 - 3.99 (m, 5H), 3.57 (s, 3H), 2.89 - 2.79 (m, 1H), 2.23 - 2.20 (t, J = 7.5 Hz, 2H), 1.55 (bs, 1H), 1.52 (bs, 3H), 150 (bs, 2H), 1.39 - 1.10 (bs, 14H), 0.98 (bs, 8H);

^{13}C (75 MHz, $CDCl_3$): δ 174.2, 77.3, 62.7, 62.6, 61.5, 61.4, 51.5, 51.3, 50.5, 48.4, 34, 33.7, 29.8, 29.5, 29, 26.0, 25.9, 24.8, 16.5.

^{31}P (121 MHz, $CDCl_3$): δ 29.02.

Reference:

17. Azizi, N. & Saidi, M. R. Synthesis of tertiary a-amino phosphonate by one-pot three component coupling mediated by LPDE. Tetrahedron **59**, 5329–5332 (2003).

Figure S33. FT - IR spectrum of aminophosphonates.

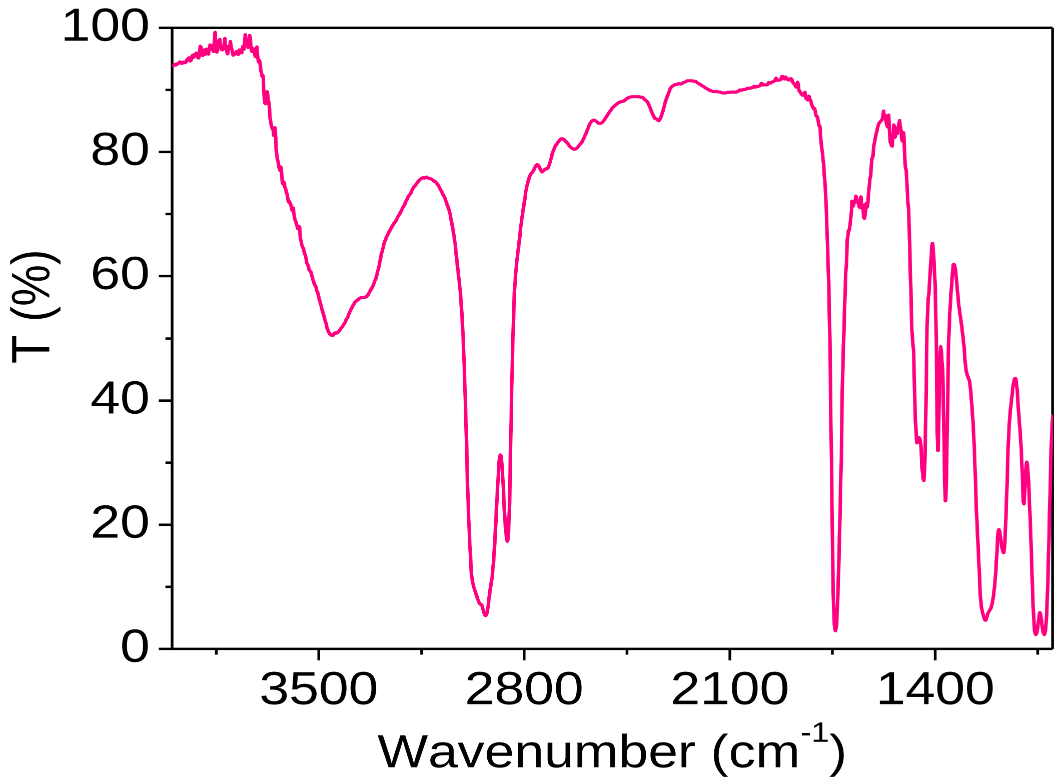

Figure S34. ^{1}H NMR spectrum of aminophosphonates

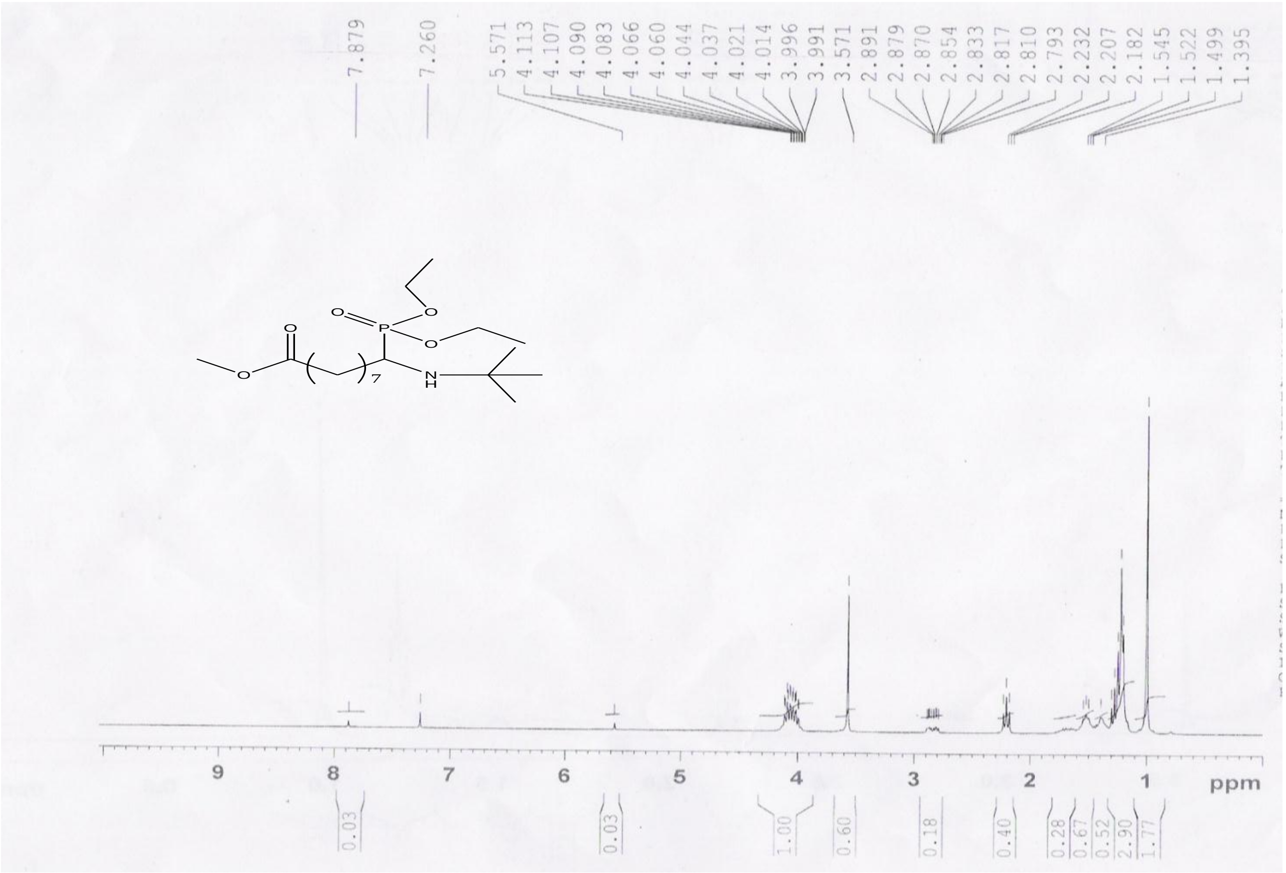

Figure S35. ^{13}C NMR spectrum of aminophosphonates

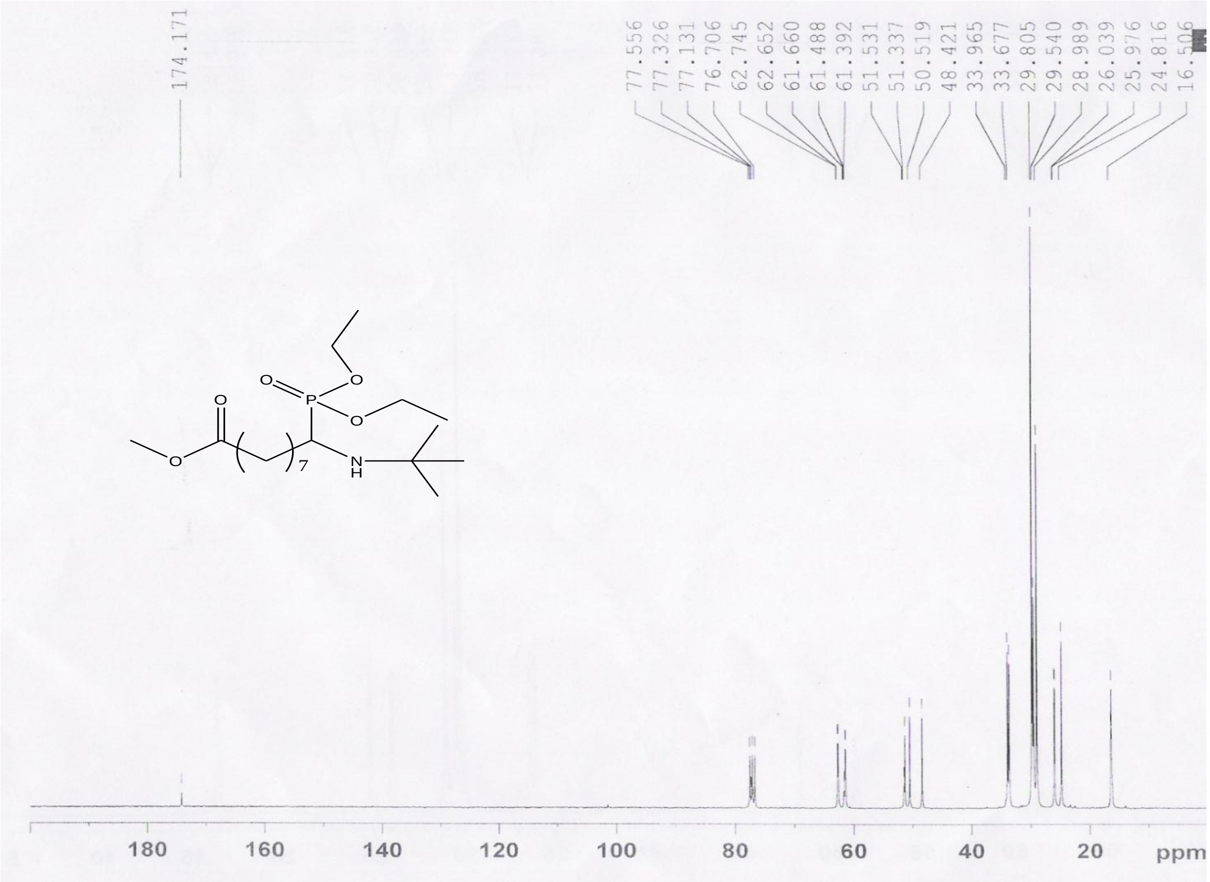

Figure S36. ^{31}P NMR spectrum of aminophosphonates functional group containing compound

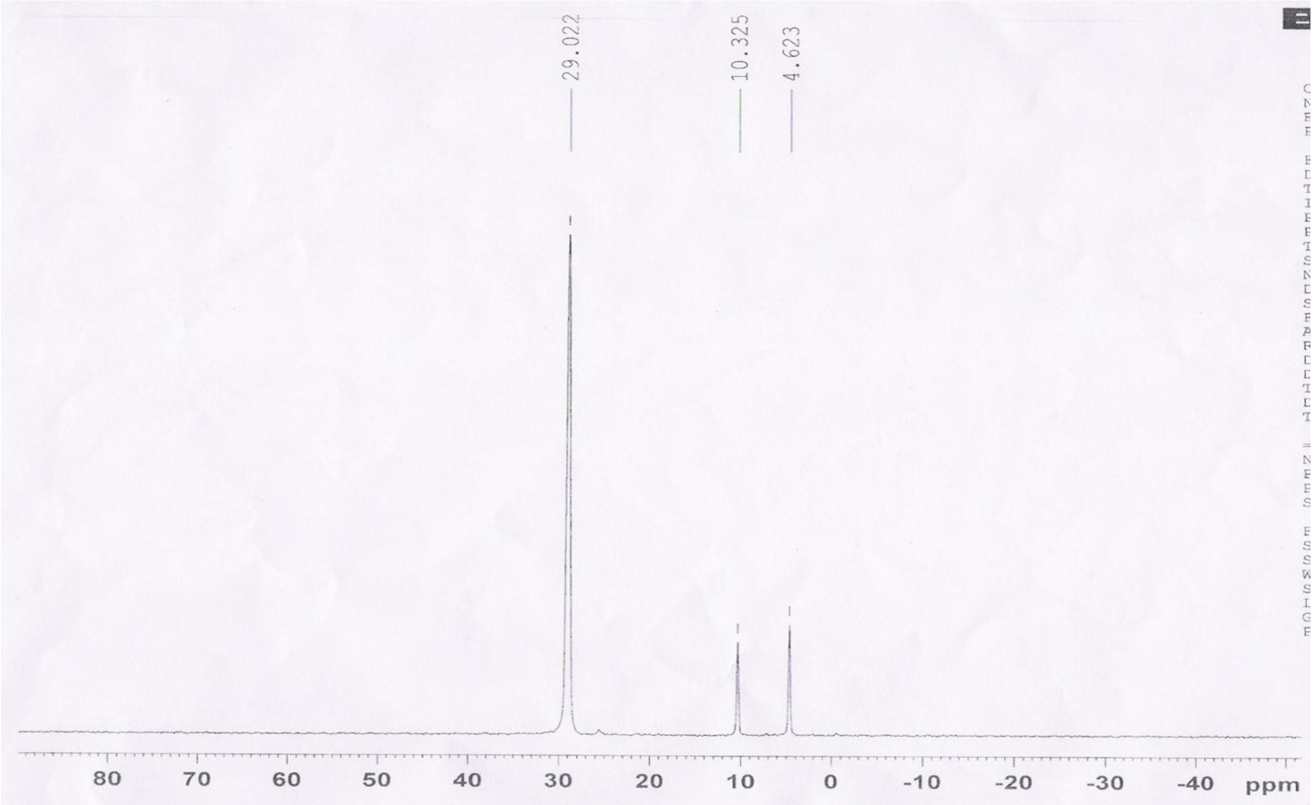

B. Functionalisation of Fe_3O_4 particles with aminophosphonate group.

B.1 Ozonolysis of the particles

To a three necked flask having Fe_3O_4 particle (5 g, 16.86) dispersed in dichloromethane (20 mL) cooled with blowing of oxygen gas till - 40 ^oC, ozone gas was bubbled. When solution became less dense, about 1 g of triphenyl phosphine was added with oxygen gas blowing only. The particle was kept stirring for overnight, centrifuged and washed with ethyl acetate three times to remove the excess triphenyl phosphine and triphenyl phosphine oxide. The obtained particle was characterized by FTIR (**Fig. S37**). But no change at the FT-IR spectra of Fe_3O_4 particles was observed except losing of peak corresponding to sp^2 C-H stretching vibration and the smell of the aldehyde. This confirmed the conversion of C-C double bond of the oleate ligand to the corresponding aldehyde functional group. It is also known from the literature that there is no much effect in the properties of the Fe_3O_4 magnetic nanoparticles by ozone gas. (Ref. 18)

FTIR (KBr, cm^{-1}): 2921 (s) (C-H stretching vibration), 1700 (b, w) (C=O stretching vibration of -CHO), 1544 (w) (asymmetric $–COO^-$ stretching vibration), 1460 (w) (C-H bending vibration), 1369 (w) (symmetric $–COO^-$ stretching vibration).

Reference:

18. Lee, S. Y. & Harris, M. T. Surface modification of magnetic nanoparticles capped by oleic acids: Characterization and colloidal stability in polar solvents. Journal of Colloid and Interface Science. **293** , 401–408 (2006).

Figure S37. FT-IR spectrum of the Fe_3O_4 particles obtained after the ozonolysis

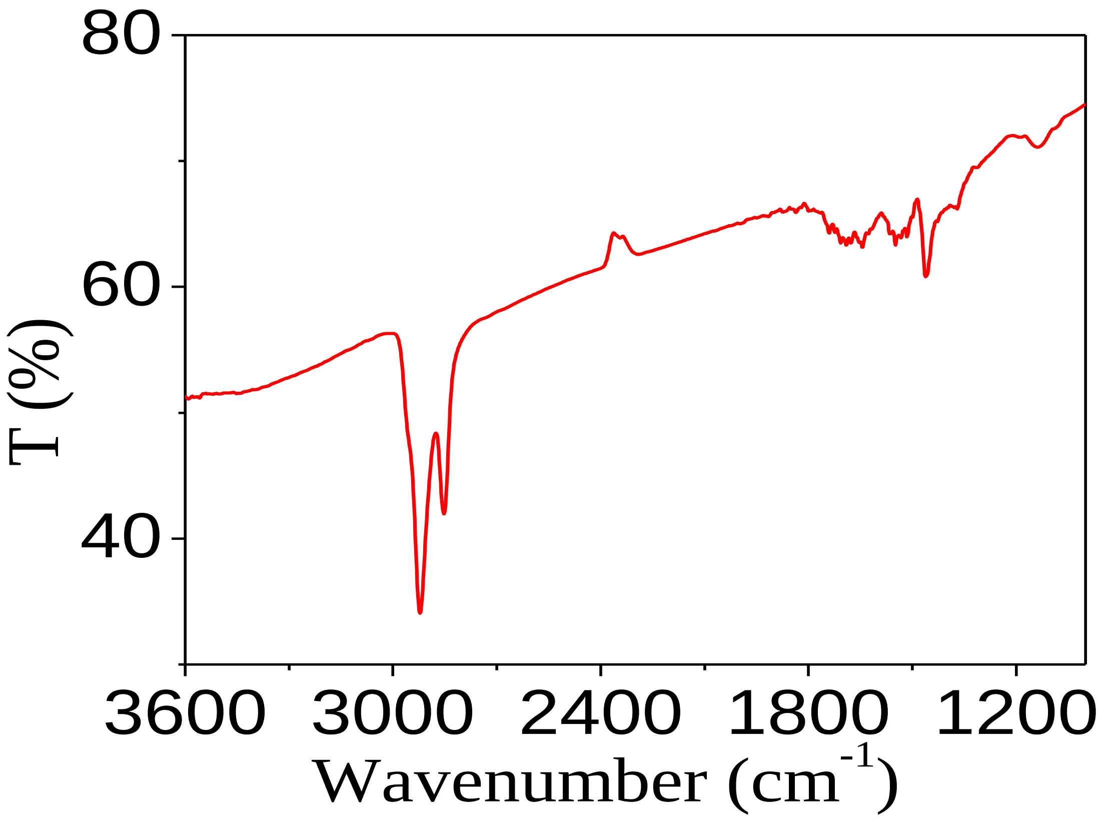

B.2 Functionalization of the particles by aminophosphonate group

About 200 mg of the aldehyde functionalized particles was dissolved in 4 mL of acetonitrile. Tertiary butyl amine (1 equivalent) was added and stirred vigorously for 15 min. Then diethyl phosphite (about 1 equivalent) was added to the reaction mixture and refluxed at the temperature of 80 ^{o}C. At first the particles were not dispersible but after heating for around 3 h., it was observed to disperse in acetonitrile. After heating for 6 h, the particles were dried under reduced pressure, washed and centrifuged with methanol for three times. The particles were characterized with FT-IR spectrometer (**Fig. S38**).

FT-IR (KBr, cm^{-1}): 3418 (b) (N-H stretching vibration), 2978 (s) / 2931 (s) (C-H stretching), 1667 (m) (N-H bending vibration), 1613 (m) (asymmetric –COO stretching vibration), 1447 (m) (C-H bending vibration), 1394 (m) (C-H bending vibration), 1246 (m) (P=O stretching vibration), 1175 (m) (C-N stretching vibration), 1050 (s) (C-O stretching vibration), 795 (m) (N-H loop), 551 (F-O stretching vibration).

Figure S38. FT-IR spectrum of aminophosphonate functionalized Fe_3O_4 particles

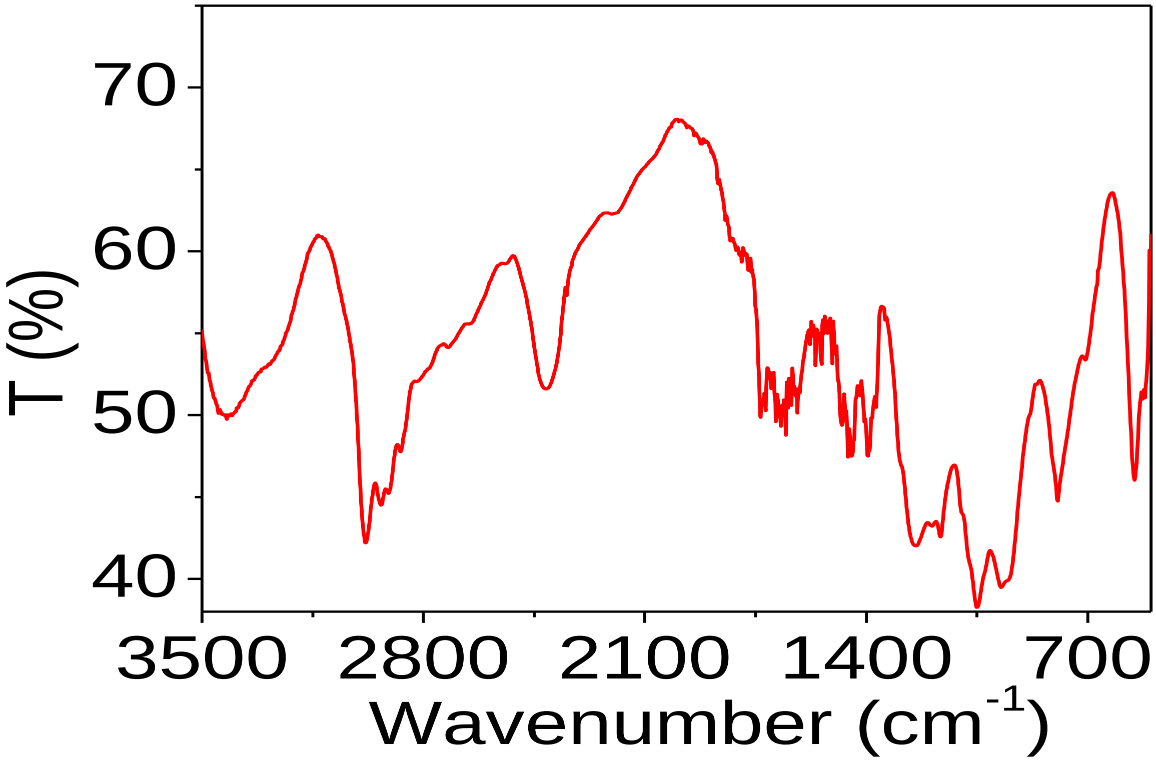

7.5. Stablity study in distilled water

We have carried out zetapotential measurement of amino-phosphonate functionalized particles MNPS. It is found to be -38.8 ± 0.6 mV (Fig. S39). The hydrodynamic size varies from 97 to 101 nm when DLS measurements were carried out within 60 min at different time intervals. It is suggested that particles are stable up to 60 min.

Figure S39: Plot of (a) Intensity (kcps) vs Zeta Potential (mV) of the MNPs- aspartate dispersed in water at pH- 8 and (b) Weighted Z-average hydrodynamic diameter (nm) vs Time (min.) of the sample in water.

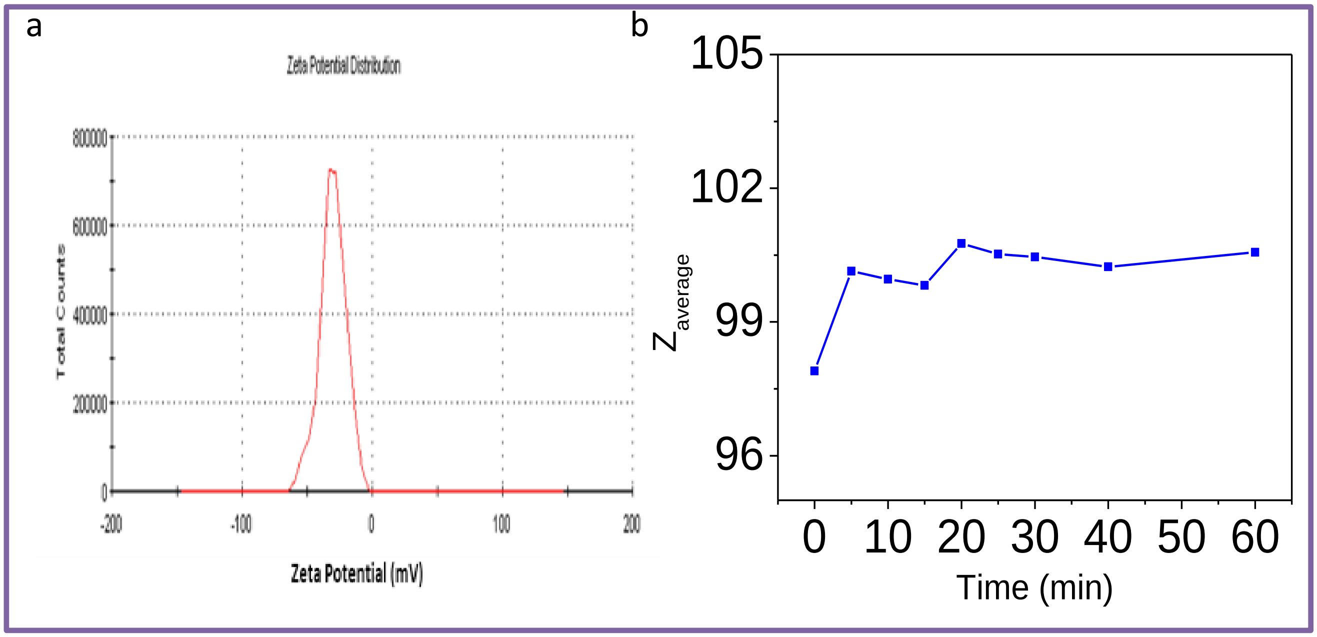

SI 7.6. Stability study in cell culture media

The zeta – potential measurement of MNP-asparatate was carried out. Its values in the DMEM media with serum is -0.03 mV. From the stability chart, the hydrodynamic size of MNP-asparatate in DMEN medium with serum is ranging from 117 nm to 125 nm when the DLS measurement was carried out for 3 h at different time intervals.

Figure S40: Plot of (a) Intensity (kcps) vs Zeta Potential (mV) of the MNPs- aspartate dispersed in DMEM with serum and (b) Weighted Z-average hydrodynamic diameter (nm) vs Time (min.) of the sample in DMEM with serum.

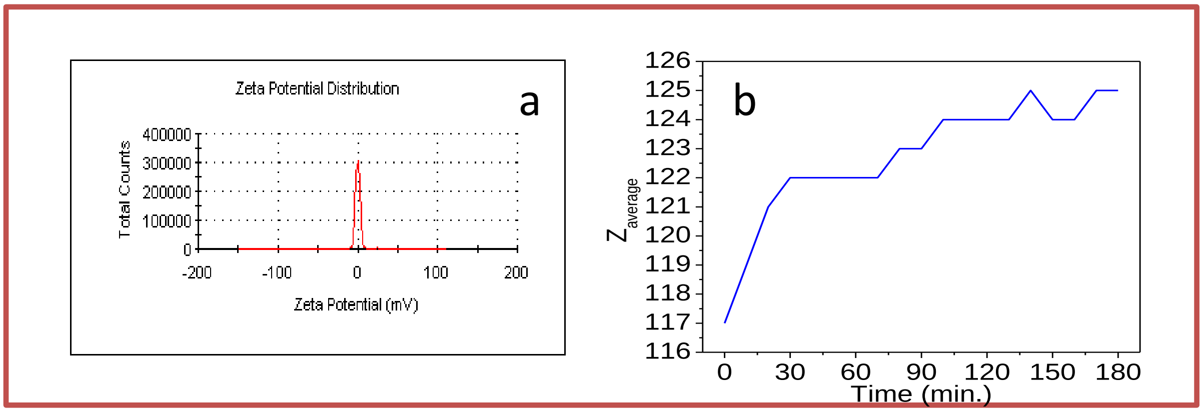

SI 7.7. Test

Fig. S41. Prussian blue test of control (A), MNPs -Aspartic acid (B), MNPs -Aminophosphonate (C) & MNPs- thioglycolysis acid (D)

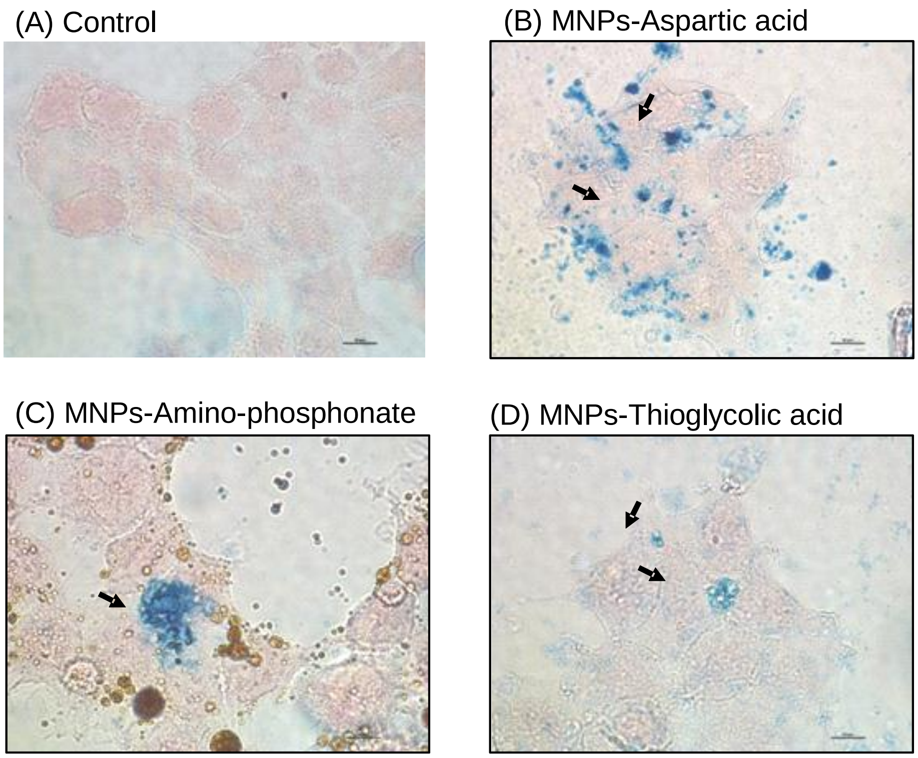

Fig. S42 The histogram analysis of flow cytometry

Sub-G1 analysis by PI staining and flow cytomtery

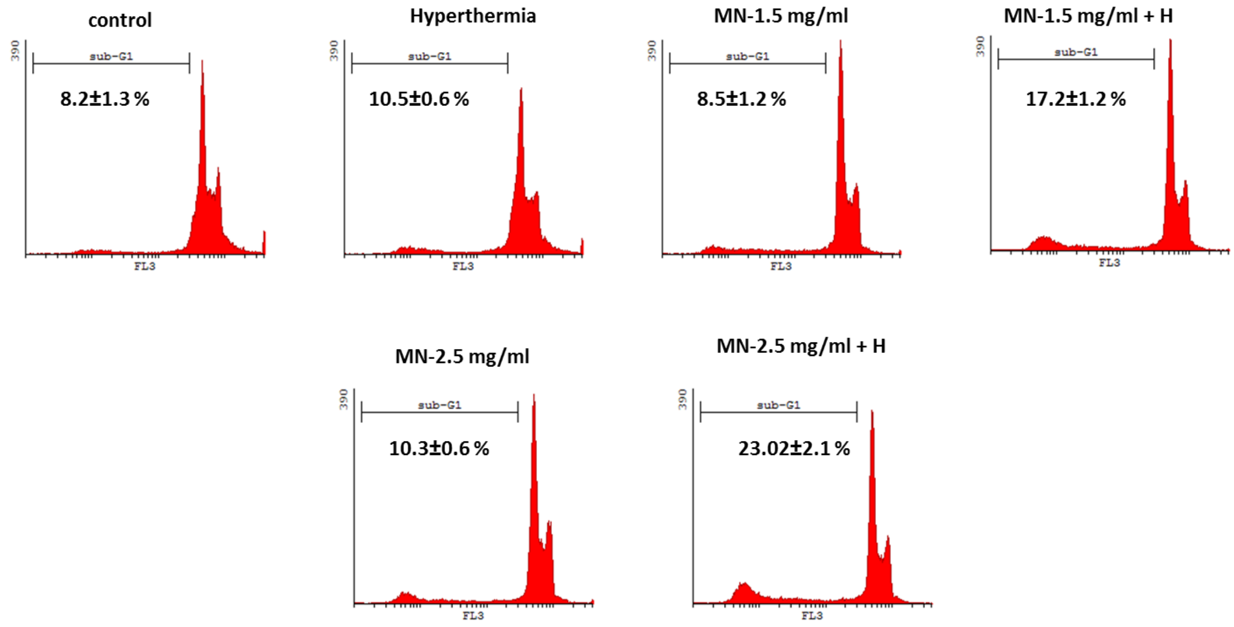

SI 7.8. Labeling of MNPs with FITC

To ethylene diamine (1 ml, 15 mmol.) in dried dimethyl sulfoxide (5 ml) contained in a 25 ml corning round bottom flask, 1.4 mg (3.5 mmol.) of FITC was added and stirred for overnight. The excess ethylene diamine was removed under pressure. To 10 mg of MNPs-aspartate dissolved in dried dimethyl formamide in a separate corning round bottom flask at 0 ^{o}C, diisopropyl carbodiimide (1 ml) (6 mmol.) was added. After stirring for twenty minutes, 0.5 ml of the reaction mixture of FITC-ethylene diamine was added and stirred for overnight. The particles were purified by repeated centrifuge and washing by ethanol and water mixture. The particles were again purified by dialysis using a dialysis tubing membrane for two days. The particles were collected by centrifuge, dried and stored at 0 ^{o}C.
